# A study on the factors influencing the vulnerability of women of childbearing age to health poverty in rural western China

**DOI:** 10.1038/s41598-024-64070-z

**Published:** 2024-06-08

**Authors:** Ximin Ma, Qi Hu, Jiahui He, Chunsheng Li, Mingsha Song, Youyun Wang, Hui Qiao

**Affiliations:** 1 School of Public Health, Ningxia Medical University, No. 1160 Shengli Street, Xingqing District, Yinchuan, 750004 Ningxia China; 2 Key Laboratory of Environmental Factors and Chronic Disease Control, Yinchuan, 750004 China; 3 School of Humanities and Management, Ningxia Medical University, Yinchuan, 750004 China

**Keywords:** Women of childbearing age, Health poverty, Shapley decomposition, Vulnerability to health poverty, Panel data, Epidemiology, Risk factors

## Abstract

The health of women of childbearing age in rural areas is crucial for the development of individuals, families, and society. Research on the identification and influencing factors of health vulnerability in impoverished and disadvantaged groups is important for adjusting and implementing health poverty alleviation policies. However, there is limited research on the health vulnerability of women of childbearing age in rural Western China. Based on panel data from the Rural Residents' Family Health Status Survey in 2019 and 2022, the vulnerability to health poverty of women of childbearing age in rural areas was constructed using the three-stage feasible generalized least squares method. Variables from four dimensions—physical capital, financial capital, social capital, and human capital—were included in the sustainable livelihood analysis framework for analysis. The Tobit model was used to analyze the influencing factors of vulnerability to health poverty among women of childbearing age in rural Western China, and the contribution rates of various factors were studied using the Shapley value decomposition method. In 2019 and 2022, under the poverty line standards of $1.90 and $2.15, respectively, the vulnerability to health poverty among rural women of childbearing age exceeded 20%. Tobit regression analysis revealed that the type of drinking water being well water significantly increased the vulnerability to health poverty of rural women of childbearing age (*P* < 0.05), whereas the separation of housing and kitchen, registered poor households, household loans, annual per capita household income, expenditures on social interactions, educational level, self-assessed health status, respondent age, and the utilization of hospital services significantly reduced the vulnerability to health poverty of rural women of childbearing age (*P* < 0.05). Shapley's decomposition shows that annual per capita household income, expenditures on social interactions, respondent age, and household loans are the factors contributing most to the vulnerability to health poverty of rural women of childbearing age, while other variables have a smaller contribution rate. The health poverty situation of women of childbearing age in rural Western China is not optimistic. Preintervention for health poverty should be strengthened among rural women of childbearing age, early warning mechanisms for the risk of falling back into poverty due to illness should be established, the precise identification of highly vulnerable rural women of childbearing age should be improved, and the medical insurance system for rural women of childbearing age should be enhanced to help improve their current health poverty situation.

## Introduction

Eliminating poverty is widely recognized as one of the most pressing challenges of the twenty-first century^1^. According to data released by the World Bank in 2018, a substantial portion of the global population, totaling 736 million individuals, continues to live on less than $1.90 per day. The eradication of poverty is one of the most significant challenges facing humanity. It prominently features one of the 17 Sustainable Development Goals (SDGs) outlined by the United Nations, with the primary objective of eliminating all forms of poverty by 2030^2,3^. China, a developing country that previously had the highest number of rural individuals living in poverty worldwide, has demonstrated considerable concern for this issue^4^. Since the initiation of its reform and opening-up policy in 1978, China has made remarkable progress in addressing poverty, ultimately attaining a comprehensive victory over destitution by 2020^5^. This accomplishment has not only had a profound domestic impact but also made a significant global contribution to the battle against poverty. Recent data from China revealed that by the conclusion of 2020, an impressive 98.99 million individuals residing in rural regions were successfully lifted out of poverty^6–9^. This milestone signifies the fulfillment of the objective of eliminating poverty and establishing a comprehensive and prosperous society for the new era.

Although China has eliminated poverty, poverty is dynamic, and the current nonpoverty status does not mean that it will continue in the future. Some nonpoor households/individuals may face the risk of falling into poverty or returning to poverty due to job loss or health issues. As of the end of 2019, a total of 980,000 households, comprising approximately 2.66 million individuals, remained in poverty in China^10^. Among the surveyed households, a significant proportion of 375,000 (equivalent to 968,000 individuals) were found to be trapped in poverty primarily due to illness. This group accounted for 42.2% of all households living in poverty^11^. From 2016 to 2019, there was a consistent prevalence of illness-induced impoverishment, with rates of approximately 40%. This underscores the significant impact of health-related issues on the occurrence of poverty. The primary focus and intricacy of efforts to address poverty lie in addressing health-related deprivation. As a result, it is crucial to evaluate the future vulnerability of these populations to health-related shocks and utilize predictive models to measure the likelihood of falling into poverty. These factors are essential for reducing the vulnerability of rural communities to health poverty and promoting efficient governance in the fight against contemporary poverty.

In the context of the migration of rural youth to urban areas for employment opportunities^11^, women have become a crucial part of the labor force in rural households. The China Health Services Survey has been conducted six times since 1993, revealing a notable improvement in healthcare utilization among women of childbearing age in China over the past decade. However, rural women in this age group continue to experience substandard health conditions, particularly in western rural regions where this problem is more pronounced^12^. The prevalence of poverty among women of childbearing age is a widespread issue influenced by various factors, including physical, psychological, and environmental factors. This situation leads to different forms of impoverishment, such as health poverty, educational poverty, and economic poverty^13,14^. Given the remote and isolated nature of the majority of rural areas in western China, coupled with the limited accessibility of medical resources and the low level of health awareness among women of childbearing age, as well as their numerous responsibilities for family care and demanding work, rural women in this demographic group are particularly prone to experiencing health poverty^15^. Consequently, women who reside in rural areas and who are of childbearing age play a pivotal role in promoting the revitalization of rural communities and bolstering efforts aimed at eradicating poverty in the postpoverty alleviation era.

To sustain the gains from poverty eradication and prevent women of reproductive age in rural China from falling into the risk of returning to poverty due to illness, it is necessary to assess the vulnerability of rural residents to health poverty from a prospective perspective and to further explore the main determinants of vulnerability to health poverty. Health poverty is defined as the condition in which individuals' health statuses are below a defined threshold considered minimally acceptable. This threshold can be based on various health indicators, such as life expectancy, the prevalence of certain diseases, or overall health status^16^, whereas vulnerability to health poverty is an expansion of health poverty and refers to the probability of an individual's or household's future level of well-being falling below the poverty line as a result of health-related risks/shocks and is characterized as dynamic and forward-looking^17,18^. Vulnerability to health poverty can therefore be used as a risk factor or early warning signal for the likelihood that an individual will be impoverished in the future as a result of health-related problems.

Previous studies have primarily examined the impact of single factors such as economics, education, and poverty alleviation policies on health-related vulnerability to poverty^19,20^. Few studies have adopted a multidimensional approach through the lens of sustainable livelihood frameworks, and these studies have largely focused on elderly individuals and patients with chronic diseases ^21–24^. Research specifically addressing health-related vulnerability to poverty among women of childbearing age in rural areas is scarce. Therefore, this study utilizes the four dimensions of the sustainable livelihood framework—physical, financial, social, and human capital^25^—to accurately identify the risks of impoverishment due to health issues among childbearing-aged women in rural Western China. This research provides a comprehensive analysis of the determinants of health-related poverty vulnerability, offering microlevel data support for proactive measures to improve the health of rural women of childbearing age, thereby contributing to poverty reduction and sustainable development efforts.

This study focused on married women of childbearing age in rural Western China to explore their health-related vulnerability to poverty and the influencing factors. This research aims to address the following questions: (1) What is the current status of health-related poverty vulnerability among these women? How can those at high risk be accurately identified to reduce the likelihood of poverty and sustain poverty alleviation achievements? (2) By constructing a sustainable livelihood analytical framework, this study examines the factors influencing health-related poverty vulnerability by applying the Shapley decomposition method to identify key factors in rural Western China. This research enriches the theoretical discourse on poverty vulnerability from a health perspective, utilizes a sustainable livelihood framework as the theoretical basis, and provides empirical evidence for preventing health-related impoverishment and postpoverty alleviation in China. It also offers theoretical support for health poverty alleviation efforts in other regions of China and other developing countries.

## Methods

### Study design and sample

The data for this study were collected from a pilot project on 'Innovative Payment Systems to Enhance Healthcare Efficiency,' jointly conducted by Harvard University and Ningxia Medical University in 2009, 2011, and 2012^26,27^ (https://www.hsph.harvard.edu/re-alignment-health-system-incentives/sample-page/policy-impact-and-media-coverage/). Subsequent follow-up surveys were funded by the National Natural Science Foundation of China in 2015, 2019, and 2022. This research utilized follow-up data from the 'Rural Household Health Inquiry Survey' conducted in 2019 and 2022, which aimed to comprehensively assess health status and healthcare service utilization among residents in rural areas of Ningxia, Western China. The survey provides empirical data and scientific evidence to inform the development of precise and realistic healthcare policies.

A multistage sampling technique was employed to conduct surveys in four counties of Ningxia: Haiyuan, Yanchi, Pengyang, and Xiji. In the first stage, 208 administrative villages from 53 townships across these four counties were classified into three levels of economic development: high, medium, and low. In the second stage, simple random sampling was used to select 40% of the villages from each township. This involved assigning numbers from 1 to 208 to each village and using a random number table to select the required percentage of villages. Numbers outside the designated range or duplicates were excluded until the quota was met. In the third stage, systematic sampling was utilized in each sampled village to select one household for every five, resulting in a total of 20–33 households per village.

In this survey, a total of 21,300 questionnaires were distributed, and all were retrieved, yielding an effective response rate of 97.75% with 20,821 valid questionnaires. The study focused on women who had been residing at home for more than six months, who were married, and who were aged between 18 and 49 years. After excluding samples with missing or invalid values, 5605 eligible rural women of childbearing age were included in the study.

The formula for calculating the sample size of count data in cross-sectional studies is $n=\frac{{u}_{\alpha }^{2}\pi \left(1-\pi \right)}{{\delta }^{2}}$. The significance test level α = 0.05 is usually adopted, and the allowable error $\updelta = 0.1\uppi$ is considered to be general. In 2019, 53.7% of rural residents in Ningxia were projected to fall into poverty due to future health issues, with π = 53.7%. Thus, the required sample size is approximately 332 individuals. ^28^, The subjects included in this study met the sample size requirements.

### Vulnerability to health poverty measurement

Vulnerability to health poverty is a predictive indicator of the likelihood that an individual's or household's future level of well-being will fall below the poverty line as a result of health-related risks/shocks. The prevailing approach for measuring this vulnerability is through the use of vulnerability as expected poverty (VEP)^29,30^, which employs a three-stage feasible generalized least squares (FGLS) method to assess the extent to which a family is susceptible to health-related poverty. This method involves a series of three steps to quantify a family's vulnerability to health poverty^31^:

Initially, the ordinary least squares (OLS) method is employed to estimate the income equation:

1 $$\text{ln}{Y}_{it+1}=\beta {X}_{it}+{e}_{it}$$

where ${Y}_{it+1}$ refers to the income level of the rural population in period $T+1$ and ${X}_{it}$ refers to a series of observable variables that affect the family income level, including family demographic characteristics, health risk variables, family resource endowment variables, risk response strategies, and health support system variables. To construct indicators for vulnerability to health poverty, this study integrates health risk theory and existing variables from the database to select indicators such as two-week morbidity rates, two-week bedridden rates, two-week work absence rates, hospitalization rates, and catastrophic health expenditures to construct indicators of vulnerability to health poverty. Considering the heterogeneity of the rural population in different counties, townships, and villages, the residual square is regarded as the approximate value of income variance $\hat{e} 2i$, and the residual square is used as the explained variable to construct the regression model of residual square $\hat{e} 2i$ for individual characteristics:

2 $${\hat{e}}_{i}^{2}=\theta \times {X}_{i}+{\eta }_{i}$$

The estimated value and the residual estimated value of ${Y}_{it+1}$ can be obtained through formulas (1) and (2). Second, the heteroscedasticity structure is constructed as a weight for weighted regression, and the expected value (3) and variance (4) of the future income logarithm are estimated:

3 $$\hat{E}[\text{ln}{Y}_{i}\mid {X}_{i}]={X}_{i}\hat{\beta }$$

4 $$\hat{V}[\text{ln}{Y}_{\text{i}}\mid {X}_{\text{i}}]={\hat{\sigma }}_{ei}^{2}={X}_{i}\hat{\theta }$$

Finally, the poverty line is selected to estimate vulnerability to poverty. This study used the international poverty lines of $1.9/day and $2.15/day as the poverty lines for measuring vulnerability to poverty^32,33^. Rural women of childbearing age with a score of ≥ 0.5 were categorized as having vulnerability to health poverty, and those with a score of < 0.5 were categorized as not having vulnerability to health poverty. The study population was rural women of childbearing age, so the lognormal distribution was more applicable. The logarithm of the poverty line in formula (5) is:

5 $$ \hat{v}_{i} = \overset{\lower0.5em\hbox{$\smash{\scriptscriptstyle\frown}$}}{P} ({\text{ln}}Y_{{\text{i}}} < {\text{ln}}l{\mid }X_{{\text{i}}} ) = \varphi \left( {\frac{{{\text{ln}}l - X_{{\text{i}}} \hat{\beta }}}{{\sqrt {X_{{\text{i}}} \hat{\theta }} }}} \right) $$

### Shapley decomposition

According to the concept of Shapley decomposition in poverty research, the contributions of various factors to vulnerability to health poverty are decomposed. Regression-based Shapley decomposition was proposed by Shorrocks^34^. By using Shapley values to decompose indicators based on regression models, it is possible not only to examine which factors determine the dependent variable but also to quantify the contribution of these factors to the dependent variable.

According to the relevant literature, vulnerability to health poverty is indicated by income levels and income disparities relative to a specific poverty line^35^. That is,

$${V}_{h}=V\left({\mu }_{h},{\sigma }_{h}^{2}\right)$$

Then, the vulnerability deviation of household h relative to the reference household can be represented as

$${V}_{h}-{V}_{m}=V\left({\mu }_{h},{\sigma }_{h}^{2}\right)-V\left({\mu }_{m},{\sigma }_{m}^{2}\right)$$

Next, according to the calculation of

$${V}_{hm}=V\left({\mu }_{h},{\sigma }_{m}^{2}\right)\text{ and }{V}_{mh}=V\left({\mu }_{m},{\sigma }_{h}^{2}\right)$$

The effects of differences in means and variances were identified. ${V}_{hm}$ refers to the vulnerability level determined by taking the real family mean as the mean value and the reference family variance as the variance. ${V}_{mh}$ refers to the vulnerability level by taking the reference family mean as the mean value and the actual family variance as the variance.

Finally, according to the relevant literature, decomposition is performed using the following equation:

$${V}_{h}-{V}_{m}=\left[\left({V}_{hm}-{V}_{m}\right)+\left({V}_{h}-{V}_{mh}\right)\right]/2+\left[\left({V}_{h}-{V}_{hm}\right)+\left({V}_{mh}-{V}_{m}\right)\right]$$

The first term on the right reflects the impact of the mean difference, while the second term reflects the impact of the variance difference.

### Theoretical framework and main variables

Considering that the vulnerability of rural women of childbearing age to health poverty is influenced by various factors, this study adopted the sustainable livelihoods analysis (SLA) theoretical framework^36,37^. The SLA framework identifies livelihood capital as its core component, and it comprehensively reveals rural residents' ability to cope with risks. Under the SLA framework, livelihood capital is divided into four dimensions: physical capital, financial capital, social capital, and human capital^38^. This research explores the key factors affecting the vulnerability of rural women of childbearing age to health poverty based on the SLA framework.

Physical capital is measured through the type of housing, type of drinking water, type of toilet, and separation of housing and kitchen. Financial capital is assessed by the registered poor household, household loans, and annual per capita household income. Social capital is measured by expenditures on social interactions. Human capital is evaluated based on education, self-assessed health status, chronic illness status, outpatient service utilization, inpatient service utilization, age, and occupation of the respondents. In social capital, social interactions refer to the investment behavior among members of the social network and its resources. This includes expenses incurred during various events among relatives and friends within the year, such as weddings, downries, New Year gifts, wedding gifts, funeral expenses, and costs of gifts for relatives and friends^39,40^. In terms of financial capital, to investigate the differences in average annual income per capita among different families, this study classifies the average annual income per family. This study uses the internationally recognized quintile method to divide income into five different categories: I, II, III, IV, and V^41^. Detailed definitions of the main variables can be found in Table 1.

**Table 1:** Variable definitions and descriptive statistics.

Variables	Definition	2019		2022	
		Mean	Standard deviation	Mean	Standard deviation
Explained variable (Y)					
Vulnerability to health poverty	Measure with expected poverty vulnerability (VEP)				
Explanatory variable (X)					
Physical capital					
Type of housing	Unordered multi categorical variable, 1 = brick soil concrete, 2 = brick wood, 3 = Civil Engineering, 4 = full brick	2.6116	1.1382	2.8336	1.1680
Type of drinking water	Unordered multi categorical variable, 1 = tap water,2 = cellar water, 3 = well water	1.2449	0.4922	1.0420	0.2467
Type of toilet	Unordered multi categorical variable, 1 = water flushing type, 2 = toilet, 3 = dry toilet	2.8187	0.5074	2.7941	0.5644
Separation of housing and kitchen	Categorical variable, Yes = 1 and No = 0	0.6607	0.4736	0.7286	0.4448
Financial capital					
Registered poor household	Categorical variable, Yes = 1 and No = 0	0.4854	0.4999	0.5173	0.4998
Household loans	Categorical variable, Yes = 1 and No = 0	0.7443	0.4363	0.6404	0.4800
Annual per capita household income	Unordered multi categorical variable ,I group = 1, II group = 2, III group = 3, IV group = 4, V group = 5	2.9556	1.4054	2.9559	1.4160
Social capital					
Expenditures on social interactions(log)	Continuous variable (logarithm)	8.2136	1.4238	8.0187	1.4486
Human capital					
Educational level of the respondents	Unordered multi categorical variable ,1 = no schooling, 2 = primary school, 3 = junior high school, 4 = senior high school or above	2.1527	0.9249	2.2241	0.9447
Self-assessed health status of respondents	Ordered multi categorical variable ,1 = very good, 2 = good, 3 = average, 4 = poor, 5 = very poor	2.4511	0.8440	2.6281	1.0054
Chronic disease of the respondents	Categorical variable, Yes = 1 and No = 0	0.1489	0.3560	0.1264	0.3324
Outpatient service utilization of the respondents	Categorical variable, Yes = 1 and No = 0	0.0667	0.2496	0.0329	0.1785
Inpatient service utilization of the respondents	Categorical variable, Yes = 1 and No = 0	0.1070	0.3092	0.0943	0.2923
Age of the respondents	Continuous variable	36.4703	8.3725	38.0408	8.1026
Occupation of the respondents of the respondents	Unordered multi categorical variable, Farming = 1, working = 2, unemployed = 3	1.4781	0.7064	1.3839	0.6270

### Data analysis

First, we use means and standard deviations to characterize the basic characteristics of rural women of childbearing age. Second, following the VEP approach described above, a three-stage feasible generalized least squares model is used to estimate the vulnerability to health poverty of rural women of childbearing age in 2019 and 2022. Moreover, the accuracy of predicting vulnerability to health poverty was tested by comparing the incidence of vulnerability to health poverty with the actual poverty incidence in 2019 and 2022 using a poverty transfer matrix. The influencing factors of vulnerability to health poverty were analyzed using Tobit regression. Additionally, the contribution of livelihood capital elements to vulnerability to health poverty was decomposed based on Shapley. Statistical analysis was performed using econometric software, specifically SPSS 26.0 and STATA version 17.0.

### Ethics approval and consent to participate

Ethical approval was Granted by the Ethics Committee of Ningxia Medical University, approval number no. 2021-G152. All the participants provided signed informed consent at the time of participation. The study methodology was carried out in accordance with approved guidelines.

## Results

### Incidence of poverty and incidence of vulnerability to health poverty among rural women of childbearing age

Table 2 shows the incidence of poverty and vulnerability to health poverty among rural women of childbearing age under different poverty line standards in 2019 and 2022. In 2019, when $1.90 was used as the poverty line, the poverty incidence was 21.8%, and the vulnerability to health poverty incidence was 21.7%; when $2.15 was used as the poverty line, the poverty incidence was 28.2%, while the vulnerability to health poverty incidence was 30.2%. In 2022, when $1.90 was used as the poverty line, the poverty incidence was 20.6%, and the vulnerability to health poverty incidence was 21.2%; when $2.15 was used as the poverty line, the poverty incidence was 27.0%, and the vulnerability to health poverty incidence was 27.0%.

**Table 2:** Comparison of the incidence of vulnerability to health poverty and the incidence of poverty among women of childbearing age in rural Ningxia, Western Region of China, 2019 and 2022.

Poverty line	2019 prevalence of poverty/incidence rate of vulnerability to health poverty (%)	2022 prevalence of poverty/incidence rate of vulnerability to health poverty (%)
Poverty line (1.9$)	21.8/21.7	20.6/21.2
Poverty line(2.15$)	28.2/30.2	27.0/27.0

Table 3 provides a detailed transfer matrix of the poverty incidence and vulnerability to health poverty rates among rural women of childbearing age under different poverty line standards in 2019 and 2022. In 2019, when $1.90 was used as the poverty line, 1.5% of the nonpoor population was vulnerable to health poverty; when $2.15 was used as the poverty line, 7.41% of the nonpoor population was vulnerable to health poverty. In 2022, when $1.90 was used as the poverty line, 0.68% of the nonpoor population was vulnerable to health poverty; when $2.15 was used as the poverty line, 7.76% of the nonpoor population was vulnerable to health poverty.

**Table 3:** Comparison of transfer matrices for the incidence of vulnerability to health poverty and the incidence of poverty among rural women of childbearing age in Ningxia, Western Region of China, in 2019 and 2022.

Poverty line	2019 (%)	2019		2022 (%)	2022	
		Vulnerability(%)	No vulnerability(%)		Vulnerability(%)	No vulnerability(%)
Poverty line (1.9$)	Poor	94.59	5.41	Poor	100.00	0.00
	Nonpoor	1.50	98.50	Nonpoor	0.68	99.32
Poverty line (2.15$)	Poor	88.47	11.53	Poor	86.88	13.12
	Nonpoor	7.41	92.59	Nonpoor	7.76	92.24

Under the poverty line of $1.9, there is no statistically significant difference in poverty (χ2 = 1.023, *P* = 0.312) or vulnerability to health poverty (χ2 = 0.230, *P* = 0.631) across different years. Under the poverty line of $2.15, there is no statistically significant difference in poverty (χ2 = 0.981, *P* = 0.322) across different years; however, the difference in vulnerability to health poverty across different years (χ2 = 6.828, *P* = 0.009) is statistically significant (specific results can be found in Tables 7 and 8 of the Supplementary Materials).

### Analysis of factors influencing the vulnerability of rural women of childbearing age to health poverty

Tables 4, 5 and 6 present the results of regression analyses considering survey sampling weights, while the results of regression analyses without considering sampling weights can be found in Tables 9 to 11 of the Supplementary Materials. The factors influencing the vulnerability of rural women of childbearing age to health poverty in 2019 and 2022 are shown in Table 4. Vulnerability to health poverty under the poverty line standards of $1.90 and $2.15 served as the dependent variable, and Tobit regression analysis was conducted on variables across four dimensions: physical capital, financial capital, social capital, and human capital. The results indicate that the type of drinking water being well water significantly increased the vulnerability of rural women of childbearing age to health poverty; factors such as separation of housing and kitchen, registered poor households, household loans, annual per capita household income, expenditures on social interactions, educational level, self-assessed health status, and the utilization of hospital services significantly reduced the vulnerability to health poverty of rural women of childbearing age.

**Table 4:** Analysis of risk factors for vulnerability to health poverty among rural women of childbearing age in 2019 and 2022.

Variable	2019				2022			
	Poverty line(1.9$)		Poverty line(2.15$)		Poverty line(1.9$)		Poverty line(2.15$)	
	Coefficient	SD	Coefficient	SD	Coefficient	SD	Coefficient	SD
Physical capital								
Type of housing (reference: brick soil concrete)								
Brick wood	− 0.0005	0.0035	− 0.0119	0.0098	0.0024	0.0012	0.0018	0.0022
Civil Engineering	0.0038	0.0026	− 0.0144	0.0096	− 0.0050	0.0022	− 0.0171**	0.0048
Full brick	− 0.0039	0.0043	− 0.0308	0.0131	0.0076***	0.0012	0.0066*	0.0022
Type of drinking water (reference: tap water)								
Cellar water	0.0133***	0.0008	0.0195***	0.0014	0.0305***	0.0017	0.0547***	0.0034
Well water	0.0223**	0.0051	0.0386**	0.0097	0.0584**	0.0147	0.1179**	0.0336
Type of toilet(reference: water flushing type)								
Toilet	− 0.0058	0.0073	− 0.0055	0.0150	0.0037	0.0019	0.0057*	0.0022
Dry toilet	0.0073**	0.0021	0.0184**	0.0036	0.0007	0.0020	0.0015	0.0023
Separation of housing and kitchen(reference: no)	− 0.0099**	0.0017	− 0.0144***	0.0014	0.0041***	0.0005	0.0073***	0.0006
Financial capital								
Registered poor household(reference:no)	0.0026	0.0022	− 0.0043**	0.0012	0.0067***	0.0008	0.0146***	0.0014
Household loans(reference:no)	− 0.0197***	0.0033	− 0.0183**	0.0035	− 0.0055**	0.0013	− 0.0151***	0.0014
Annual per capita household income(reference: I group)								
II group	− 0.8124***	0.0093	− 0.5291***	0.0089	− 0.8600***	0.0032	− 0.5351***	0.0044
III group	− 0.9791***	0.0028	− 1.0175***	0.0028	− 0.9852***	0.0013	− 0.9954***	0.0004
IV group	− 0.9706***	0.0031	− 1.0082***	0.0028	− 0.9793***	0.0017	− 0.9869***	0.0007
V group	− 0.9698***	0.0025	− 1.0056***	0.0043	− 0.9737***	0.0021	− 0.9773***	0.0020
Social capital								
Expenditures on social interactions(log)	− 0.0186***	0.0024	− 0.0242***	0.0027	− 0.0122***	0.0003	− 0.0189***	0.0004
Human capital								
Educational level of the respondents(reference: no schooling)								
Primary school	− 0.0096**	0.0025	− 0.0179 ***	0.0026	− 0.0075**	0.0016	− 0.0064*	0.0020
Junior high school	− 0.0128***	0.0005	− 0.0289***	0.0023	− 0.0067**	0.0015	− 0.0088	0.0039
Senior high school or above	− 0.0211**	0.0049	− 0.0441**	0.0102	− 0.0166**	0.0041	− 0.0242**	0.0059
Self− assessed health status of respondents(reference: very good)								
Good	− 0.0171***	0.0009	− 0.0204***	0.0014	0.0006	0.0012	− 0.0017	0.0025
Average	− 0.0196***	0.0025	− 0.0210**	0.0044	0.0090*	0.0031	0.0107	0.0048
Poor	− 0.0275***	0.0034	− 0.0262***	0.0031	0.0136**	0.0034	0.0179**	0.0046
Very poor	0.0018	0.0118	0.0296	0.0197	0.0195***	0.0023	0.0346***	0.0023
Chronic disease of the respondents(reference: no)	− 0.0035	0.0046	0.0005	0.0034	0.0057**	0.0018	0.0262***	0.0033
Outpatient service utilization of the respondents(reference: no)	0.0137	0.0078	0.0260*	0.0103	− 0.0056***	0.0006	− 0.0238***	0.0020
Inpatient service utilization of the respondents(reference: no)	− 0.0005	0.0006	− 0.0064**	0.0011	− 0.0123**	0.0038	− 0.0187*	0.0059
Age of the respondents (reference:)	− 0.0005*	0.0002	− 0.0009***	0.0001	− 0.0008***	0.0001	− 0.0013***	0.0002
Occupation of the respondents of the respondents (reference: Farming)								
Working	− 0.0009	0.0030	− 0.0045	0.0034	− 0.0022**	0.0004	− 0.0043*	0.0015
Unemployed	− 0.0049	0.0032	− 0.0077	0.0048	0.0024	0.0012	0.0018**	0.0022

***P < 0.01; **P < 0.05; *P < 0.1.

**Table 5:** Decomposition of risk factors for vulnerability to health poverty among rural women of childbearing age in 2019 and 2022.

Variable	2019				2022			
	Poverty line (1.9$)		Poverty line (2.15$)		Poverty line (1.9$)		Poverty line (2.15$)	
	Shapley	Contribution (%)	Shapley	Contribution (%)	Shapley	Contribution (%)	Shapley	Contribution (%)
Type of drinking water	0.0392	0.14	0.0379	0.16	0.3625	1.03	0.2447	0.86
Separation of housing and kitchen	0.1925	0.68	0.1406	0.59	0.4753	1.35	0.3761	1.33
Registered poor household	0.2498	0.88	0.1752	0.74	0.9050	2.57	0.7252	2.56
Household loans	2.0239	7.12	1.5663	6.62	1.2070	3.43	0.9512	3.36
Annual per capita household income	12.9714	45.65	11.3310	47.92	11.9878	34.11	10.5086	37.12
Expenditures on social interactions(log)	9.8124	34.53	7.9678	33.70	11.3365	32.25	9.0171	31.85
Educational level of the respondents	0.3969	1.40	0.2888	1.22	0.6845	1.95	0.5104	1.80
Self-assessed health status of respondents	1.4198	5.00	1.1032	4.67	0.0895	0.25	0.0769	0.27
Inpatient service utilization of the respondents	0.0978	0.34	0.0810	0.34	0.4185	1.19	0.3431	1.21
Age of the respondents	1.2090	4.25	0.9536	4.03	4.2564	12.11	3.3109	11.69
TOTAL	28.4168	100.00	23.6454	100.00	35.1480	100.00	28.3117	100.00

**Table 6:** Decomposition of different dimensions of vulnerability to health poverty.

Dimension	2019				2022			
	Poverty line (1.9$)		Poverty line (2.15$)		Poverty line (1.9$)		Poverty line (2.15$)	
	Shapley	Contribution (%)	Shapley	Contribution (%)	Shapley	Contribution (%)	Shapley	Contribution (%)
Physical capital	0.2317	0.82	0.1785	0.75	0.8378	2.38	0.6208	2.19
Financial capital	15.2451	53.65	13.0725	55.28	14.0999	40.11	12.1850	43.04
Social capital	9.8124	34.53	7.9678	33.7	11.3365	32.25	9.0171	31.85
Human capital	3.1234	10.99	2.4266	10.26	5.4489	15.5	4.2413	14.97

### Shapley decomposition of factors influencing vulnerability to health poverty among rural women of childbearing age

Considering the impact of the number of explanatory variables on the speed and computational efficiency of Shapley decomposition^42^, this study included statistically significant variables from Tobit regression analyses conducted in 2019 and 2022 for Shapley decomposition analysis. As shown in Table 5, regardless of the poverty line, the contribution rate of annual per capita household income was the highest (45.65%, 47.92%, 34.11%, 37.12%), followed by expenditures on social interactions (34.53%, 33.70%, 32.25%, 31.85%), respondents' age (4.25%, 4.03%, 12.11%, 11.69%), and household loans (7.12%, 6.62%, 3.43%, 3.36%), with other variables contributing less. Table 6 displays the contribution rates of the Shapley decomposition under the sustainable livelihood framework for vulnerability to health poverty. The results show that the contributing factors, from highest to lowest, were financial capital (53.65%, 55.28%, 40.11%, 43.04%), social capital (34.53%, 33.70%, 32.25%, 31.85%), human capital (10.99%, 10.26%, 15.50%, 14.97%), and physical capital (0.82%, 0.75%, 2.38%, 2.19%).

## Discussion

China has made significant progress in eradicating absolute poverty; however, the concept of poverty vulnerability as a precursor to future destitution offers a valuable perspective for further enhancing poverty reduction efforts^43^. Through an investigation focused on the vulnerability of rural married women of childbearing age in western China to health poverty, in both 2019 and 2022, there was an increase in vulnerability to health poverty, coinciding with an increase in the poverty line from the established standard of $1.90 to $2.15. In formulating future anti-poverty policies, it is crucial to employ health poverty indicators to identify populations at risk of falling into poverty due to illness. Moreover, it is essential to identify individuals who are prone to relapse into poverty as a result of health-related challenges. By implementing targeted preventive interventions, we can effectively address the underlying factors contributing to health poverty and work toward its eradication.

This study presents findings that demonstrate a noticeable decrease in both the prevalence of poverty and vulnerability to health poverty over a period of time, regardless of the specific poverty threshold used as a benchmark. The decline in both the prevalence of poverty and vulnerability to health poverty observed in this study can be attributed to the implementation of various poverty reduction strategies in China^44,45^. Therefore, efforts aimed at alleviating poverty not only reduce extreme poverty but also diminish the likelihood of experiencing poverty due to health-related issues in the future. Despite significant achievements in poverty eradication in China, it must be acknowledged that the possibility of rural women of childbearing age falling into poverty due to health issues has not been completely eliminated. Research results show that in 2022, 21.8% (below the poverty line of $1.90) and 28.2% (below the poverty line of $2.15) of rural women of childbearing age faced the risk of falling into poverty due to health issues. On the one hand, in rural areas, women of childbearing age often bear the responsibility of caring for their families and children, which makes it easy for them to neglect their own health needs^46^. On the other hand, due to the relatively underdeveloped medical facilities in rural areas of western China^47^, women of childbearing age may find it difficult to obtain timely and effective medical services when they encounter health problems, leading to a deterioration in their health condition and, consequently, greater vulnerability to health poverty.

Based on the sustainable livelihood framework, this study posits that vulnerability to health poverty should be comprehensively considered through multiple dimensions, including physical capital, financial capital, social capital, and human capital^48^. The unique advantage of the sustainable livelihood framework lies in its comprehensive set of indicators and its guiding role in empirical analysis. It can integrate multiple factors that may affect vulnerability to health poverty into a relatively mature and concise multidimensional analysis framework. Based on the sustainable livelihood theoretical framework, it is possible to accurately identify the risk factors affecting the vulnerability of people to health poverty with different characteristics and provide targeted suggestions from multiple perspectives.

The results of Shapley's decomposition show that within the dimensions of sustainable livelihood, financial capital has the greatest impact on vulnerability to health poverty, followed by social capital, human capital, and finally physical capital. Within financial capital, the per capita annual income of households contributes the most to reducing vulnerability to health poverty, which significantly decreases this vulnerability. This is consistent with the widespread understanding that higher income levels enable individuals to accumulate wealth and effectively address health-related risks^49^. Additionally, people with higher incomes who generally have better health literacy tend to pay more attention to their health^50,51^. Household loans also reduce vulnerability to health poverty, possibly because the capital accumulation obtained through borrowing can reduce the likelihood of rural women of childbearing age falling into health poverty.

Social capital significantly contributes to reducing vulnerability to health poverty, notably decreasing the vulnerability of rural women of childbearing age to health poverty. In rural areas, interpersonal networks are particularly important, with people helping and supporting each other to collectively cope with various challenges in life^52,53^. When rural women of childbearing age participate in activities such as celebrations of births and weddings, downries, New Year gifts, wedding gifts, and funeral expenses, they are able to establish and strengthen good social relationships with other villagers, thereby enhancing their social capital. This increase in social capital helps them receive more support and assistance when facing health issues.

In terms of human capital, the age of respondents contributes significantly to reducing vulnerability to health poverty, likely because as they age, rural women of childbearing age accumulate extensive life experience, including awareness of their own health status, knowledge of disease prevention and management, and strategies to cope with health risks, thus reducing the likelihood of falling into poverty due to health issues. The study also revealed that educational level plays a role in reducing the vulnerability of rural women of childbearing age to health poverty; women with higher educational levels typically have better health awareness^54,55^, which helps them manage their health more effectively and reduce medical expenses due to health issues.

Within physical capital, the type of drinking water contributes to vulnerability to health poverty. Compared to the use of tap water, the use of well water increases the vulnerability of rural women of childbearing age to health poverty, possibly because women who use tap water pay more attention to water quality and health issues, whereas families that rely on well water might lack the necessary knowledge or habits, leading to insufficient concern about water quality and thereby increasing health risks^56–58^. The separation of housing and kitchen also reduces the vulnerability of rural women of childbearing age to health poverty; separating the kitchen from the living space effectively isolates the fumes produced during cooking, significantly improving the indoor air quality and reducing the risk of respiratory diseases.

Despite the considerable progress made in eradicating absolute poverty in China, our analysis reveals that a particular subset of rural women of childbearing age continue to confront a significant risk of falling into poverty due to health-related issues. Consequently, it is imperative for future policy development in the realms of anti-poverty and welfare programs to prioritize the needs of these marginalized groups. Targeted interventions should be devised to address the issue of poverty, with a specific emphasis on alleviating the vulnerabilities faced by rural women during their childbearing years, particularly in relation to health-related impoverishment. These measures should be informed by a comprehensive understanding of the factors that contribute to such vulnerability.

### Strengths and limitations

This study has two strengths. First, by including data from 2019 and 2022, future poverty due to health issues among rural women of childbearing age can be predicted. Second, our research provides a considerable sample size based on population data, allowing for a comprehensive investigation of the factors influencing vulnerability to health poverty. However, our study also has limitations. One is the reliance on self-reported data for household income and expenditures, which may be subject to recall bias. Second, our analysis is limited to data collected during two specific periods—2019 and 2022—and does not fully explore the long-term patterns of dynamic changes in the vulnerability of rural women of childbearing age to health poverty.

## Conclusion

This study revealed that women of childbearing age in rural western China face the risk of falling into poverty due to health issues. The Shapley value decomposition results show that the four major contributors to the vulnerability of these women to health-related poverty are annual per capita household income, expenditures on social interactions, the age of the respondents, and household loans. To maintain poverty reduction achievements and prevent rural women of childbearing age from falling into poverty or returning to poverty due to illness, anti-poverty policies must prioritize these specific groups. A set of precise and efficient intervention policies should be formulated from a proactive perspective to consolidate the achievements of health poverty alleviation and prevent the return to poverty among rural women of childbearing age.

### Supplementary Information

Supplementary Information 1.

Supplementary Information 2.

Supplementary Information 3.

Supplementary Information 4.

Supplementary Tables.

## Data Availability

The data for this study are part of the overall project and are not publicly available. Access to the datasets of this study can be directed to the corresponding authors.

## References

[1] Jeremy M, Vincenzo F (2023). The role of social innovation in tackling global poverty and vulnerability. Front. Sociol..

[2] Qitao L (2022). Determinants of financial poverty alleviation efficiency: Evidence from Henan China. PLOS ONE.

[3] Chiara M, Dario S (2022). The dynamics of poverty in Europe: what has changed after the great recession?. J. Econ. Inequal.

[4] Qi Z, Xiaoqun H, Zhong L, Wanchun X, Liang Z (2019). The effects of poverty reduction policy on health services utilization among the rural poor: a quasi-experimental study in central and western rural China. Int. J. Equity Health.

[5] Muyi H (2023). Evaluation of typical ecosystem services in Dabie Mountain area and its application in improving residents' well-being. Front. Plant Sci..

[6] Yuanquan L, Li C, Yuan M (2023). How do public services supply, livelihood capital, and livelihood strategies affect subjective poverty?. PLOS ONE.

[7] Haiying L (2022). Measurement and identification of relative poverty level of pastoral areas: an analysis based on spatial layout. Environ. Sci. Pollut. Res. Int..

[8] Yusong L, Linyi Z (2022). Impact of the participation of rural households in the appraisal on poor households' identification. PLOS ONE.

[9] Jay P, Chu C, Yili Y (2021). Building a global community with shared future free from poverty via health poverty alleviation. Global Health J..

[10] Yongtian Z, Shigemitsu S, Rui G, Jin Y (2023). Poverty alleviation relocation, fuelwood consumption and gender differences in human capital improvement. Int. J. Environ. Res. Public Health.

[11] Fangkai Z, Jianjun J, Min Y, Kun Z, Dandi C (2023). Catastrophic health expenditure, incidence, trend and socioeconomic risk factors in China: A systematic review and meta-analysis. Front. Public Health.

[12] Peng W, Fanzhi W (2022). A study of the impact of land transfer decisions on household income in rural China. PLOS ONE.

[13] Bo S, Jiangli D, Gengli Z, Yu M, Linhong W (2020). The utilization of health examination by menopausal and older women - 6 provinces, China 2018. China CDC Week..

[14] Getaneh T, Derebe M, Light T (2023). Maternal psychological distress and associated factors among pregnant women attending antenatal care at public hospitals, Ethiopia. PLOS ONE.

[15] Peltzer K, Shikwane E, Matseke G (2011). Psychological distress and associated factors among a sample of pregnant women in South Africa. J. Psychol. Afr..

[16] Lilin Y (2021). Development of a novel nomogram for predicting premature rupture of membrane in pregnant women with *Vulvovaginal candidiasis*. Front. Med..

[17] Philip C, Guido E (2020). Defining and measuring health poverty. Soc. Sci. Med..

[18] Gaoling W, Xiaolin S, Zhaopeng C, Qianqian K, Shaoliang T (2022). The impact of informal social support on the health poverty vulnerability of the elderly in rural China: Based on 2018 CHARLS data. BMC Health Serv. Res..

[19] Qin X, Chaoyang Y, Ying M, Hui L, Jing W (2021). Classification and influencing factors of rural elderly's vulnerability to health-related poverty in central and western regions of China. Global Health J..

[20] Simões N, Crespo N, Moreira SB, Varum CA (2016). Measurement and determinants of health poverty and richness: evidence from Portugal. Empir. Econ..

[21] Costa GOT, Machado AF, Amaral PV (2018). Vulnerability to poverty in Brazilian municipalities in 2000 and 2010: A multidimensional approach. EconomiA.

[22] Junjun L, Gao-Ling W, Beilei Y (2019). A study of factors influencing the vulnerability of chronic disease patients to health poverty. China Health Econ..

[23] Yue L (2019). Exploring multidimensional risk factors for health poverty vulnerability in rural households. China Health Care Manag..

[24] Xiaoyan L (2024). Construction of a vulnerability risk indicator system for health poverty among rural elderly patients with chronic diseases. J. Nurs..

[25] Sowmya D (2016). Economic vulnerability to health shocks and coping strategies: evidence from Andhra Pradesh, India. Health Policy Plan..

[26] Mobeen M, Kabir KH, Schneider UA, Ahmed T, Scheffran J (2023). Sustainable livelihood capital and climate change adaptation in Pakistan's agriculture: Structural equation modeling analysis in the VIABLE framework. Heliyon.

[27] Kexin C (2024). Housing conditions, cooking fuels, and health-related quality of life among rural middle-aged and elderly in northwest China: A ten-year balanced panel study. Prevent. Med. Rep..

[28] Wenqin G (2022). The impact of healthcare reform on the dynamic changes in health service utilization and equity: a 10-year follow-up study. Sci. Rep. UK.

[29] Wang W, Chen K, Xiao W, Du J, Qiao H (2024). Determinants of health poverty vulnerability in rural areas of Western China in the post-poverty relief era: an analysis based on the Anderson behavioral model. BMC Public Health.

[30] Wenqin G (2022). The mediation path of physical multimorbidity on the vulnerability to health-related poverty of rural aging families in Ningxia, China: A cross-sectional survey. Front. Public Health.

[31] Yali L, Lei H (2022). Assessing the impact of public transfer payments on the vulnerability of rural households to healthcare poverty in China. BMC Health Serv. Res..

[32] Atake EH (2018). Health shocks in sub-Saharan Africa: Are the poor and uninsured households more vulnerable?. Health Econ. Rev..

[33] Thapa R, Dahl C, Aung WP, Bjertness E (2021). Urban-rural differences in overweight and obesity among 25–64 years old Myanmar residents: A cross-sectional, nationwide survey. BMJ Open.

[34] Éric H, Gaël G, Aurélie L, Christophe G (2023). Macroeconomic dynamics in a finite world based on thermodynamic potential. Sci. Rep. UK.

[35] Shorrocks AF (2013). Decomposition procedures for distributional analysis: A unified framework based on the Shapley value. J. Econ. Inequal..

[36] Jacob N, Justice N, Richard M, Chiwaula LS (2012). Health and vulnerability to poverty in Ghana: Evidence from the Ghana living standards survey round. Health Econ. Rev..

[37] Jie S, Yaping C, Yahong W, Salim K (2022). Health risk, income effect, and the stability of farmers’ poverty alleviation in deep poverty areas: A case study of S-County in Qinba Mountain Area. Int. J. Environ. Res. Public Health.

[38] Busse HA (2017). Participatory assessment of factors influencing nutrition and livelihoods in rural Ethiopia: Implications for measuring impacts of multisector nutrition programs. Food Nutr. Bull..

[39] Zhengyue J (2022). Identifying vulnerability to poverty and its determinants among older adults in empty-nest households: An empirical analysis from rural Shandong Province, China. Health Policy Plan.

[40] Jun H, Yining S, Wenhao T (2020). Social capital, risk resilience and poverty vulnerability of rural female-headed households-an empirical analysis based on CFPS data. J. Nanjing Agric. Univ..

[41] Yan W, Baomin T (2022). Health shocks, social capital, and rural household poverty vulnerability. Forum Stat. Inf..

[42] Maarit BS (2022). Preconception mental health, socioeconomic status, and pregnancy outcomes in primiparous women. Front. Public Health.

[43] Sharma M, Pradhan MR (2023). Socioeconomic inequality in cognitive impairment among India's older adults and its determinants: A decomposition analysis. BMC Geriatr..

[44] Wei Y, Zhang Z, Zhang M (2023). Effects of health poverty alleviation project from the perspective of vulnerability to poverty: Evidence from five Chinese prefectures. Glob. Health Action.

[45] Xiaoting C, Yanling Z, Haiquan C, Yu T (2023). Does opportunity co-creation help the poor entrepreneurs? Evidence from China. Front. Psychol..

[46] Ping QW, Hui WY, Ji ZW, Xia LC, Xu C (2018). Multidimensional poverty measurement of poverty-stricken counties in China's 14 conti-guous destitute areas considering ecological environment. Ying Yong Sheng Tai Xue Bao J. Appl. Ecol..

[47] Wenxin M (2023). Female perspective: the burden of Alzheimer's disease and other dementias in China from 1990 to 2019 and prediction of their prevalence up to 2044. Front. Public Health.

[48] Zhou J (2023). Analyzing the efficiency of Chinese primary healthcare institutions using the Malmquist-DEA approach: Evidence from urban and rural areas. Front. Public Health.

[49] Xin C, Jiajia L, Wenhao F, Feng F (2021). Association between livelihood capital and catastrophic health expenditure among patients with critical illness: A cross-sectional study in rural Shandong, China. BMJ Open.

[50] Dai Y (2016). Social support and the self-rated health of older people: A comparative study in Tainan Taiwan and Fuzhou Fujian province. Med. (Baltimore).

[51] Zhang K (2022). Global Burden of cardiomyopathy and myocarditis in the older adults from 1990 to 2019. Front. Public Health.

[52] Tianpei M (2021). Health literacy mediates the association between socioeconomic status and productive aging among elderly Chinese adults in a newly urbanized community. Front. Public Health.

[53] Novak SA, Webster GD (2011). Spousal social control during a weight loss attempt: A daily diary study. Pers. Relationship.

[54] Donfrancesco C (2008). Italian network for obesity and cardiovascular disease surveillance: A pilot project. BMC Family Pract.

[55] Wang J, Geng L (2019). Effects of socioeconomic status on physical and psychological health: Lifestyle as a mediator. Int. J. Environ. Res. Public Health.

[56] Xiaoyong H, Tiantian W, Duan H, Yanli W, Qiong L (2021). Impact of social class on health: The mediating role of health self-management. PLOS ONE.

[57] Rashedul IM, Bosong L, Yue Y, ChauChyun C, Mahdi M (2023). Comparative energetics of various membrane distillation configurations and guidelines for design and operation. Membranes.

[58] Baskin-Graves L (2019). Rapid health impact assessment of a proposed poultry processing plant in Millsboro, Delaware. Int. J. Environ. Res. Public Health.

[59] Lopes RH (2023). Worldwide surveillance actions and initiatives of drinking water quality: A scoping review. Int. J. Environ. Res. Public Health.

