# Supplementary material for: A study on the factors influencing the vulnerability of women of childbearing age to health poverty in rural western China
Source: Sci Rep. 2024 Jun 8;14:13219. doi: 10.1038/s41598-024-64070-z (PMC11162415; doi:10.1038/s41598-024-64070-z)
Supplement: Supplementary file 1 — Supplementary Information 1. [file 41598_2024_64070_MOESM1_ESM.pdf]

Note: pay attention to

1, set variable value 999 as missing (variable value 999 is missing unless otherwise specified in the questionnaire)

2、As there 're some data entry mistakes, check the variables first to see if there any extrem e s.

For the variables of expenditure and amount of services: replace th e 1% highest cases with missing.

(Before analysis, check whether each variable has specific values and logic errors. A maximum of 1% for continuous variables such as household income, expense and access was set as missing values.)

3、For Education Level (A7), the frequencies of categories (4)-(7) are very small.We 'd better merge categories (4)-(7) into category (4) which represents the senior high school and above.

(A7 education level: because the proportion of selection (4) - (7) is relatively small, so (4) - (7) can be combined into one classification (4) -high school and above)

4、Income: generate new variables

The household income=sum of the consumption expenditure=J13*12+J14+J15+J16(amount consumed by themselves*price)

I ncome per capita=the household income /number of people in the family

I ncome level: divide the income per capita into 5 groups, from the 20% lowest to the 20% highest

(Calculation of income: generating new variables

Household income = the sum of household consumption expenditure = J13 * 12 + J14 + J15 + J16 (the quantity of your own consumption * price) - - here, please select the specific variables according to the questionnaire

Household income per capita = household income / number of permanent household residents

Income level: divided into 5 groups by household per capita income:

Level 1:20% the lowest 20% of revenue,

Level 2: 20%-40%,

Level 3: 40%-60%,

Level4: 60%-80%,

Level 5:20% the highest Top 20% of revenue)

In the database, the household questionnaire has coded only the households, but not the surveyed members in the questionnaire.

The investigated members codes in the database have been supplemented

I. Outpatient (Outpatient Clinic)

a. % of being ill in the last two weeks (prevalence of past two weeks); among the ill:%seek care,%self tx,%no tx (among patients: visit rate, self-treatment rate, untreated rate)

1)V ariables:

B1 Do you feel physically unwell for the 14 days before the survey

B7 disease, whether treatment

B8 How did you treat it

2)% of being ill (prevalence): B1=1

%seek care among the ill (prevalence): B8=1 | B8=2 if B1=1

%self t x among the ill (disease self-treatment rate): B8=3 | B8=1 if B1=1

%no tx among the ill (disease-untreated rate): B7=2 if B1=1

Please note: the denominator calculated by visit rate, self-treatment rate and untreated rate are all the number of patients.

	Freq(number of people)	Percent (Number)%	The number of people	The person-time rate%
% of being ill	1973	17.1		
%seek care	767	38.9	1230	62.3
%self tx,	1085	55.0		
%no tx	493	25.0		

b. Among those with no tx:% distribution of reasons (untreated reason in untreated patients)

1) variables: B7.1 If not treated, what is the main reason

2)tab B7.1 if B1=1 & B7=2

	Freq	Percent
1 Self-feeling illness	124	25.2
2 Economic difficulties	283	57.4
3 No time	15	3.0
4 Transportation inconvenience	4	0.8
5. No effective measures	34	6.9
6 Other	33	6.7

c. Among those who seek care: (patient)

O nly analyze the first time.(Only the first visit)

i. Where?(To see a doctor)

1)Variables:

B12 Where was the first clinic visit?

Please ask the teachers of Ningxia Hospital to code the medical units: divide the medical institutions into: 1 village clinics, 2 private clinics, 3 township health hospitals, 4 county hospitals, 5 hospitals above the county level and 6 others

Clinic unit codes were supplemented in the database

2)tab B12 if B1=1 & B7=1 & B8!=3

	Freq	Percent
1 Village clinic	151	19.7
2 Private practice	83	10.8
3. Township health centers	190	24.8
4 County hospital	233	30.4
5 Hospitals above the county level	66	8.6
6 Other	44	5.7

ii. **THE, Saving account, reimbursement, OOP by where.(Total medical expenses, family account payment, reimbursement, self-payment)**

1)Variables:

B14 The total medical cost of this doctor

B14.1 How much is cash for the medical expenses

B14.2 How much yuan to pay in the family account

B14.3 How much yuan for reimbursement or exemption of the new rural cooperative medical care system

2)Total expenditure: B14 if B1=1 & B7=1 & B8!=3

Saving accout: B14.2 if B1=1 & B7=1 & B8!=3

Reimbursement: B14.3 if B1=1 & B7=1 & B8!=3

Out of pocket: B14.1 if B1=1 & B7=1 & B8!=3

Please do the logical checking if B14=B14.2+B14.3+B14.1 (Please do the logic check: whether B14=B14.2+B14.3+B14.1, list the proportion of yes or not

Except for the missing values, B14=B14.2+B14.3+B14.1

Total table:

Raw data calculation:

	T otal expenditure	Out of pocket	Saving account	Reimburs e-ment
Mean	542.9	523.5	12.6	30.9
Median	150.0	150.0	0.0	0.0
miss	22	26	90	88

The total amount of medical expenses, the cash out-of-pocket amount of medical expenses, the payment amount of the family account and the reimbursement or reduction amount of the new rural cooperative medical care system are mainly due to the following reasons. First, the respondent could not remember clearly. Second, farmers' understanding is limited to family accounts. Third, some farmers are not clear about the new rural cooperative medical service outpatient pooling sales reduction of the policy.

According to the medical institution: By where: By B12

		T otal expenditure	Out of pocket	Saving account	Reimb urse-ment
1 Village clinic	Mean	153.9	152.9	1.3	0.05
	Median	80.0	80.0	0.0	0.0
2 Private practice	Mean	248.3	251.4	0.05	0.6
	Median	150.0	160.0	0.0	0.0
3. Township health centers	Mean	257.7	268.8	6.2	5.1
	Median	100.0	100.0	0.0	0.0
4 County hospital	Mean	597.3	551.4	2.5	55.0
	Median	230.0	200.0	0.0	0.0
5 Hospitals above the county level	Mean	2362.6	2271.0	115.2	156.5
	Median	600.0	600.0	0.0	0.0
6 Other	Mean	782.5	711.6	0.0	1.2
	Median	200.0	200.0	0.0	0.0

d. **Among self tx (Self-Medical Person):**

i. **% distribution of reasons (Self-medical reasons)**

1) Variables: B9 Why did you choose self-medical treatment?

2)tab B9 if B1=1 & B7=1 & (B8=1|B8=3)

	Freq	Percent（%）
1 Self-treatment as prescribed by the physician	563	51.9
2 Self-reported illness is mild / no need to see a doctor	187	17.2
3. Self-medical treatment is cheaper	203	18.7
4 No time	11	1.0
5 inconvenient transportation	26	2.4
6 Poor service	2	0.2
7 Other	93	8.6

ii. Drug source

1) Variables: B10 If it is self-medical treatment, the source of your medicine

2) B10 if B1=1 & B7=1 & (B8=1|B8=3)

In the database, multiple choice is that each option is set as a variable. The variable name and the variable label may not indicate which topic it belongs to. Please be careful when making the analysis.

	Percent of cases
1 Existing at home	12.6%(137)
2 Buy it at the drugstore	53.7%(583)
3 bought by medical institutions (not seen)	43.1%(467)
4 People give	1.0%(11)
5 Other	1.1%(12)

iii. THE (total self-medical costs)

1) Variables: B10.1 If you buy drugs in a pharmacy or a medical institution, how much do you spend to buy drugs in these 14 days?

2)THE: B10.1 if B1=1 & B7=1 & (B8=1|B8=3) & (B10=2|B10=3)

	T otal expenditure
Mean	209.98
Median	70.00
miss	105 cases (respondents could not remember the data, numerical variables cannot be recorded with 999)

e. Top 5 health problems by level (Top five disorders)

1)Variables:

B3.2 What disease or injury-disease code are you suffering from (use the disease code table Please consult the disease code table to determine the name of the disease)

B12 Where was the first clinic visit?

2)tab B3.2 if B1=1

Ororder according to the first name of the disease

Order cis-position	Name of disease Disease name	Percent（%）
1health problem	070 (Acute nasopharyngitis)	1.9
2health problem	112 (Other sports disorders)	1.2
3health problem	080 (Acute and chronic gastroenteritis)	1.1
4health problem	086 (gallstones and cholecystitis)	1.0
5health problem	066 (hypertension)	0.8
6health problem	097 (Other female genital disorders)	0.8
	999 (signs, symptoms, and unknown condition)	1.9

B y level (Flow of visits to the top five diseases): by B12

if B1=1 & B7=1 & B8!=3

row percent（%）

Name of disease	Village Clinics	Private Clinics	Township Hospitals	County Hospitals	County Hospitals above	Others
1health problem	49.5	5.5	27．5	16.5	0	1.1
2health problem	22.2	16.7	36.1	22．2	0	2.8
3health problem	10	8.3	43.3	26.7	8.3	3.3
4health problem	10.9	9.1	23.6	47.3	3.6	5.5
5health problem	29.7	2.7	16.2	40.5	10.8	0

6health problem	8.2	18.4	16.3	42.9	2.0	12.2
999	15.7	13.7	13.7	47.1	5.9	3.9

f. % of being ill in the last two weeks; among the ill:%seek care,%self tx,%no tx by income (two-week prevalence, attendance rate, self-treatment rate, untreated rate in different income groups)

1)Variables:

B1 Do you feel physically unwell for the 14 days before the survey

B7 disease, whether it was treated

B8 How did you treat it

2)By income level:

% of being ill (prevalence): B1=1

%seek care among the ill (prevalence): B8=1 | B8=2 if B1=1 & B7=1

%self t x among the ill (disease self-treatment rate): B8=3 if B1=1 & B7=1

%no tx among the ill (disease-untreated rate): B7=2 if B1=1

When analyzing the table below, revenue set the maximum 1% to the missing value

Income	% of being ill	%seek care	%self tx	%no tx
Level 1	15.3	31.3	52.4	28.7
Level 2	16.1	33.0	50.5	32.7
Level 3	17.7	40.9	54.4	24.8
Level 4	19.3	43.6	55.8	20.1
Level 5	16.9	43.9	61.8	20.1

g. THE, saving, reimbursement, OOP by income level (total clinic expenses, family account expenses, reimbursement, self-pay for different income groups)

Present visit seeking care:

1)Variables:

B14 The total medical cost of this doctor

B14.1 How much is cash for the medical expenses

B14.2 How much yuan to pay in the family account

B14.3 How much yuan for reimbursement or exemption of the new rural cooperative medical care system

2)Total expenditure: B14 if B1=1 & B7=1 & B8!=3

Saving accout: B14.2 if B1=1 & B7=1 & B8!=3

Reimbursement: B14.3 if B1=1 & B7=1 & B8!=3

Out of pocket: B14.1 if B1=1 & B7=1 & B8!=3

When analyzing the table below, revenue set the maximum 1% to the missing value

Income		T otal expenditure	Out of pocket	Saving account	Reimburs ement
Level 1	Mean	425.4	390.0	4.6	40.9
	Median	80.0	80.0	0.0	0.0
Level 2	Mean	569.7	513.0	3.0	46.7
	Median	200.0	190.0	0.0	0.0
Level 3	Mean	455.3	431.6	4.1	3.3
	Median	180.0	150.0	0.0	0.0
Level 4	Mean	469.7	451.8	1.2	30.3
	Median	160.0	160.0	0.0	0.0
Level 5	Mean	764.4	787.3	43.2	40.1
	Median	200.0	200.0	0.0	0.0

Self-Medical Person self treatment:

THE: B10.1 if B1=1 & B7=1 & (B8=1|B8=3) & (B10=2|B10=3)

When analyzing the table below, revenue set the maximum 1% to the missing value

Income	Mean(first)	Median (Yuan)
Level 1	106.6	50.0
Level 2	181.7	60.0
Level 3	174.3	80.0
Level 4	301.9	100.0
Level 5	251.2	100.0

II. Inpatient (hospitalization)

a. % of needing hospital care in the last year; among the ill:%seek care,%no tx (hospitalization rate; among inpatients: hospitalization rate and hospitalization rate)

1)Variables:

C1 Has any doctor diagnosed your hospitalization in the past year?

C2 How many times in the past year has your doctor diagnosed you as needing a hospital and you did not?

C3 How many times have you been in a hospital in the past year?

2)% of needing hospital care: C1=1

% seek care: C3>0 if C1=1

% no tx: C2>0 if C1=1

	number of people	The number of people	Number rate (%)	The person-time rate is (%)
% of needing hospital care	867	1333	7.5	11.58
%seek care,	749	965	86.4	111.30
%no tx	118	368	13.6	42.45

b. Among those with no tx:% distribution of reasons (among hospitalized hospitalized, no hospitalized hospitalization)

1) Variables: C2.1 The main reason for no hospitalization

2) tab C2.1 if C1=1 & C2>0

The label in the questionnaire is wrong: (5) too high, (5) no beds, (6) other

Check whether the database has been changed over.

(This problem has been found when establishing the database, but the paper directly fills in the selected number, which cannot distinguish between 5 or 6, and the data is recorded according to the questionnaire)

	Freq	Percent
1 There is no need	8	4.1
2 No time	9	4.6
3 Economic difficulties	161	82.6
4 Poor service	1	.5
5 Too high price and no beds	6	3.1
6 Other	10	5.1

c. Among those who seek care: (Inpatient)

O nly analyze last hospital care.(Only the latest hospitalization)

i. Where?(Hospitality)

1)Variables:

C4.2 What is the name of the medical unit hospitalized this time?

Ask the teacher of Ningxia Medical College to code the name of the inpatient unit:

1 township hospitals 2 county hospitals 3 hospitals above the county level 4 others

The inpatient unit code has been added to the database

2) tab C4.2 if C1=1 & C3>0

	Freq	Percent
1. Township health centers	163	21.8
2 County hospital	399	53.3
3 Hospitals above the county level	146	19.5
4 Other	41	5.5

ii. THE, reimbursement, OOP by where.(The total medical expenses of different inpatient institutions, reimbursed by the new rural cooperative medical care system, self-payment)

1)Variables:

C4.8 In the past year, have you already participated in the new rural cooperative medical care system?

C4.8.1 If you participate in the new rural cooperative medical care system, what is the payment method?

C4.9 How much yuan did you pay for the hospitalization expenses?

C4.9.1 How much did the center reimburyou for inpatient medical expenses?

C4.10 How much yuan have you paid for this hospitalization expenses

C4.11 the travel expenses, nutrition expenses and escort expenses incurred to this hospitalization?

C4.12 Do you or several people give gifts or red envelopes to medical staff during your hospitalization?

C4.12.1 If any, how much is the total money?

2) If C1=1 & C3>0:

* Medical Expenditure: (Medical Expenditure)

If C4.8=1 & C4.8.1=1:

THE=C4.9

Reimbursement=C4.9.1

OOP=C4.9-C4.9.1

If C4.8=1 & C4.8.1=2:
OOP=C4.10
If C4.8=2:
THE=OOP=C4.10
* Other Expenditure (Other expenses)
C4.11 Travel expenses, nutrition and food expenses, and escort expenses
C4.12.1 gift or red envelope fee if C4.12! =4

Please check:
How many C4.9, C4.9.1, C4.10 are answered while C4.8 or C4.8.1 are missing?
And if C4.9, C4.9.1, C4.10 are all answered, does C4.9-C4.9.1=C4.10?
Please check:
How many C4.8 or C4.8.1 are missing, while C4.9, C4.9.1, C4.10 still answer?
No absence of C4.8 or C4.8.1
If C4.9, C4.9.1, and C4.10 are all answered, is it C4.9-C4.9.1=C4.10?
Follow this logical relationship to investigate and input the data

Total table:

If C4.8=1 & C4.8.1=1

	Medical Expenditure (Medical expenses)			Other Expenditure (Other expenditures)	
	T otal expenditure	Reimburse-ment	Out of pocket	C4.11 Vehicle Travel expenses, etc	C4.12 Gift fee, etc
Mean	3215.09	835.66	2382.60	645.08	211.43
Median	1863.41	260.00	1000.00	300.00	175.00

If C4.8=1 & C4.8.1=2 or If C4.8=2

	Medical Expenditure ---OOP (Medical expenses)	Other Expenditure (Other expenditures)	
		C4.11 Vehicle Travel expenses, etc	C4.12 Gift fee, etc
Mean	1955.53	736.18	902.50
Median	500.00	300.00	375.00

Group by different inpatient institutions:

If C4.8=1 & C4.8.1=1

		Medical Expenditure (Medical Expenditure)			Other Expenditure (Other expenditures)	
		T otal expenditure	Reimburse-ment	Out of pocket	C4.11 Vehicle Travel expenses , etc	C4.12 Gift fee, etc
1. Township health centers	Mean	1093.46	325.67	767.79	258.78	203.33
	Median	600.00	150.00	300.00	150.00	200.00
2 County hospital	Mean	2675.66	1044.09	1635.19	601.94	150.00
	Median	2000.00	500.00	1000.00	400.00	100.00
3 Hospitals above the county level	Mean	6674.60	974.93	5699.68	1042.07	500.00
	Median	3537.78	210.00	3000.00	500.00	500.00
4 Other	Mean	3954.73	121.21	3833.52	1105.48	0.00
	Median	2460.00	0.00	2100.00	3500.00	0.00

If C4.8=1 & C4.8.1=2 or If C4.8=2

	Medical Expenditure ---OOP (Medical Expenditure)	Other Expenditure (Other expenses)	
		C4.11 Vehicle Travel expenses, etc	C4.12 Gift fee, etc

1. Township health centers	Mean	440.23	176.96	0.00
	Median	300.00	65.00	0.00
2 County hospital	Mean	1533.02	608.00	375.00
	Median	1000.00	300.00	375.00
3 Hospitals above the county level	Mean	5577.79	2018.75	1430.00
	Median	3900.00	700.00	1430.00
4 Other	Mean	1290.00	1000.00	0.00
	Median	370.00	1000.00	0.00

d. Top 5 health problems by level (Top five disorders)

1)Variables:

C 4 The name of your illness hospitalized due to illness or injury poisoning?

(Please use the disease code table Please consult the disease code table to determine the disease name)

2)tab C4 if C1=1 & C3>0

Total table:

Order cis-position	Name of disease Disease name	Percent（%）
1health problem	Bolelithiasis and cholecystitis 086	8.3
2health problem	Appendiceal disease 082	6.4
3health problem	Normal delivery 102	6.3
4health problem	Hepatitis B, 012	3.6
5health problem	Acute and chronic gastroenteritis 080	3.6
6health problem	Other female genital diseases 097	3.5
7health problem	999	9.9

Hospital flow for the top five diseases:

C4， row percent

Name of disease	Township Hospitals	County Hospitals	County Hospitals above	Others
1health problem	19	35	6	2
(percent)	30.6%	56.5%	9.7%	3.2%
2health problem	12	32	3	1
(percent)	25.0%	66.7%	6.3%	2.1%
3health problem	13	31	3	0
(percent)	27.7%	66.0%	6.4%	.0%
4health problem	2	18	6	1
(percent)	7.4%	66.7%	22.2%	3.7%
5health problem	4	16	3	4
(percent)	14.8%	59.3%	11.1%	14.8%
6health problem	11	10	2	3
(percent)	42.3%	38.5%	7.7%	11.5%
7health problem	22	33	17	2
(percent)	29.7%	44.6%	23.0%	2.7%

e. % of needing hospital care in the last year; among the ill:%seek care,%no tx by income (hospitalization rate for different income groups; among inpatients: hospitalization rate, hospitalization rate)

1)Variables:

C1 Has any doctor diagnosed your hospitalization in the past year?

C2 How many times in the past year has your doctor diagnosed you as being hospitalized and you did not?

C3 How many times have you been in a hospital in the past year?

2)By income level:

% of needing hospital care: C1=1

% seek care: C3>0 if C1=1

% no tx: C2>0 if C1=1

When analyzing the table below, revenue set the maximum 1% to the missing value

Income	% of needing H ospital care	%seek care	%no tx
Level 1	127（14.8%）	116（15.7%）	23（11.9%）
Level 2	148（17.3%）	124（16.8%）	39（20.2%）

Level 3	181（21.1%）	148（20.0%）	51（26.4%）
Level 4	218（25.5%）	191（25.8%）	40（20.7%）
Level 5	182（21.3%）	160（21.7%）	40（20.7%）

f. THE, reimbursement, OOP by income level (medical expenditure, reimbursement, self-payment for different income groups)

1)Variables:

C4.8 In the past year, have you already participated in the new rural cooperative medical care system?

C4.8.1 If you participate in the new rural cooperative medical care system, what is the payment method?

C4.9 How much yuan did you pay for the hospitalization expenses?

C4.9.1 How much did the center reimburyou for inpatient medical expenses?

C4.10 How much yuan have you paid for this hospitalization expenses

C4.11 the travel expenses, nutrition expenses and escort expenses incurred to this hospitalization?

C4.12 Do you or several people give gifts or red envelopes to medical staff during your hospitalization?

C4.12.1 If any, how much is the total money?

Income Level

2) If C1=1 & C3>0:

* Medical Expenditure: (Medical Expenditure)

If C4.8=1 & C4.8.1=1:

THE=C4.9

Reimbursement=C4.9.1

OOP=C4.9-C4.9.1

If C4.8=1 & C4.8.1=2:

OOP=C4.10

If C4.8=2:

THE=OOP=C4.10

* Other Expenditure (Other expenses)

C4.11 Travel expenses, nutrition and food expenses, and escort expenses

C4.12.1 Gift or red envelope fee

If C4.8=1 & C4.8.1=1

When analyzing the table below, revenue set the maximum 1% to the missing value

		Medical Expenditure (Medical Expenditure)			Other Expenditure (Other expenditures)	
		T otal expenditure	Reimburse-ment	Out of pocket	C4.11 Vehicle Travel expenses , etc	C4.12 Gift fee, etc
Level 1	Mean	1996.09	458.27	1537.82	433.67	3.93
	Median	1060.00	150.00	700.00	200.00	4.00
Level 2	Mean	2185.65	785.95	1399.71	488.05	3.97
	Median	1300.00	300.00	700.00	225.00	4.00
Level 3	Mean	2751.03	1003.27	1747.76	517.63	3.91
	Median	2000.00	300.00	1000.00	300.00	4.00
Level 4	Mean	3139.57	811.57	2328.00	752.20	3.92
	Median	2000.00	300.00	1100.00	350.00	4.00
Level 5	Mean	4469.20	1013.63	3478.20	862.48	3.91
	Median	2000.00	300.05	1500.00	500.00	4.00

If C4.8=1 & C4.8.1=2 or If C4.8=2

When analyzing the table below, revenue set the maximum 1% to the missing value

		Medical Expenditure ---OOP (Medical Expenditure)	Other Expenditure (Other expenses)	
			C4.11 Vehicle Travel expenses, etc	C4.12 Gift fee, etc
Level 1	Mean	363.67	178.33	4.00
	Median	300.00	100.00	4.00
Level 2	Mean	1040.63	1829.23	4.00
	Median	275.00	300.00	4.00
Level 3	Mean	2428.57	581.54	4.00
	Median	1500.00	300.00	4.00
Level 4	Mean	1295.52	419.05	3.88
	Median	1000.00	400.00	4.00
Level 5	Mean	3634.12	1168.13	3.79
	Median	2500.00	650.00	4.00

III. Chronic (Chronic disease)

a. **% of having a chronic disease; among the ill:%seek care (prevalence of chronic disease, disease visits)**

1)Variables:

D1 Have you have a chronic disease diagnosed by a doctor in the past six months

D2 In the last three months, you have seen several times with these diseases

2)% of having a chronic disease: D1=1

%seek care:% of D2>0 if D1=1

	Freq	person-time	Percent（%）	person-time (%)
% of having a chronic disease	1528		13.3	
%seek care,	707	2001	46.3	130.96%

b. Among those who seek care: (patient)

i. Where?(To see a doctor)

1)Variables:

D3.1 In the last three months, have you referred to these diseases in the main institution?

2)tab D3.1 if D1=1 & D2>0

	Freq	Percent（%）
1 Village clinic	93	13.2
2. Township health centers	193	27.3
3 County hospital	314	44.4
4 Private practice	61	8.6
5 Other	46	6.5

ii. THE, Saving account, reimbursement, OOP by where.(Medical expenses of different medical institutions, family account payment, reimbursement, self-payment)

1)Variables:

D3.2 Whether you have participated in the new rural cooperative medical care system in the last three months

D3.2.1 If you participate in the new rural cooperative medical care system, the payment method is

D3.3 How much yuan have you paid for medical expenses

D3.3.1 How much yuan was paid by the family account for medical expenses?

D3.3.2 How much yuan is reimbursed by the medical expenses management center

D3.4 How much yuan do you pay yourself for the medical expenses yourself

2) If D1=1 & D2>0:

If D3.2=1 & D3.2.1=1:

THE=D3.3

Saving Account=D3.3.1

Reimbursement=D3.3.2

OOP=D3.3-D3.3.1-D3.3.2

If D3.2=1 & D3.2.1=2:

OOP=D3.4

If D3.2=2:

OOP=D3.4

Please check:

How many D3.3, D3.3.1, D3.3.2,D3.4 are answered while D3.2 or D3.2.1 are missing?

And if D3.3, D3.3.1, D3.3.2,D3.4 are all answered, does D3.3-D3.3.1-D3.3.2=D3.4?

Please check:

How many D3.2 or D3.2.1 were missing, while D3.3, D3.3.1, D3.3.2, D3.4 were still answered?

D3.2 is not missing, D3.2.1 is missing reason: ⑴ The respondent did not know the chronic disease pooling policy implemented in January 2009, and could not answer this option. ⑵ patients were all self-paid, ⑶ patients paid with the remaining money from the previous family account, and the patient did not know.

(Note: The answer to the questionnaire is the same as D3.3 and D3.3.1 data, which means that the patient directly uses the family account to see the doctor. If the survey is conducted according to the table, D3.2.1 should answer 2 and directly jump to D3.4, then D3.4 is directly 0, which does not reflect the cost of the patient.）

If D3.3, D3.3.1, D3.3.2, and D3.3.4 are all answered, is it D3.3-D3.3.1-D3.3.2=D3.4?

Our survey is at the end of February, the sea is in January 2009 chronic diseases as a whole, farmers basically don't know chronic diseases can be reimbursed, in the survey, students think D3.3= D3.4, entry personnel directly put four entry, now do check, call up the original questionnaire, clarify the logical relationship after the data cleaning, we will this part of the problem according to D3.3=D3.3.1+D3.3.2, entry! If you answer D3.4, then the first three vacancies!

Total table:

IfD 3.2 & D3.2.1=1 (missing values for D 3.2.1 part included in the analysis)

	T otal expenditure	Saving account	Reimburse-ment	Out of pocket
Mean	961.6319	191.9472	94.4120	961.6319
Median	400.0000	.0000	.0000	400.0000

If D3.2=1 & D3.2.1=2 or If D3.2=2 (missing values for part D 3.2.1 included in analysis)

	Out of pocket
Mean	856.3171
Median	300.0000

Group by different medical institutions: By where: D3.2

If D3.2 & D3.2.1=1

		T otal expenditure	Saving account	Reimburse-ment	Out of pocket
1 Village clinic	Mean	441.0395	52.9730	15.7895	
	Median	300.0000	.0000	.0000	
2 Township health centers	Mean	501.1875	56.3057	42.8428	
	Median	200.0000	.0000	.0000	
3 County hospital	Mean	1310.7462	345.0583	146.1989	
	Median	600.0000	.0000	.0000	
4 Private practice	Mean	808.9815	69.0741	44.0556	
	Median	300.0000	.0000	.0000	
5 Other	Mean	1676.5854	156.8049	176.2650	
	Median	450.0000	.0000	.0000	

If D3.2=1 & D3.2.1=2 or If D3.2=2

		Out of pocket
1 Village clinic	Mean	724.2857
	Median	350.0000
2 Township health centers	Mean	450.8056
	Median	260.0000
3 County hospital	Mean	1091.6897
	Median	360.0000
4 Private practice	Mean	617.0000
	Median	100.0000
5 Other	Mean	1894.0000
	Median	300.0000

D3.5 Cost of self-purchased drugs in pharmacies.

If D1=1

	Buy drugs by yourself
Mean	449.7786
Median	120.0000

c. Top 5 health problems by level

1) Variables: D1.1 If any, what are the diseases?

(Please use the disease code table Please consult the disease code table to determine the disease name)

2) tab D1.1 if D1=1

(Order by the first most severe chronic disease)

Order cis-position	Name of disease Disease name	fre	Percent
1health problem	Hypertension 066	227	2.0
2health problem	Acute and chronic gastroenteritis 080	138	1.2
3health problem	Gallstones and cholecystitis 086	123	1.1
4health problem	Hepatitis B, 012	107	0.9
5health problem	Rheumatoid arthritis 109	83	0.7

d. % of having a chronic disease in the last half year ; among the ill: %seek care by income

1)Variables:

D1 Have you have a chronic disease diagnosed by a doctor in the past six months

D2 In the last three months, you have seen them several times with these diseases

2)By income:

% of having a chronic disease: D1=1

%seek care:% of D2>0 if D1=1

When analyzing the following table, the maximum 1% of income was set to the missing value

Income Level	number of people	% of having chronic disease	number of people	% of seeking care
Level 1	298	12.9%	103	34.6%
Level 2	284	12.4%	118	41.5%
Level 3	299	13.0%	134	44.8%
Level 4	369	16.1%	197	53.4%
Level 5	266	12.2%	147	55.3%

e. THE, saving, reimbursement, OOP,income by income level

1)Variables:

D3.2 Whether you have participated in the new rural cooperative medical care system in the last three months

D3.2.1 If you participate in the new rural cooperative medical care system, the payment method is

D3.3 How much yuan have you paid for medical expenses

D3.3.1 How much yuan was paid by the family account for medical expenses?

D3.3.2 How much yuan is reimbursed by the medical expenses management center

D3.4 How much yuan do you pay yourself for the medical expenses yourself

2) If D1=1 & D2>0:

If D3.2=1 & D3.2.1=1:

THE=D3.3

Saving Account=D3.3.1

Reimbursement=D3.3.2

OOP=D3.3-D3.3.1-D3.3.2

If D3.2=1 & D3.2.1=2:

OOP=D3.4

If D3.2=2:

OOP=D3.4

Group group by per capita income level: by income level

When analyzing the following table, the maximum 1% of income was set to the missing value

If D3.2 & D3.2.1=1

		T otal expenditure	Saving account	Reimburse-ment	Out of pocket
Level 1	Mean	469.0741	51.6138	80.5432	
	Median	300.0000	.0000	.0000	
Level 2	Mean	663.4455	168.4495	116.4771	
	Median	300.0000	.0000	.0000	
Level 3	Mean	738.2328	201.7391	17.9478	
	Median	300.0000	.0000	.0000	
Level 4	Mean	1098.9636	124.4063	69.4878	
	Median	460.0000	.0000	.0000	
Level 5	Mean	1612.99	396.50	189.930	
	Median	500.0000	.0000	.0000	

If D3.2=1 & D3.2.1=2 or If D3.2=2

When analyzing the following table, the maximum 1% of income was set to the missing value

Income		Out of pocket
Level 1	Mean	530.4091
	Median	310.0000
Level 2	Mean	458.0769
	Median	300.0000
Level 3	Mean	583.9000
	Median	280.0000
Level 4	Mean	669.0909
	Median	230.0000

Level 5	Mean	1538.91
	Median	415.000

D3.5 Cost of independent drug purchase at pharmacies By income level
If D1=1

When analyzing the following table, the maximum 1% of income was set to the missing value

Income		Buy drugs by yourself
Level 1	Mean	242.25
	Median	60.0
Level 2	Mean	310.9576
	Median	120.0000
Level 3	Mean	485.3358
	Median	200.0000
Level 4	Mean	588.6751
	Median	150.0000
Level 5	Mean	497.38
	Median	100.00

IV. **Catastrophic expenditure: (Risk health expenditure)**

a. Catastrophic expenditure= OOP (OP+IP+chronic+self tx)/income

1) Variables:

J14.4 Household expenses-drugs, medical expenses, etc. (including only the amount of medical expenses paid by oneself)

2) Being pressed for time, we will use J14.4 as OOP at this stage.(J14.4 as out-of-pocket medical expenses)

OOP=J14.4

Income=the household income

Catastrophic expenditure=J14.4/the household income

	Catastrophic expenditure
Mean	.12004
Median	.05085

b. Catastrophic expenditure by income level :

Income	Mean	Median
Level 1	0.1079	0.0554
Level 2	0.1124	0.0632
Level 3	0.1245	0.0662
Level 4	0.1362	0.0838
Level 5	0.1213	0.0353

V. Poverty

We will send the instructions later.

VI. Prevention

a. MCH [separately for those born last year

F: Married women of childbearing age aged 15-49 years: A3 sex; A5 age; A6 marital status

If A3=2 & A5>=15 & A5<=49 & (A6=2|A6=3|A6=4|A6=5)

F1 Have you ever had a gynecological examination in the past year?

	Freq	Percent（%）
1 Is	564	36.2
2 No	993	63.8

F2 Did you deliver after January 1,2003?

	Freq	Percent（%）
1 Is	577	37.0
2 No	981	63.0

Could you please ask the teacher of Ningxia Medical College to explain: in which year did the new rural cooperative medical care policy of fixed compensation for delivery costs begin to be implemented? Is all the women can get subsidies, or only the women who deliver in the township hospital can get? Is subsidy to join a tube center to get, still deduct directly from childbirth cost?

Consulted Haiyuan County, Haiyuan from January 1,2007 to implement the new rural cooperative medical care, the same year also began to implement the compensation for the cost of childbirth. The implementation plan stipulates that all inpatient institutions can subsidize, the subsidy is directly reduced from the delivery costs, (the hospital outside the county is their own advance payment). However, Haiyuan County is a reduction project and a Swedish maternal and child health project county, and there is compensation

for hospital delivery, so the cost of childbirth in township health centers is basically free, but many respondents only know that their delivery is not free, nothing else)

The data in the following table are calculated based on the year of the compensation for the delivery fee in Haiyuan County.

Year of delivery	Freq	Percent（%）
The new rural cooperative medical care for the delivery of labor costs before the fixed compensation	430	44.1
After the new rural cooperative medical care for the cost of childbirth quota compensation	544	55.9

F3 is the most recent delivery, how many prenatal tests?

If F2=1

	The number of antenatal examinations
Mean	2.16
Median	2
% of F3=0	183（31.9%）

F3.1 Was the first prenatal visit done at what week of pregnancy?

If F2=1 & F3>0

	Time of the first antenatal visit (week)
Mean	18
Median	12

F3.2 Where did you have a prenatal examination?

If F2=1 & F3>0

	Percent（%）
1 county / district and above hospitals	26.3
2 county / district and above traditional Chinese medicine hospitals	23.5
3 Maternal and child health care institutions	7.7
4. Township and street health centers	43.5
5 Community health service center	0
6. Family planning guidance station	0.8
7 Health clinic / place / station	3.1
8 Other	1.3

F3.3 Prenatal inspection items?

If F2=1 & F3>0

	Freq	Percent（%）
1 Measuring weight	113	28.9
2 Blood test	101	25.8
3 Measuring blood pressure	163	41.7
4. Routine urine examination	133	34
5 B ultrasound inspection	369	94.4

F4 Place of delivery?

If F2=1

	Freq	Percent（%）
1 county level and above hospitals	99	17.2
2 county and above TCM hospitals	41	7.1
3 Maternal and child health care institutions	35	6.1
4 Township health centers	125	21.8
5 Community health service center	0	0
6. Family planning guidance station	0	0
7 Health clinic / place / station	5	0.9

8 In the home	260	45.3
9 Other	9	1.6

This form cannot be filled out, the mother and the child's code is different, the questionnaire does not investigate the specific birth date of the last delivery!

	The delivery time is before the new rural cooperative medical system implementation quota compensation	Delivery time in the implementation of the new rural cooperative quota compensation
1 county level and above hospitals		
2 county and above TCM hospitals		
3 Maternal and child health care institutions		
4 Township health centers		
5 Community health service center		
6. Family planning guidance station		
7 Health clinic / place / station		
8 In the home		
9 Other		

F4.1 The main reason for not visiting the hospital if childbirth at home

If F2=1 & F4=8

Income	1 There is no need	%	2 Too late	%	3 Economic difficulties	%	4 Transportation inconvenience	%	5 Other	%
Level 1	13	18.1	22	30.6	24	33.3	12	16.7	1	1.4
Level 2	17	26.6	21	32.8	15	23.4	8	12.5	3	4.7
Level 3	10	20.8	19	39.6	10	20.8	6	12.5	3	6.3
Level 4	8	20.6	8	20.5	18	46.2	5	12.8	0	0
Level 5	9	25.0	14	38.9	8	22.2	2	5.6	3	8.3
Total	57	22.0	84	32.4	75	29.0	33	12.7	10	3.9

F4.2 If delivery at home, who is the midwife?

If F2=1 & F4=8

	Freq	Percent（%）
1 Township and above doctors	4	1.5
2 Village doctor	28	10.8
3. Full-time birth attendant	51	19.6
4. Non-full-time midwives	60	23.1
5 Family pick up	106	40.8
6 Other	11	4.2

F5-F5.3 Cost of delivery

Please ask the teacher of Ningxia Medical College: F5-F5.3, in the medical expenditure part of hospitalization and chronic disease, when the payment method is selected 1, the jump is set after answering the advance payment and reimbursement of expenses, so that there is no need to answer the question of self-paid expenses. However, in the delivery cost, F5.2 has no jump set. How did we do in the investigation, is the jump survey, or no jump, the three questions are investigated?

There is no jump in this part of the questionnaire, but according to the requirements of the training manual, c4.8.1----c4.10 was calculated. All three questions were investigated.

If F2=1:

If F5=1:

THE=F5.1

Reimbursement=F5.2

OOP=F5.1-F5.2

If F5=2:

OOP=F5.3

Please check:
How many F5.1, F5.2,F5.3 are answered while F5 is missing?
And if F5.1,F5.2 ,F5.3 are all answered, does F5.1-F5.2=F5.3?

Total table:

If F2=1 & F5=1

	T otal expenditure	Reimbursement	Out of pocket
Mean	766.89	155.03	611.86
Median	400	0	250

If F2=1 & F5=2

	Out of pocket
Mean	267.25
Median	50

Cost for different places of delivery: By where: F4
If F2=1 & F5=1

		T otal expenditure	Reimbursement	Out of pocket
1 county / district and above hospitals	Mean	1454	224.81	1229.19
	Median	530	98.5	500
2 county / district or above	Mean	910.29	369.14	541.14
	Median	540	150	350
3 Maternal and child health care institutions	Mean	702.12	149.12	553
	Median	500	125	350
4. Township and street health centers	Mean	428.52	94.03	334.49
	Median	300	0	175
5 Community health service center	Mean	0	0	0
	Median	0	0	0
6. Family planning guidance station	Mean	0	0	0
	Median	0	0	0
7 Health clinic / place / station	Mean	260.67	96.33	164.33
	Median	300	0	120
8 In the home	Mean	213.53	22.35	191.18
	Median	100	0	100
9 Other	Mean	280	0	280
	Median	200	0	200

If F2=1 & F5=2

		Out of pocket
1 county / district and above hospitals	Mean	1144.29
	Median	200
2 county / district or above	Mean	400
	Median	400
3 Maternal and child health care institutions	Mean	0
	Median	0
4. Township and street health centers	Mean	93.53
	Median	0
5 Community health service center	Mean	0
	Median	0
6. Family planning guidance station	Mean	0
	Median	0
7 Health clinic / place / station	Mean	208
	Median	208

8 In the home	Mean	101.05
	Median	0
9 Other	Mean	300
	Median	300

Delivery costs at different income levels: By income level

If F2=1 & F5=1

The table below does not set the maximum value of 1% as a missing value (less valid data)

Income		T otal expenditure	Reimbursement	Out of pocket
Level 1	Mean	662.67	189.18	473.49
	Median	300	50	200
Level 2	Mean	600.86	111.95	488.91
	Median	300	0	177.5
Level 3	Mean	835.41	192.03	643.38
	Median	500	0	250
Level 4	Mean	777.93	147.67	630.26
	Median	460	0	350
Level 5	Mean	995.86	132.07	863.79
	Median	465	0	300

If F2=1 & F5=2

The table below does not set the maximum value of 1% as a missing value (less valid data)

		Out of pocket
Level 1	Mean	196.25
	Median	10
Level 2	Mean	388.75
	Median	75
Level 3	Mean	81.67
	Median	25
Level 3	Mean	467.29
	Median	75
Level 4	Mean	83.33
	Median	50

F6 Number of times you have received postpartum visits within 42 days after delivery

F2=1

	Average number of postpartum visits	Number of postpartum visits by non-home producers （If F4≠8）
Mean	0.36	0.52
Median	0	0
% of F3=0	481（84.1%）	243（77.4%）

b. Children

Investigate children under 5 years: IfA 5 (age) <5

Group by age: by A5

The missing values of this part, ⑴ is due to the insider is absent or remembering, unable to investigate,

G2 nearly 12 months, the child received several health examination?

	G2 The number of health physical examinations
Mean	0.58
Median	0
% of G2=0	596（68.1%）
miss	108

Do G3 children have a planned immunization card or manual?

	Freq	Percent
1 Have	724	83.1
2 No	127	14.6
3 Don't know	20	2.3
miss	103	

Did the G4 accept the BCG vaccine?

	Freq	Percent
1 Is	725	83.1
2 No	62	7.6
miss	160	

How many times did G4.1 receive the PdT vaccine?

	G4.1 Number of DPT vaccination
Mean	2.56
Median	3
% of G4.1=0	105（13.5%）
miss	203

G4.2 How many times did polio sugar pills?

	G4.2 Number of times of taking polio sugar pills
Mean	2.72
Median	3
% of G4.2=0	82（10.3%）
miss	185

Is G4.3 vaccinated against measles?

	Freq	Percent（%）
1 Is	606	75.1
2 No	201	24.9
miss	167	

G4.4 How many times was hepatitis B vaccinated?

	G4.4 Number of hepatitis B vaccine administered
Mean	2.57
Median	3
% of G4.2=0	46（5.6%）
miss	166

G4.4.1 Time of the first dose of hepatitis B vaccine?

If G4.4>0

	Freq	Percent
1 within 24 hours after birth	544	66
2 within 1 week after birth	39	4.7
3 within 1 month after birth	66	8
Over 4 1 months	97	11.8
5 Not clear	78	9.5
miss	150	

Where is the G5 usually immunized?

	Freq	Percent
1 CDC	11	1.3
2 Health center	151	18
3 Community health service center	17	2
4 Health clinic / place / station	585	69.6
5 Other	76	9
miss	134	

Does the G6 child have diarrhea (diarrhea) in the past 2 weeks?

	Freq	Percent
1 Is	123	14.5
2 No	728	85.5
miss	123	

Have you ever eaten G7 during diarrhea?

If G6=1

	Freq	Percent
1 Oral rehydration solution with salt and powder	83	85.6
2 Oral rehydration solution and salt solution	9	9.3
3 Home-made oral	5	5.2

rehydration solution and salt solution	
miss	26

VII. TB knowledge (Knowledge knowledge of tuberculosis)

For TB and H ypertension, only keep the cases who answered the questions (like household). In this part, we only need to analyze the record of answering the question. The survey members were coded on the questionnaire, but this variable is not available in the database. Can you ask the teachers of Ningxia Medical College to check the following questionnaire and increase this variable. Otherwise we could not correspond to other variables such as the education of the respondents.

This variable has been added to the database, and the tables with different educational levels have been analyzed
Since it is not possible to match the education level for the time being, only the aggregate and the cross table with the income level are analyzed.

a. By education: (by educational level)
Education: A7 education level: The proportion of high school education or above is very small, which can be combined into one classification.
b. By income: Income Level (by income level)

c. By education: (by educational level)
Education: A7 education level: The proportion of high school education or above is very small, which can be combined into one classification.
d. By income: Income Level (by income level)

I2.1 Do you think that tuberculosis is a serious disease?

Column percent

	1 Didn't go to school<br>Education 1	2 Primary school<br>Education 2	3 Junior high school<br>Education 3	4 High school or above<br>Education 4	amount to<br>Total
1 Very serious	42.9%	54.2%	55.7%	67.8%	50.8%
2 Some serious	14.4%	11.7%	21.1%	13.6%	14.8%
3 Not very serious	3.7%	3.4%	4.1%	5.1%	3.7%
4 Don't know	38.9%	30.6%	19.1%	13.6%	30.7%

The maximum income value of 1% was set as missing value (24 households were excluded during the analysis, and 2484 households were analyzed)

	Income 1	Income 2	Income 3	Income 4	Income 5
1 Very serious	47.9%	46.5%	53.0%	53.9%	52.9%
2 Some serious	13.1%	17.2%	14.3%	12.7%	17.2%
3 Not very serious	2.5%	3.7%	3.5%	5.0%	4.6%
4 Don't know	36.5%	32.5%	29.2%	28.4%	25.3%

I2.2 In what way did you know about tuberculosis?

By Education:

	1 Didn't go to school<br>Education 1	2 Primary school<br>Education 2	3 Junior high school<br>Education 3	4 High school or above<br>Education 4	amount to<br>Total
1 Newspapers and periodicals	2.1%	2.3%	4.4%	8.5%	3.0%
2 broadcasting	2.6%	2.4%	4.3%	6.8%	3.1%
3 TV	16.8%	21.0%	27.3%	36.4%	21.4%
4 Billboard	3.6%	2.9%	7.0%	7.6%	4.2%
5 Publicity manual, etc	8.5%	11.4%	22.4%	24.6%	13.2%
6 Medical staff	16.3%	15.5%	18.4%	26.3%	16.9%
7 Family and friends, etc	40.7%	41.6%	48.2%	40.7%	42.5%
8 Religious leaders	0.7%	0.4%	.0%	.0%	0%.4
9 The teacher	1.2%	.3%	1.5%	.8%	1.0%
10 Other	1.1%	2.0%	1.7%	.8%	1.6%
11 Don't know	37.5%	30.9%	20.1%	11.0%	30.3%

By income :

The maximum income value of 1% was set as missing value (24 households were excluded during the analysis, and 2484 households were analyzed)

	Income 1	Income 2	Income 3	Income 4	Income 5
1 Newspapers and periodicals	0.8%	2.4%	3.3%	4.1%	4.6%
2 broadcasting	2.4%	3.7%	1.7%	2.8%	4.8%
3 TV	16.8%	20.5%	21.5%	25.2%	24.6%
4 Billboard	2.5%	4.7%	3.3%	5.6%	5.5%
5 Publicity manual, etc	8.6%	12.2%	14.3%	14.9%	17.0%
6 Medical staff	17.0%	17.0%	16.8%	16.4%	17.7%
7 Family and friends, etc	40.0%	39.3%	45.8%	41.8%	46.2%
8 Religious leaders	0.7%	0.6%	0.2%	0.4%	0.2%
9 The teacher	0.0%	0.8%	0.6%	1.9%	1.8%
10 Other	.8%	2.0%	1.0%	2.2%	2.1%
11 Don't know	34.1%	32.7%	28.2%	28.4%	27.1%

I2.3 What symptoms does pulmonary tuberculosis have?

By Education:

	1 Didn't go to school<br>Education 1	2 Primary school<br>Education 2	3 Junior high school<br>Education 3	4 High school or above<br>Education 4	amount to<br>Total
1 Rash	0.8%	1.2%	0.6%	5.1%	1.1%
2 Cough	28.3%	30.4%	42.0%	54.2%	33.1%
3 Cough for more than 3 weeks	9.2%	11.1%	14.7%	25.4%	11.8%
4 Cough blood	10.4%	10.0%	11.2%	25.4%	11.1%
5 Severe headache	3.8%	3.2%	3.3%	9.3%	3.7%
6 Nausea	4.4%	5.9%	6.4%	9.3%	5.6%
7 Thin	7.9%	5.9%	7.9%	18.6%	7.7%
8 Fever	6.2%	7.0%	7.2%	18.6%	7.3%
9 Unknown cause of fever for more than 7 days	1.1%	1.5%	.8%	3.4%	1.3%
10 Chest pain	9.5%	10.4%	10.3%	16.9%	10.3%
11 Short gas	17.3%	18.1%	20.5%	27.1%	18.7%
12 and was associated with persistent weakness	9.1%	9.4%	13.0%	16.1%	10.3%
13 Other	0.6%	0.9%	0.6%	0.0%	0.7%
14 Don't know	61.3%	58.0%	46.4%	28.8%	55.5%

By income:

The maximum income value of 1% was set as missing value (24 households were excluded during the analysis, and 2484 households were analyzed)

	Income 1	Income 2	Income 3	Income 4	Income 5
1 Rash	1.0%	0.6%	1.2%	1.3%	1.6%
2 Cough	31.8%	31.0%	31.3%	35.3%	36.6%
3 Cough for more than 3 weeks	7.9%	10.8%	9.9%	14.4%	16.3%
4 Cough blood	9.2%	9.7%	11.0%	12.1%	14.3%
5 Severe headache	1.8%	3.7%	3.3%	4.5%	5.7%
6 Nausea	3.0%	4.3%	5.0%	6.9%	9.2%
7 Thin	4.0%	4.9%	9.1%	8.2%	13.3%
8 Fever	4.2%	5.3%	6.8%	9.9%	10.6%
9 Unknown cause of fever for more than 7 days	.8%	1.2%	.4%	1.5%	2.5%
10 Chest pain	6.1%	9.1%	8.9%	11.6%	17.5%
11 Short gas	15.3%	18.5%	16.6%	21.1%	23.0%
12 and was associated with persistent weakness	7.4%	8.1%	9.5%	11.2%	16.3%
13 Other	.2%	1.2%	.4%	.6%	1.1%

14 Don't know	60.3%	57.4%	56.3%	51.9%	49.9%

I2.4 through what way can infect tuberculosis?

By education:

	1 Didn't go to school Education 1	2 Primary school Education 2	3 Junior high school Education 3	4 High school or above Education 4	amount to Total
1 Shake hands	1.9%	1.9%	3.5%	6.8%	2.4%
2 Droplets from a TB person who coughs or sneezes	19.4%	25.0%	34.0%	50.8%	25.9%
3. Eat together	15.2%	17.5%	27.3%	30.5%	19.2%
4 Public tableware	9.9%	12.5%	20.7%	28.0%	13.9%
5 Touch public items	3.1%	2.1%	3.1%	5.1%	2.8%
6 Other	1.7%	1.7%	1.5%	3.4%	1.7%
7 Don't know	73.5%	68.4%	55.1%	37.3%	66.1%

By income:

The maximum income value of 1% was set as missing value (24 households were excluded during the analysis, and 2484 households were analyzed)

	Income 1	Income 2	Income 3	Income 4	Income 5
1 Shake hands	1.7%	2.0%	1.9%	3.4%	3.0%
2 Droplets from a TB person who coughs or sneezes	20.0%	22.5%	24.2%	32.8%	32.2%
3. Eat together	13.8%	16.8%	19.3%	24.1%	23.9%
4 Public tableware	9.7%	11.8%	13.0%	15.5%	21.1%
5 Touch public items	1.7%	1.4%	1.9%	5.0%	4.8%
6 Other	0.5%	2.2%	1.7%	2.4%	2.3%
7 Don't know	71.8%	69.8%	68.7%	60.8%	57.5%

I2.5 How to prevent pulmonary tuberculosis?

By Education:

	1 Didn't go to school Education 1	2 Primary school Education 2	3 Junior high school Education 3	4 High school or above Education 4	amount to Total
1 Avoid shaking hands	2.5%	2.9%	5.6%	7.6%	3.5%
2 Cover your mouth and nose when coughing or sneezing	15.6%	18.4%	23.6%	39.8%	19.4%
3. Avoid eating with others	15.0%	19.2%	26.5%	33.1%	19.7%
4 Wash your hands after touching the items in a public place	4.9%	6.2%	8.7%	18.6%	6.8%
5. Close the window	2.1%	1.0%	1.7%	2.5%	1.6%
6. Good nutrition	3.3%	3.5%	4.1%	6.8%	3.7%
7 Pray	1.8%	1.0%	1.5%	.8%	1.4%
8 Don't know	75.5%	71.7%	59.2%	50.8%	69.6%
9 Other	1.1%	1.3%	2.7%	1.7%	1.6%

By income :

The maximum income value of 1% was set as missing value (24 households were excluded during the analysis, and 2484 households were analyzed)

	Income 1	Income 2	Income 3	Income 4	Income 5
1 Avoid shaking hands	2.2%	3.0%	2.5%	5.4%	4.6%
2 Cover your mouth and nose when coughing or sneezing	12.6%	19.3%	17.2%	24.6%	24.6%
3. Avoid eating with others	13.1%	16.8%	20.5%	24.6%	25.7%
4 Wash your hands after	4.4%	4.9%	6.0%	9.3%	10.1%

touching the items in a public place					
5. Close the window	0.8%	0.8%	1.0%	3.2%	2.5%
6. Good nutrition	1.8%	3.0%	2.5%	5.8%	6.2%
7 Pray	1.2%	1.4%	1.0%	2.2%	1.4%
8 Don't know	77.3%	73.0%	71.0%	63.1%	61.4%
9 Other	1.5%	.8%	1.4%	1.5%	2.8%

I2.6 Who do you think is more susceptible to tuberculosis?

By Education:

	1 Didn't go to school<br>Education 1	2 Primary school<br>Education 2	3 Junior high school<br>Education 3	4 High school or above<br>Education 4	amount to<br>Total
1 Anyone	32.8%	29.2%	33.7%	39.8%	32.0%
2 Only the poor	3.5%	3.9%	3.9%	5.9%	3.8%
3 Only the homeless	2.0%	2.0%	3.1%	2.5%	2.2%
4 Only the alcoholics	3.5%	4.6%	7.9%	5.1%	4.9%
5 Only for the drug users	5.0%	6.4%	8.7%	6.8%	6.3%
6 Only those living with HIV	2.3%	2.5%	2.5%	5.9%	2.6%
7 Only jtime	0.8%	0.8%	0.2%	0.8%	0.7%
8 Other	46.5%	45.4%	38.7%	35.6%	44.0%

By income :

The maximum income value of 1% was set as missing value (24 households were excluded during the analysis, and 2484 households were analyzed)

	Income 1	Income 2	Income 3	Income 4	Income 5
1 Anyone	29.2%	32.0%	29.8%	32.8%	36.8%
2 Only the poor	3.5%	4.5%	2.7%	3.4%	4.6%
3 Only the homeless	2.0%	2.2%	0.6%	3.4%	2.8%
4 Only the alcoholics	4.0%	5.5%	4.8%	6.0%	4.4%
5 Only for the drug users	3.2%	7.5%	6.8%	9.9%	4.6%
6 Only those living with HIV	0.8%	2.6%	1.7%	4.7%	3.7%
7 Only jtime	0.3%	1.2%	0.2%	1.3%	0.5%
8 Other	47.2%	43.2%	45.5%	43.1%	41.1%

I2.7 Can tuberculosis be cured?

By Education:

	1 Didn't go to school<br>Education 1	2 Primary school<br>Education 2	3 Junior high school<br>Education 3	4 High school or above<br>Education 4	amount to<br>Total
1 Can	59.6%	59.2%	69.6%	79.7%	62.5%
2 Can't	31.0%	30.3%	24.6%	16.9%	28.7%
999 (not known)	9.4%	10.5%	5.8%	3.4%	8.8%

By income :

The maximum income value of 1% was set as missing value (24 households were excluded during the analysis, and 2484 households were analyzed)

	Income 1	Income 2	Income 3	Income 4	Income 5
1 Can	61.5%	60.9%	59.0%	64.4%	67.6%
2 Can't	32.1%	31.0%	31.1%	25.4%	22.3%
999 (not known)	6.4%	8.1%	9.9%	10.1%	10.1%

I2.8 How should the pulmonary tuberculosis patient be treated?

By Education:

	1 Didn't go to school<br>Education 1	2 Primary school<br>Education 2	3 Junior high school<br>Education 3	4 High school or above<br>Education 4	amount to<br>Total
1 Chinese herbal medicine	10.6%	9.7%	8.9%	14.4%	10.1%
2 Rest at home without treatment	2.9%	1.8%	1.4%	0.8%	2.1%
3 Prayer	1.8%	1.5%	0.6%	0.8%	1.4%

4 Specific drugs given by the medical facilities	36.3%	38.7%	47.8%	65.3%	40.9%
5DOTS policy	3.1%	3.4%	4.1%	5.1%	3.5%
6 Don't know	52.6%	51.3%	43.3%	26.3%	49.0%
7 Other	2.1%	2.2%	2.9%	4.2%	2.4%

By income :

The maximum income value of 1% was set as missing value (24 households were excluded during the analysis, and 2484 households were analyzed)

	Income 1	Income 2	Income 3	Income 4	Income 5
1 Chinese herbal medicine	7.4%	8.3%	8.5%	12.7%	14.5%
2 Rest at home without treatment	2.2%	2.6%	1.7%	2.2%	1.6%
3 Prayer	1.5%	1.8%	1.0%	1.3%	1.4%
4 Specific drugs given by the medical facilities	38.0%	35.1%	41.6%	42.7%	47.6%
5DOTS policy	1.3%	2.6%	4.3%	6.0%	3.9%
6 Don't know	53.4%	53.8%	49.3%	44.4%	42.8%
7 Other	2.9%	2.4%	1.7%	3.0%	1.8%

I2.9 If you think you have tuberculosis, will you get it?

By Education:

	1 Didn't go to school<br>Education 1	2 Primary school<br>Education 2	3 Junior high school<br>Education 3	4 High school or above<br>Education 4	amount to<br>Total
1 Will	93.6%	95.4%	98.1%	99.2%	95.4%
2 Will not	6.4%	4.6%	1.9%	.8%	4.%6

By income :

The maximum income value of 1% was set as missing value (24 households were excluded during the analysis, and 2484 households were analyzed)

	Income 1	Income 2	Income 3	Income 4	Income 5
1 Will	93.1%	96.1%	95.7%	96.8%	95.9%
2 Will not	6.9%	3.9%	4.3%	3.2%	4.1%

I2.9.1 If treatment, where would you choose to go?

If I2.9=1

By Education:

	1 Didn't go to school<br>Education 1	2 Primary school<br>Education 2	3 Junior high school<br>Education 3	4 High school or above<br>Education 4	amount to<br>Total
1 Go to the village clinic	4.3%	3.8%	2.4%	4.3%	3.7%
2. Go to the township health centers	12.7%	11.0%	7.9%	10.3%	10.9%
3. Go to hospitals above the county level	75.0%	76.1%	77.5%	70.1%	75.7%
4 Go to the tuberculosis center	16.3%	17.6%	22.5%	30.8%	18.8%
5 Go to the pharmacy to buy your medicine	1.9%	1.7%	2.2%	.9%	1.8%
6 Traditional therapy (such as traditional Chinese medicine)	1.2%	1.5%	1.6%	.9%	1.4%
7 Self-treatment (e. g., herbal medicine)	0.6%	0.8%	0.6%	0.0%	0%.6
8 Other	1.8%	1.5%	1.8%	1.7%	1.7%

By income :

The maximum income value of 1% was set as missing value (24 households were excluded during the analysis, and 2484 households were analyzed)

	Income 1	Income 2	Income 3	Income 4	Income 5

1 Go to the village clinic	3.4%	3.5%	4.8%	2.9%	4.1%
2. Go to the township health centers	9.9%	11.5%	13.0%	10.5%	9.4%
3. Go to hospitals above the county level	76.7%	78.4%	74.7%	74.2%	74.3%
4 Go to the tuberculosis center	17.3%	16.2%	16.7%	21.8%	22.5%
5 Go to the pharmacy to buy your medicine	1.4%	1.6%	1.3%	2.2%	2.9%
6 Traditional therapy (such as traditional Chinese medicine)	1.3%	.2%	1.9%	1.6%	1.9%
7 Self-treatment (e. g., herbal medicine)	0.5%	0.4%	0.6%	0.7%	0.7%
8 Other	1.6%	1.6%	1.9%	1.6%	1.7%

I2.9.2 What is the reason for you not going to a medical institution?

If I2.9=2

By Education:

	1 Didn't go to school<br>Education 1	2 Primary school<br>Education 2	3 Junior high school<br>Education 3	4 High school or above<br>Education 4	amount to<br>Total
1 Don't know where to see a doctor	3.2%	4.8%	0%	0%	3.5%
2 The cost is too high	53.2%	52.4%	40.0%	100.0%	52.2%
3. Inconvenient transportation / too far away	16.1%	19.0%	50.0%	.0%	20.0%
4. Do not trust the medical staff	1.6%	2.4%	10.0%	.0%	2.6%
5 Poor attitude of medical staff	0%	0%	0%	0%	0%
6 Work can not do without	0%	0%	0%	0%	0%
7 Don't want to know the bad news	0%	2.4%	.0%	0%	0%.9
8 Other	24.2%	9.5%	10.0%	0%	17.4%

By income :

The maximum income value of 1% was set as missing value (24 households were excluded during the analysis, and 2484 households were analyzed)

	Income 1	Income 2	Income 3	Income 4	Income 5
1 Don't know where to see a doctor	0%	5.0%	0%	13.3%	5.6%
2 The cost is too high	61.0%	60.0%	52.4%	40.0%	33.3%
3. Inconvenient transportation / too far away	14.6%	10.0%	19.0%	40.0%	27.8%
4. Do not trust the medical staff	0%	0%	4.8%	6.7%	5.6%
5 Poor attitude of medical staff	0%	0%	0%	0%	0%
6 Work can not do without	0%	0%	0%	0%	0%
7 Don't want to know the bad news	2.4%	.0%	.0%	.0%	.0%
8 Other	22.0%	15.0%	9.5%	13.3%	22.2%

I2.10 What do you think of the cost of tuberculosis diagnosis and treatment?

By Education:

	1 Didn't go to school<br>Education 1	2 Primary school<br>Education 2	3 Junior high school<br>Education 3	4 High school or above<br>Education 4	amount to<br>Total
1 Should be free	29.9%	33.6%	41.4%	45.8%	34.4%
2 The cost is reasonable	3.2%	6.4%	8.9%	14.4%	6.1%
3 The cost is a little too high	9.1%	8.6%	8.5%	4.2%	8.6%
4 The cost is very high	10.9%	10.1%	8.3%	9.3%	10.0%
5 Don't know	46.9%	41.3%	32.9%	26.3%	41.0%

By income :

The maximum income value of 1% was set as missing value (24 households were excluded during the analysis, and 2484 households were analyzed)

	Income 1	Income 2	Income 3	Income 4	Income 5
1 Should be free	30.3%	33.3%	33.7%	40.5%	35.2%
2 The cost is reasonable	5.2%	5.7%	6.0%	4.7%	9.2%
3 The cost is a little too high	9.7%	7.9%	8.5%	8.0%	8.3%
4 The cost is very high	10.6%	8.9%	9.5%	11.4%	9.4%
5 Don't know	44.2%	44.2%	42.2%	35.3%	37.9%

I2.11 What ways do you want to obtain the knowledge and information on tuberculosis prevention?

By Education:

	1 Didn't go to school<br>Education 1	2 Primary school<br>Education 2	3 Junior high school<br>Education 3	4 High school or above<br>Education 4	amount to<br>Total
1 Newspapers and periodicals	3.1%	3.8%	6.0%	6.8%	4.15
2 broadcasting	5.9%	6.4%	8.3%	11.0%	6.8%
3 TV	39.5%	42.4%	45.1%	44.9%	41.9%
4 Billboard	4.7%	4.4%	8.3%	6.8%	5.5%
5 Publicity manual, etc	23.9%	23.7%	34.6%	36.4%	26.6%
6 Medical staff	47.0%	40.4%	41.2%	45.8%	43.3%
7 Family and friends, etc	33.2%	28.9%	28.6%	23.7%	30.3%
8 Religious leaders	1.4%	1.1%	.6%	.0%	1.1%
9 The teacher	2.4%	1.9%	1.9%	4.2%	2.2%
10 Other	8.4%	6.2%	3.7%	4.2%	6.4%

By income :

The maximum income value of 1% was set as missing value (24 households were excluded during the analysis, and 2484 households were analyzed)

	Income 1	Income 2	Income 3	Income 4	Income 5
1 Newspapers and periodicals	2.5%	4.1%	4.3%	3.9%	5.7%
2 broadcasting	6.6%	5.9%	5.6%	7.3%	8.5%
3 TV	36.5%	42.8%	45.5%	46.3%	39.8%
4 Billboard	4.4%	5.1%	5.8%	5.2%	7.1%
5 Publicity manual, etc	17.8%	24.1%	28.0%	34.1%	32.4%
6 Medical staff	43.7%	41.0%	41.0%	42.7%	48.5%
7 Family and friends, etc	29.2%	33.1%	29.8%	31.0%	26.9%
8 Religious leaders	1.5%	.8%	1.4%	.6%	.9%
9 The teacher	1.2%	.8%	1.7%	2.8%	5.1%
10 Other	8.2%	6.9%	5.8%	5.2%	5.5%

VIII. Hypertension knowledge (Knowledge knowledge of hypertension)

a. By education
b. By income

I3.1 Is the onset of hypertension related to diet?

Column percent

	Education 1	Education 2	Education 3	Education 4	Total
1 Have	33.3%	36.8%	50.7%	67.8%	39.8%
2 No	12.5%	14.8%	12.6%	8.5%	13.2%
3 Don't know	54.2%	48.3%	36.8%	23.7%	47.0%

The maximum income value of 1% was set as missing value (24 households were excluded during the analysis, and 2484 households were analyzed)

Income	Income 1	Income 2	Income 3	Income 4	Income 5
1 Have	30.6%	36.9%	39.1%	48.1%	46.7%
2 No	12.6%	12.6%	14.9%	13.1%	12.9%
3 Don't know	56.8%	50.5%	46.0%	38.8%	40.5%

I3.1.1 Does how much salt do you eat to affect the incidence of hypertension?

	Education 1	Education 2	Education 3	Education 4	Total

1 Have	34.0%	36.7%	51.3%	61.0%	39.8%
2 No	8.7%	11.1%	8.1%	5.1%	9.3%
3 Don't know	57.4%	52.2%	40.6%	33.9%	51.0%

The maximum income value of 1% was set as missing value (24 households were excluded during the analysis, and 2484 households were analyzed)

Income	Income 1	Income 2	Income 3	Income 4	Income 5
1 Have	32.3%	34.1%	41.0%	47.2%	46.4%
2 No	7.6%	11.4%	11.0%	8.0%	8.7%
3 Don't know	60.2%	54.4%	48.0%	44.8%	44.8%

I3.2 Is hypertension incidence related to cigarette smoking?

	Education 1	Education 2	Education 3	Education 4	Total
1 Have	33.7%	38.3%	53.2%	69.5%	41.1%
2 No	7.0%	7.4%	7.2%	4.2%	7.1%
3 Don't know	59.2%	54.3%	39.7%	26.3%	51.9%

The maximum income value of 1% was set as missing value (24 households were excluded during the analysis, and 2484 households were analyzed)

Income	Income 1	Income 2	Income 3	Income 4	Income 5
1 Have	33.9%	39.3%	40.4%	47.0%	46.7%
2 No	5.4%	6.3%	7.9%	9.7%	6.7%
3 Don't know	60.7%	54.4%	51.8%	43.3%	46.7%

I3.3 Is hypertension disease related to obesity?

	Education 1	Education 2	Education 3	Education 4	Total
1 Have	39.7%	46.1%	59.4%	75.4%	47.8%
2 No	6.7%	7.1%	6.2%	3.4%	6.6%
3 Don't know	53.6%	46.8%	34.4%	21.2%	45.7%

The maximum income value of 1% was set as missing value (24 households were excluded during the analysis, and 2484 households were analyzed)

Income	Income 1	Income 2	Income 3	Income 4	Income 5
1 Have	36.5%	44.2%	47.4%	57.3%	57.0%
2 No	7.1%	6.3%	8.7%	3.7%	6.9%
3 Don't know	56.5%	49.5%	43.9%	39.0%	36.1%

I3.4 Is hypertension disease related to alcohol consumption?

	Education 1	Education 2	Education 3	Education 4	Total
1 Have	37.3%	39.8%	58.2%	72.9%	44.2%
2 No	3.8%	5.1%	4.4%	1.7%	4.3%
3 Don't know	58.9%	55.1%	37.3%	25.4%	51.5%

The maximum income value of 1% was set as missing value (24 households were excluded during the analysis, and 2484 households were analyzed)

Income	Income 1	Income 2	Income 3	Income 4	Income 5
1 Have	35.5%	41.8%	43.1%	53.4%	49.9%
2 No	4.0%	4.7%	5.2%	3.4%	3.9%
3 Don't know	60.5%	53.5%	51.8%	43.1%	46.2%

I3.5 What diseases can a hypertensive patient develop if his blood pressure is not effectively controlled?

	Education 1	Education 2	Education 3	Education 4	Total
1 Stroke	20.4%	23.3%	32.3%	44.9%	25.1%
2 CHD	9.5%	12.2%	20.3%	33.9%	13.8%
3 Tumor	3.6%	4.5%	4.8%	6.8%	4.3%
4 Will not develop	0.5%	0.8%	0.8%	0%	0%.6
99 Don't know	74.3%	70.9%	58.2%	44.9%	68.4%

The maximum income value of 1% was set as missing value (24 households were excluded during the analysis, and 2484 households were analyzed)

Income	Income 1	Income 2	Income 3	Income 4	Income 5
1 Stroke	17.5%	21.1%	25.5%	30.8%	32.4%
2 CHD	7.9%	9.9%	13.9%	17.2%	21.8%
3 Tumor	2.5%	3.0%	3.9%	5.2%	7.1%
4 Will not develop	0.3%	0.4%	0.6%	0.4%	1.1%
99 Don't know	78.7%	73.8%	67.7%	60.3%	57.9%

I3.6 How should a hypertensive patient control his blood pressure?

	Education 1	Education 2	Education 3	Education 4	Total
Take medicine according to your doctor's advice	27.3%	30.2%	41.4%	48.3%	32.3%
2 According to the doctor's advice conditioning diet control salt	19.1%	23.0%	29.8%	44.9%	23.9%
3 Eat less meat and eggs with fat and cholesterol	16.8%	18.4%	26.7%	39.0%	20.5%
4. Keep yourself emotionally stable	9.3%	12.8%	12.4%	22.9%	11.8%
5 Do appropriate exercise	6.7%	10.0%	11.6%	25.4%	9.8%
6 Control weight	7.4%	8.4%	9.9%	21.2%	8.9%
7 No control	0.7%	0.7%	0.6%	0.8%	0%.7
99 Don't know	67.3%	63.9%	50.1%	34.7%	61.0%

The maximum income value of 1% was set as missing value (24 households were excluded during the analysis, and 2484 households were analyzed)

Income	Income 1	Income 2	Income 3	Income 4	Income 5
Take medicine according to your doctor's advice	27.2%	28.2%	32.7%	37.1%	37.5%
2 According to the doctor's advice conditioning diet control salt	15.5%	20.3%	23.2%	30.2%	32.6%
3 Eat less meat and eggs with fat and cholesterol	12.6%	17.0%	19.5%	27.4%	28.0%
4. Keep yourself emotionally stable	6.9%	9.7%	10.1%	14.7%	19.1%
5 Do appropriate exercise	4.4%	5.9%	8.7%	13.8%	17.7%
6 Control weight	3.9%	6.7%	7.5%	11.6%	16.3%
7 No control	0.3%	0.8%	0.2%	0.2%	1.6%
99 Don't know	69.6%	67.9%	60.2%	53.4%	50.6%

IX. Smoking (Smoking)

a. By education

b. By income

I1.1 Do you smoke any cigarettes?

In the smoking part, we unified that the family members over 15 years old must answer, but the family members can also answer the question if they know! I1.2, I1.3 and I1.4 have some missing values, insiders can not remember clearly did not answer this question!

	Education 1	Education 2	Education 3	Education 4	Total
1 Never suck	1836	1640	881	240	4597
	39.9%	35.7%	19.2%	5.2%	100.0%
2 Occasionally suck	37	53	35	12	137
	27.0%	38.7%	25.5%	8.8%	100.0%
3 Often suck	127	231	226	55	639
	19.9%	36.2%	35.4%	8.6%	100.0%
4 Has quit smoking	15	23	11	4	53
	28.3%	43.4%	20.8%	7.5%	100.0%

Income	Income 1	Income 2	Income 3	Income 4	Income 5
1 Never suck	951	883	904	926	933
	20.7%	19.2%	19.7%	20.1%	20.3%
2 Occasionally suck	34	27	31	23	22
	24.8%	19.7%	22.6%	16.8%	16.1%
3 Often suck	118	125	128	147	121
	18.5%	19.6%	20.0%	23.0%	18.9%
4 Has quit smoking	7	12	13	9	12
	13.2%	22.6%	24.5%	17.0%	22.6%

I1.2 How old did you start smoking?

If I1.1=2|I1.1=3

Education	Mean Age	Median Age
1 Didn't go to school	21.96	20.00
2 Primary school	20.67	20.00
3 Junior high school	21.23	20.00
4 High school and above	22.51	22.00
Total	86.37	82

Income	Mean Age	Median Age
Level 1	21.33	20.00
Level 2	20.97	20.00
Level 3	21.47	20.00
Level 4	21.34	20.00
Level 5	21.39	20.00

I1.3 How many cigarettes do you smoke per day?

If I1.1=2|I1.1=3

Education	Mean	Median
1 Didn't go to school	13.18	10.00
2 Primary school	14.13	15.00
3 Junior high school	17.56	20.00
4 High school and above	14.43	12.50
Total	59.3	57.5

Income	Mean	Median
Level 1	11.90	10.00
Level 2	13.43	15.00
Level 3	15.10	20.00
Level 4	16.93	20.00
Level 5	18.05	20.00

I1.4 How much do you cost to smoke every month?

If I1.1=2|I1.1=3

Education	Mean	Median
1 Didn't go to school	71.805	40.000
2 Primary school	75.167	60.000
3 Junior high school	93.196	65.000
4 High school and above	105.227	75.000
Total	345.395	240

Income	Mean	Median
Level 1	48.583	35.000
Level 2	74.196	60.000
Level 3	79.974	60.000
Level 4	90.693	60.000
Level 5	114.714	90.000

I1.5 How many years have you quit smoking?
If I1.1=4

Education	Mean	Median
1 Didn't go to school	3.67	2.00
2 Primary school	6.96	3.00
3 Junior high school	3.45	1.00
4 High school and above	6.25	4.50
Total	20.33	10.5

Income	Mean	Median
Level 1	6.57	1.00
Level 2	4.58	2.50
Level 3	5.31	3.00
Level 4	2.78	1.00
Level 5	19.24	7.5

I1.5.1 The main reasons for quitting smoking?
If I1.1=4

	Education 1	Education 2	Education 3	Education 4	Total
1 Has been sick	10	13	6	2	31
	32.3%	41.9%	19.4%	6.5%	100.0%
2. Prevention of disease	1	4	1	1	7
	14.3%	57.1%	14.3%	14.3%	100.0%
3 Economic reasons	6	2	3	0	11
	54.5%	18.2%	27.3%	.0%	100.0%
4 Family opposition	3	3	4	0	10
	30.0%	30.0%	40.0%	.0%	100.0%
5 Environmental restrictions	0	0	0	0	0
	0	0	0	0	0
6 Set up the image	0	0	0	0	0
	0	0	0	0	0
7 After publicity and education	0	0	0	0	0
	0	0	0	0	0
8 On the doctor's advice	1	4	0	0	5
	20.0%	80.0%	.0%	.0%	100.0%
9 Other	3	3	1	1	8
	37.5%	37.5%	12.5%	12.5%	100.0%
10 Don't know	0	1	0	0	1
	.0%	100.0%	.0%	.0%	100.0%

Income	Income 1	Income 2	Income 3	Income 4	Income 5
1 Has been sick	5	9	4	8	5
	16.1%	29.0%	12.9%	25.8%	16.1%
2. Prevention of disease	2	2	1	1	1
	28.6%	28.6%	14.3%	14.3%	14.3%
3 Economic reasons	2	1	5	2	1
	18.2%	9.1%	45.5%	18.2%	9.1%
4 Family opposition	2	1	5	1	1
	20.0%	10.0%	50.0%	10.0%	10.0%
5 Environmental restrictions	0	0	0	0	1
	.0%	.0%	.0%	.0%	100.0%
6 Set up the image	7	12	13	9	12
	13.2%	22.6%	24.5%	17.0%	22.6%
7 After publicity and education	7	12	13	9	12
	13.2%	22.6%	24.5%	17.0%	22.6%
8 On the doctor's advice	1	2	1	0	1
	20.0%	40.0%	20.0%	.0%	20.0%
9 Other	1	1	3	0	3
	12.5%	12.5%	37.5%	.0%	37.5%
10 Don't know	0	0	0	0	1
	.0%	.0%	.0%	.0%	100.0%

attach:

Disease classification- -coding table codes disease name encode disease name

A . Infectious disease meter
001. Typhoid fever and pay typhoid fever
002. Bacterial food poisoning 003. Dysrhea
004., Hepatitis A
005. Other intestinal infectious diseases 006. tuberculosis 007. tetanus 008, sepsis

.
009 Measles.
010. Epidemic Japanese encephalitis
. 011 Epidemic hemorrhagic fever
012., hepatitis B
013 Lepptospirosis
014. Atypical pneumonia
015. Other parenteral infectious diseases
B. Parasitic disease meter
016. Malaria
017., and schistosomiasis
018. Other parasitic diseases
C. Malignant tumor meter
019. Nasopharyngeal malignant tumors

021 esophageal malignancy 022 gastric colon malignancy 023 rectal and anal malignancy 024 liver malignancy 025 pancreatic malignancy 026 trachea. Bronchial and lung malignancies
027. Malignancies of the breast
028. Malignancies of the cervix
029. Leukemia
030. Other malignancies
D. Benign, in situ, and dynamic undetermined tumor plan
. 031 Benign uterine tumors
032. Benign brain tumors
. 033 Other benign tumors
034., and an orthotopic tumor
035. Tumor meter with undetermined dynamics or unknown dynamics

E.incretion. Nutritional and metabolic diseases and immune diseases
036. hyperthyroidism
037. Diabetes Mellitus
038. Poor nutrition or poor nutrition
. 039 Internal: rickets
. 040 Obesity and other hypernutrition
041. Other internal, camp, generation, and immune diseases
F. Subtotal of disorders of the blood and hematopoietic organs
042. The anemia
043. Other blood and hematopoietic organ diseases
G. Psychiatry
044. Old age, organic psychosis in the early stage of old age
045. Schizophrenia
046. Depression
047. Other mental disorders
H. Neurological disease
048. Meningitis
049. Vitilampsia
. 050 Acute infective polyneuritis
. 051 In Parkinson's disease
052. Other neurological disorders
I. Eye and apillary disease meter
053. Glaucoma
054. Cataracts
055. Corneal diseases meter
056. Other ocular and accessory diseases
J. Ear and mastoid disorders meter
057. otitis media and mastoiditis
058. Other ear and mastoid diseases
K. Subtotal of circulatory system diseases
059. Acute rheumatic fever
060. Chronic rheumatic heart disease
061. Angina pectoris
062. Acute myocardial infarction
063. Other ischemic heart diseases
064. Pulmonary primary heart disease

065. Other types of heart disease
066., Patients with hypertension
067., and cerebrovascular disease
068. Varices of the lower limbs
069. Other circulatory system diseases

L. Subtotal of respiratory diseases
070. Acute nasopharyngeal (common cold)
. 071 Acute pharynx, larynx, tonsils and trachea, etc

Upper call road infection 072. influenza 073. Pneumonia

. 074 Chronic pharynx and laryngitis 075 emphysema

. 076 Other chronic obstructive pulmonary disease (COPD, including
Slow branch, etc.)
077. The asthma
. 078 Other respiratory diseases (including acute lower breathing
A tract infection)
M. Subtotal of digestive system diseases
079. Teeth disease
Other oral or salivary gland and jaw disorders
080. Acute and chronic gastroenteritis
081. peptic ulcer
082. Disease of appendix
083.
Abdominal hernia 084.
Intestinal obstruction 085. Chronic liver disease and liver cirrhosis
086. Bolelithiasis and cholecystitis
087. Other digestive system disorders
N. Subtotal of urogenital diseases
088. nephritis and kidney disease
089. pyelitis
090. Urinary stones
091. Other urological disorders
092. Prostatic hyperplasia or inflammation
093. Other male genital diseases
094. mammary gland disease

095. Tupingitis and ovariitis
096. Uterovaginal prolapse
097. Other female genital diseases
O. Subtotal incidence of pregnancy, labor disease and puerperium disease
098. natural abortion
099. * An induced abortion
100. Bleeding in pregnancy and delivery

101. Pregnancy and hypertension syndrome
102. * Normal delivery
103. obstructed labor
104. Paratalytic disease
105. Other pregnancy and delivery diseases and complications of the puerperium
P. Subtotal of skin and subcutaneous tissue disease
106. Carbuncle and boils
107. Dermatitis
108. Other skin and subcutaneous tissue disorders
Q.muscle. Subtotal of the skeletal system and connective tissue disorders
109. Rheumatoid arthritis
110. Disc disease
111 and osteomyelitis
112
R. Innate anomalies
113. Congenital heart disease
114. Other congenital abnormalities
S. Subtotal of cases of perinatal origin
115 Premature and immature infants
116
117. Fetal and neonatal asphyxia
118. Neonatal tetanus
119. Other neonatal diseases
T. Subtotal of injury and poisoning
120
121. The dislocation. Sprains and strain
122. Intracranial and in vivo injuries (including nerves)
123. Open trauma and vascular injury
124
125. Poisoning and toxic effects

126. Other injuries and poisoning
**V.* Other small plan**
127., and pregnancy monitoring
128. Stertering
129. Hospitalization for special treatment
130. Individual and population examinations
131., for other reasons
999. Signs, symptoms, and ambiguity

* Not for the disease.

Note: ⑴ When filling in the disease code, fill in the three Arabic digits corresponding to the disease name (such as 012 for hepatitis B); ⑵ In the disease classification, it is impossible to list all the disease names, fill in the code of "other · · disease" in the kind of disease that the disease belongs to. For example, for ovarian malignant tumor, fill in the "other malignant tumors" in the malignant tumor, and the code is 030. For the disease symptoms and signs are not clear and the disease diagnosis cannot be made 999.
