# Supplementary material for: A study on the factors influencing the vulnerability of women of childbearing age to health poverty in rural western China
Source: Sci Rep. 2024 Jun 8;14:13219. doi: 10.1038/s41598-024-64070-z (PMC11162415; doi:10.1038/s41598-024-64070-z)
Supplement: Supplementary file 2 — Supplementary Information 2. [file 41598_2024_64070_MOESM2_ESM.doc]

**Note: 注意**

1、set variable value 999 as missing （变量值999设为 缺失值，除非问卷另有说明）

2、As there’re some data entry mistakes, check the variables first to see if there any extremes.

For the variables of expenditure and amount of services: replace the 1% highest cases with missing.

（在分析之前，先核查每一变量是否有特异值以及罗辑错误。对家庭收入、费用支出和获得服务量等连续型变量，将1%的最大值设为缺失值。）

3、For Education Level (A7), the frequencies of categories (4)-(7) are very small. We’d better merge categories (4)-(7) into category (4) which represents the senior high school and above.

（A7文化程度：因为选择(4)-(7)的比例比较小，所以可以把(4)-(7)合并为一个分类(4)-高中及以上）

4、Income: generate new variables

The household income=sum of the consumption expenditure=J13*12+J14+J15+J16(amount consumed by themselves*price)

Income per capita=the household income /number of people in the family

Income level: divide the income per capita into 5 groups, from the 20% lowest to the 20% highest

(收入的计算：生成新的变量

家庭收入=家庭消费性支出之和= J13*12+J14+J15+J16(自己消费数量*价格)—此处请根据问卷选择具体的变量

家庭人均收入=家庭收入/家庭常住人口数

收入水平：按家庭人均收入分为5组:

Level 1: 20% the lowest 收入最低的20%，

Level 2: 20%-40%，

Level 3: 40%-60%，

Level4: 60%-80%，

Level 5: 20% the highest收入最高的20%)

数据库中，家庭问卷只对户做了编码，而没有调查表中的被调查成员编码。

数据库中被调查成员编码已补充

1. **Outpatient （门诊）**
   1. **% of being ill in the last two weeks（过去两周患病率）; among the ill: %seek care, %self tx, %no tx（在患病者中：就诊率、自我医疗率、未治疗率）**

1)Variables:

B1 调查前14天，您是否有身体不适

B7 患病后，是否进行了治疗

B8 您是如何治疗的

2)% of being ill（患病率）: B1=1

%seek care among the ill（患病就诊率）: B8=1｜B8=2 if B1=1

%self tx among the ill（患病自我医疗率）: B8=3|B8=1 if B1=1

%no tx among the ill（患病未治疗率）: B7=2 if B1=1

请注意：就诊率、自我医疗率和未治疗率计算的分母都是患病人数。

|  | Freq（人数） | Percent（人数率）% | 人次数 | 人次率% |
| --- | --- | --- | --- | --- |
| % of being ill | 1973 | 17.1 |  |  |
| %seek care | 767 | 38.9 | 1230 | 62.3 |
| %self tx, | 1085 | 55.0 |  |  |
| %no tx | 493 | 25.0 |  |  |

- 1. **Among those with no tx: % distribution of reasons（患病未治疗者未治疗原因）**

1)variables:B7.1 如未治疗，最主要的原因是什么

2)tab B7.1 if B1=1 & B7=2

|  | Freq | Percent |
| --- | --- | --- |
| 1 自感病情 | 124 | 25.2 |
| 2 经济困难 | 283 | 57.4 |
| 3 无时间 | 15 | 3.0 |
| 4 交通不便 | 4 | 0.8 |
| 5 无有效措施 | 34 | 6.9 |
| 6 其它 | 33 | 6.7 |

- 1. **Among those who seek care:（就诊者）**

Only analyze the first time.（只分析第一次就诊）

- - 1. **Where?（就诊流向）**

1)Variables:

B12 第一次就诊是在哪里？

请宁夏医院学的老师对就诊单位进行编码：将就诊机构分为：1 村卫生室 2 私人诊所 3 乡镇卫生院 4 县医院 5 县级以上医院 6 其它

数据库中就诊单位编码已补充

2)tab B12 if B1=1 & B7=1 & B8!=3

|  | Freq | Percent |
| --- | --- | --- |
| 1 村卫生室 | 151 | 19.7 |
| 2 私人诊所 | 83 | 10.8 |
| 3 乡镇卫生院 | 190 | 24.8 |
| 4 县医院 | 233 | 30.4 |
| 5 县级以上医院 | 66 | 8.6 |
| 6 其它 | 44 | 5.7 |

- - 1. **THE, Saving account, reimbursement, OOP by where.（就诊总支出，家庭账户支付，报销，自付）**

1)Variables:

B14 此次就诊医疗费总额多少元

B14.1 此次就诊医疗费用现金自付多少元

B14.2 家庭账户支付多少元

B14.3 新农合报销或减免多少元

2)Total expenditure: B14 if B1=1 & B7=1 & B8!=3

Saving accout: B14.2 if B1=1 & B7=1 & B8!=3

Reimbursement: B14.3 if B1=1 & B7=1 & B8!=3

Out of pocket: B14.1 if B1=1 & B7=1 & B8!=3

Please do the logical checking if B14=B14.2+B14.3+B14.1（请做逻辑校对：是否B14=B14.2+B14.3+B14.1，列出是与否的比例

除缺失值外，B14=B14.2+B14.3+B14.1

合计表 ：

原始数据计算：

|  | Total  expenditure | Out of  pocket | Saving  account | Reimburse-ment |
| --- | --- | --- | --- | --- |
| Mean | 542.9 | 523.5 | 12.6 | 30.9 |
| Median | 150.0 | 150.0 | 0.0 | 0.0 |
| miss | 22 | 26 | 90 | 88 |

就诊医疗费总额, 就诊医疗费用现金自付金额，就诊家庭账户支付金额和新农合报销或减免金额数据缺失主要是由于以下几个原因。第一，被调查者记不清。第二，农民的理解程度有限对于家庭账户的理解不清。第三，部分农民对于新农合报门诊统筹销减免相关的政策不清楚。

按就诊机构：By where: By B12

|  |  | Total  expenditure | Out of  pocket | Saving  account | Reimburse-  ment |
| --- | --- | --- | --- | --- | --- |
| 1 村卫生室 | Mean | 153.9 | 152.9 | 1.3 | 0.05 |
|  | Median | 80.0 | 80.0 | 0.0 | 0.0 |
| 2 私人诊所 | Mean | 248.3 | 251.4 | 0.05 | 0.6 |
|  | Median | 150.0 | 160.0 | 0.0 | 0.0 |
| 3 乡镇卫生院 | Mean | 257.7 | 268.8 | 6.2 | 5.1 |
|  | Median | 100.0 | 100.0 | 0.0 | 0.0 |
| 4 县医院 | Mean | 597.3 | 551.4 | 2.5 | 55.0 |
|  | Median | 230.0 | 200.0 | 0.0 | 0.0 |
| 5 县级以上医院 | Mean | 2362.6 | 2271.0 | 115.2 | 156.5 |
|  | Median | 600.0 | 600.0 | 0.0 | 0.0 |
| 6 其它 | Mean | 782.5 | 711.6 | 0.0 | 1.2 |
|  | Median | 200.0 | 200.0 | 0.0 | 0.0 |

- 1. **Among self tx（自我医疗者）:**
     1. **% distribution of reasons（自我医疗原因）**

1)Variables: B9 您为什么选择自我医疗

2)tab B9 if B1=1 & B7=1 & (B8=1|B8=3)

|  | Freq | Percent（%） |
| --- | --- | --- |
| 1 按医生的处方进行自我治疗 | 563 | 51.9 |
| 2 自感病轻/没必要看医生 | 187 | 17.2 |
| 3 自我医疗比较便宜 | 203 | 18.7 |
| 4 无时间 | 11 | 1.0 |
| 5 交通不便 | 26 | 2.4 |
| 6 服务差 | 2 | 0.2 |
| 7 其它 | 93 | 8.6 |

- - 1. **药品来源**

1)Variables: B10 如果是自我医疗，您吃药的来源

2) B10 if B1=1 & B7=1 & (B8=1|B8=3)

在数据库中，多选题是每一个选项设为一个变量，变量名和变量标签可能没有标明是属于哪个题目，分析时请小心。

|  | Percent of cases |
| --- | --- |
| 1 家里已有的 | 12.6%(137) |
| 2 药店买的 | 53.7%(583) |
| 3 医疗机构买的（未就诊） | 43.1%(467) |
| 4 别人给的 | 1.0%(11) |
| 5 其它 | 1.1%(12) |

- - 1. **THE（自我医疗总费用）**

1)Variables: B10.1 若在药店或医疗机构购买，您在这14天内一共购买药品的花费是多少元？

2)THE: B10.1 if B1=1 & B7=1 & (B8=1|B8=3) & (B10=2|B10=3)

|  | Total expenditure |
| --- | --- |
| Mean | 209.98 |
| Median | 70.00 |
| miss | 105例（被调查者记不清数据，数值变量无法用999记录数据） |

- 1. **Top 5 health problems by level（前五位疾病）**

1)Variables:

B3.2 您患的是什么病或伤-疾病编码 （use the disease code table 请查阅疾病编码表确定疾病名称）

B12 第一次就诊是在哪里？

2)tab B3.2 if B1=1

按照就诊疾病的第一位名称排列顺位

| Order顺位 | Name of disease 疾病名称 | Percent（%） |
| --- | --- | --- |
| 1health problem | 070（急性鼻咽炎） | 1.9 |
| 2health problem | 112(其他运动疾病) | 1.2 |
| 3health problem | 080（急慢性胃肠炎） | 1.1 |
| 4health problem | 086（胆结石和胆囊炎） | 1.0 |
| 5health problem | 066（高血压） | 0.8 |
| 6health problem | 097（其他女性生殖器官疾病） | 0.8 |
|  | 999（体征、症状和不明情况） | 1.9 |

By level(前五位疾病的就诊流向) : by B12

if B1=1 & B7=1 & B8!=3

row percent（%）

| Name of disease | Village Clinics | Private Clinics | Township Hospitals | County Hospitals | County Hospitals above | Others |
| --- | --- | --- | --- | --- | --- | --- |
| 1health problem | 49.5 | 5.5 | 27．5 | 16.5 | 0 | 1.1 |
| 2health problem | 22.2 | 16.7 | 36.1 | 22．2 | 0 | 2.8 |
| 3health problem | 10 | 8.3 | 43.3 | 26.7 | 8.3 | 3.3 |
| 4health problem | 10.9 | 9.1 | 23.6 | 47.3 | 3.6 | 5.5 |
| 5health problem | 29.7 | 2.7 | 16.2 | 40.5 | 10.8 | 0 |
| 6health problem | 8.2 | 18.4 | 16.3 | 42.9 | 2.0 | 12.2 |
| 999 | 15.7 | 13.7 | 13.7 | 47.1 | 5.9 | 3.9 |

- 1. **% of being ill in the last two weeks; among the ill: %seek care, %self tx, %no tx by income（不同收入组的两周患病率、患病就诊率、患病自我医疗率、患病未治疗率）**

1)Variables:

B1 调查前14天，您是否有身体不适

B7 患病后，是否进行了治

B8 您是如何治疗的

2)By income level:

% of being ill（患病率）: B1=1

%seek care among the ill（患病就诊率）: B8=1｜B8=2 if B1=1 & B7=1

%self tx among the ill（患病自我医疗率）: B8=3 if B1=1 & B7=1

%no tx among the ill（患病未治疗率）: B7=2 if B1=1

分析下表时，收入将1%最大值设为缺失值

| Income | % of being ill | %seek care | %self tx | %no tx |
| --- | --- | --- | --- | --- |
| Level 1 | 15.3 | 31.3 | 52.4 | 28.7 |
| Level 2 | 16.1 | 33.0 | 50.5 | 32.7 |
| Level 3 | 17.7 | 40.9 | 54.4 | 24.8 |
| Level 4 | 19.3 | 43.6 | 55.8 | 20.1 |
| Level 5 | 16.9 | 43.9 | 61.8 | 20.1 |

- 1. **THE, saving, reimbursement, OOP by income level（不同收入组的就诊总费用，家庭账户支出，报销，自付）**

**就诊者 seeking care：**

1)Variables:

B14 此次就诊医疗费总额多少元

B14.1 此次就诊医疗费用现金自付多少元

B14.2 家庭账户支付多少元

B14.3 新农合报销或减免多少元

2)Total expenditure: B14 if B1=1 & B7=1 & B8!=3

Saving accout: B14.2 if B1=1 & B7=1 & B8!=3

Reimbursement: B14.3 if B1=1 & B7=1 & B8!=3

Out of pocket: B14.1 if B1=1 & B7=1 & B8!=3

分析下表时，收入将1%最大值设为缺失值

| Income |  | Total  expenditure | Out of  pocket | Saving  account | Reimbursement |
| --- | --- | --- | --- | --- | --- |
| Level 1 | Mean | 425.4 | 390.0 | 4.6 | 40.9 |
|  | Median | 80.0 | 80.0 | 0.0 | 0.0 |
| Level 2 | Mean | 569.7 | 513.0 | 3.0 | 46.7 |
|  | Median | 200.0 | 190.0 | 0.0 | 0.0 |
| Level 3 | Mean | 455.3 | 431.6 | 4.1 | 3.3 |
|  | Median | 180.0 | 150.0 | 0.0 | 0.0 |
| Level 4 | Mean | 469.7 | 451.8 | 1.2 | 30.3 |
|  | Median | 160.0 | 160.0 | 0.0 | 0.0 |
| Level 5 | Mean | 764.4 | 787.3 | 43.2 | 40.1 |
|  | Median | 200.0 | 200.0 | 0.0 | 0.0 |

**自我医疗者 self treatment**：

THE: B10.1 if B1=1 & B7=1 & (B8=1|B8=3) & (B10=2|B10=3)

分析下表时，收入将1%最大值设为缺失值

| Income | Mean（元） | Median（元） |
| --- | --- | --- |
| Level 1 | 106.6 | 50.0 |
| Level 2 | 181.7 | 60.0 |
| Level 3 | 174.3 | 80.0 |
| Level 4 | 301.9 | 100.0 |
| Level 5 | 251.2 | 100.0 |

1. **Inpatient（住院）**
   1. **% of needing hospital care in the last year; among the ill: %seek care, %no tx（应住院率；在需住院者中：住院率和应住院未住院率）**

1)Variables:

C1 过去一年中是否有医生诊断您需要住院的情况？

C2 过去一年中医生诊断您需要住院而您未住院的次数？

C3 过去一年中，您一共住过几次院？

2)% of needing hospital care: C1=1

% seek care: C3>0 if C1=1

% no tx: C2>0 if C1=1

|  | 人数 | 人次数 | 人数率（%） | 人次率（%） |
| --- | --- | --- | --- | --- |
| % of needing hospital care | 867 | 1333 | 7.5 | 11.58 |
| %seek care, | 749 | 965 | 86.4 | 111.30 |
| %no tx | 118 | 368 | 13.6 | 42.45 |

- 1. **Among those with no tx: % distribution of reasons（在应住院未住院者中，未住院原因）**

1)Variables: C2.1 未住院主要原因

2) tab C2.1 if C1=1 & C2>0

调查表中的标签标错：(5)价格太高，(5)没床位，(6)其他

核查一下数据库中是否已经改过来。

（在建立数据库时已发现这个问题，但文卷中是直接填入所选数字，无法区分5还是6，录入资料时按照问卷录入的）

|  | Freq | Percent |
| --- | --- | --- |
| 1没必要 | 8 | 4.1 |
| 2没时间 | 9 | 4.6 |
| 3经济困难 | 161 | 82.6 |
| 4服务差 | 1 | .5 |
| 5价格太高及没床位 | 6 | 3.1 |
| 6其他 | 10 | 5.1 |

- 1. **Among those who seek care:（住院者）**

Only analyze last hospital care.（仅分析最近一次住院情况）

- - 1. **Where?（住院机构）**

1)Variables:

C4.2 本次住院的医疗单位名称是？

请宁夏医学院的老师对住院单位名称编码：

1 乡镇卫生院 2 县医院 3 县级以上医院 4 其它

数据库中已添加住院单位编码

2) tab C4.2 if C1=1 & C3>0

|  | Freq | Percent |
| --- | --- | --- |
| 1 乡镇卫生院 | 163 | 21.8 |
| 2县医院 | 399 | 53.3 |
| 3 县级以上医院 | 146 | 19.5 |
| 4 其它 | 41 | 5.5 |

- - 1. **THE, reimbursement, OOP by where.（不同住院机构的医疗总费用，新农合报销，自付）**

1)Variables:

C4.8 过去一年，你是否已参加了新农合？

C4.8.1如果参加了新农合，缴费方式是？

C4.9 本次住院医疗费用您自己先垫付了多少元？

C4.9.1本次住院医疗费用合管中心给你报销了多少元？

C4.10本次住院医疗费用您自己支付了多少元

C4.11 因本次住院所花费的车旅费、营养伙食费、陪护费？

C4.12住院期间，您或几人是否向医护人员送礼或送红包？

C4.12.1 如有，共多少钱？

2) If C1=1 & C3>0:

*Medical Expenditure:（医疗支出）

If C4.8=1 & C4.8.1=1:

THE=C4.9

Reimbursement=C4.9.1

OOP=C4.9-C4.9.1

If C4.8=1 & C4.8.1=2:

OOP=C4.10

If C4.8=2:

THE=OOP=C4.10

*Other Expenditure（其它支出）

C4.11 所花费的车旅费、营养伙食费、陪护费

C4.12.1 送礼或红包费 if C4.12!=4

Please check:

How many C4.9, C4.9.1, C4.10 are answered while C4.8 or C4.8.1 are missing?

And if C4.9, C4.9.1, C4.10 are all answered, does C4.9-C4.9.1=C4.10?

请核查：

有多少C4.8或C4.8.1缺失，而C4.9,C4.9.1,C4.10仍回答了的？

没有C4.8或C4.8.1缺失的情况

如果C4.9,C4.9.1,C4.10全部回答了的话，是否C4.9-C4.9.1=C4.10?

是按照这种逻辑关系调查并输入数据的

**合计表**：

If C4.8=1 & C4.8.1=1

|  | Medical Expenditure  (医疗支出) | | | Other Expenditure  （其它支出） | |
| --- | --- | --- | --- | --- | --- |
|  | Total  expenditure | Reimburse-  ment | Out of  pocket | C4.11车旅费等 | C4.12礼品费等 |
| Mean | 3215.09 | 835.66 | 2382.60 | 645.08 | 211.43 |
| Median | 1863.41 | 260.00 | 1000.00 | 300.00 | 175.00 |

If C4.8=1 & C4.8.1=2 or If C4.8=2

|  | Medical Expenditure  ---OOP  (医疗支出) | Other Expenditure  （其它支出） | |
| --- | --- | --- | --- |
|  |  | C4.11车旅费等 | C4.12礼品费等 |
| Mean | 1955.53 | 736.18 | 902.50 |
| Median | 500.00 | 300.00 | 375.00 |

**按不同住院机构分组**：

If C4.8=1 & C4.8.1=1

|  |  | Medical Expenditure(医疗支出) | | | Other Expenditure  （其它支出） | |
| --- | --- | --- | --- | --- | --- | --- |
|  |  | Total  expenditure | Reimburse-  ment | Out of  pocket | C4.11车旅费等 | C4.12礼品费等 |
| 1 乡镇卫生院 | Mean | 1093.46 | 325.67 | 767.79 | 258.78 | 203.33 |
|  | Median | 600.00 | 150.00 | 300.00 | 150.00 | 200.00 |
| 2 县医院 | Mean | 2675.66 | 1044.09 | 1635.19 | 601.94 | 150.00 |
|  | Median | 2000.00 | 500.00 | 1000.00 | 400.00 | 100.00 |
| 3 县级以上医院 | Mean | 6674.60 | 974.93 | 5699.68 | 1042.07 | 500.00 |
|  | Median | 3537.78 | 210.00 | 3000.00 | 500.00 | 500.00 |
| 4 其它 | Mean | 3954.73 | 121.21 | 3833.52 | 1105.48 | 0.00 |
|  | Median | 2460.00 | 0.00 | 2100.00 | 3500.00 | 0.00 |

If C4.8=1 & C4.8.1=2 or If C4.8=2

|  |  | Medical Expenditure  ---OOP(医疗支出) | Other Expenditure（其它支出） | |
| --- | --- | --- | --- | --- |
|  |  |  | C4.11车旅费等 | C4.12礼品费等 |
| 1 乡镇卫生院 | Mean | 440.23 | 176.96 | 0.00 |
|  | Median | 300.00 | 65.00 | 0.00 |
| 2 县医院 | Mean | 1533.02 | 608.00 | 375.00 |
|  | Median | 1000.00 | 300.00 | 375.00 |
| 3 县级以上医院 | Mean | 5577.79 | 2018.75 | 1430.00 |
|  | Median | 3900.00 | 700.00 | 1430.00 |
| 4 其它 | Mean | 1290.00 | 1000.00 | 0.00 |
|  | Median | 370.00 | 1000.00 | 0.00 |

- 1. **Top 5 health problems by level（前五位疾病）**

1)Variables:

C4 您因疾病或损伤中毒等住院的疾病名称？

（Please use the disease code table 请查阅疾病编码表确定疾病名称）

2)tab C4 if C1=1 & C3>0

**合计表：**

| Order顺位 | Name of disease 疾病名称 | Percent（%） |
| --- | --- | --- |
| 1health problem | 胆结石症和胆囊炎086 | 8.3 |
| 2health problem | 阑尾疾病082 | 6.4 |
| 3health problem | 正常分娩102 | 6.3 |
| 4health problem | 乙型肝炎012 | 3.6 |
| 5health problem | 急、慢性胃肠炎080 | 3.6 |
| 6health problem | 其他女性生殖器官疾病097 | 3.5 |
| 7health problem | 999 | 9.9 |

**前五位疾病的住院机构流向**：

C4， row percent

| Name of disease | Township Hospitals | County Hospitals | County Hospitals above | Others |
| --- | --- | --- | --- | --- |
| 1health problem | 19 | 35 | 6 | 2 |
| (percent) | 30.6% | 56.5% | 9.7% | 3.2% |
| 2health problem | 12 | 32 | 3 | 1 |
| (percent) | 25.0% | 66.7% | 6.3% | 2.1% |
| 3health problem | 13 | 31 | 3 | 0 |
| (percent) | 27.7% | 66.0% | 6.4% | .0% |
| 4health problem | 2 | 18 | 6 | 1 |
| (percent) | 7.4% | 66.7% | 22.2% | 3.7% |
| 5health problem | 4 | 16 | 3 | 4 |
| (percent) | 14.8% | 59.3% | 11.1% | 14.8% |
| 6health problem | 11 | 10 | 2 | 3 |
| (percent) | 42.3% | 38.5% | 7.7% | 11.5% |
| 7health problem | 22 | 33 | 17 | 2 |
| (percent) | 29.7% | 44.6% | 23.0% | 2.7% |

- 1. **% of needing hospital care in the last year; among the ill: %seek care, %no tx by income （不同收入组的应住院率；在应住院者中：住院率，应住院未住院率）**

1)Variables:

C1 过去一年中是否有医生诊断您需要住院的情况？

C2 过去一年中医生诊断您需要住院而您未住院的次数？

C3 过去一年中，您一共住过几次院？

2)By income level:

% of needing hospital care: C1=1

% seek care: C3>0 if C1=1

% no tx: C2>0 if C1=1

分析下表时，收入将1%最大值设为缺失值

| Income | % of needing  Hospital care | %seek care | %no tx |
| --- | --- | --- | --- |
| Level 1 | 127（14.8%） | 116（15.7%） | 23（11.9%） |
| Level 2 | 148（17.3%） | 124（16.8%） | 39（20.2%） |
| Level 3 | 181（21.1%） | 148（20.0%） | 51（26.4%） |
| Level 4 | 218（25.5%） | 191（25.8%） | 40（20.7%） |
| Level 5 | 182（21.3%） | 160（21.7%） | 40（20.7%） |

- 1. **THE, reimbursement, OOP by income level（不同收入组的医疗支出、报销、自付）**

1)Variables:

C4.8 过去一年，你是否已参加了新农合？

C4.8.1如果参加了新农合，缴费方式是？

C4.9 本次住院医疗费用您自己先垫付了多少元？

C4.9.1本次住院医疗费用合管中心给你报销了多少元？

C4.10本次住院医疗费用您自己支付了多少元

C4.11 因本次住院所花费的车旅费、营养伙食费、陪护费？

C4.12住院期间，您或几人是否向医护人员送礼或送红包？

C4.12.1 如有，共多少钱？

Income Level

2) If C1=1 & C3>0:

*Medical Expenditure:（医疗支出）

If C4.8=1 & C4.8.1=1:

THE=C4.9

Reimbursement=C4.9.1

OOP=C4.9-C4.9.1

If C4.8=1 & C4.8.1=2:

OOP=C4.10

If C4.8=2:

THE=OOP=C4.10

*Other Expenditure（其它支出）

C4.11 所花费的车旅费、营养伙食费、陪护费

C4.12.1 送礼或红包费

If C4.8=1 & C4.8.1=1

分析下表时，收入将1%最大值设为缺失值

|  |  | Medical Expenditure(医疗支出) | | | Other Expenditure  （其它支出） | |
| --- | --- | --- | --- | --- | --- | --- |
|  |  | Total  expenditure | Reimburse-  ment | Out of  pocket | C4.11车旅费等 | C4.12礼品费等 |
| Level 1 | Mean | 1996.09 | 458.27 | 1537.82 | 433.67 | 3.93 |
|  | Median | 1060.00 | 150.00 | 700.00 | 200.00 | 4.00 |
| Level 2 | Mean | 2185.65 | 785.95 | 1399.71 | 488.05 | 3.97 |
|  | Median | 1300.00 | 300.00 | 700.00 | 225.00 | 4.00 |
| Level 3 | Mean | 2751.03 | 1003.27 | 1747.76 | 517.63 | 3.91 |
|  | Median | 2000.00 | 300.00 | 1000.00 | 300.00 | 4.00 |
| Level 4 | Mean | 3139.57 | 811.57 | 2328.00 | 752.20 | 3.92 |
|  | Median | 2000.00 | 300.00 | 1100.00 | 350.00 | 4.00 |
| Level 5 | Mean | 4469.20 | 1013.63 | 3478.20 | 862.48 | 3.91 |
|  | Median | 2000.00 | 300.05 | 1500.00 | 500.00 | 4.00 |

If C4.8=1 & C4.8.1=2 or If C4.8=2

分析下表时，收入将1%最大值设为缺失值

|  |  | Medical Expenditure  ---OOP(医疗支出) | Other Expenditure（其它支出） | |
| --- | --- | --- | --- | --- |
|  |  |  | C4.11车旅费等 | C4.12礼品费等 |
| Level 1 | Mean | 363.67 | 178.33 | 4.00 |
|  | Median | 300.00 | 100.00 | 4.00 |
| Level 2 | Mean | 1040.63 | 1829.23 | 4.00 |
|  | Median | 275.00 | 300.00 | 4.00 |
| Level 3 | Mean | 2428.57 | 581.54 | 4.00 |
|  | Median | 1500.00 | 300.00 | 4.00 |
| Level 4 | Mean | 1295.52 | 419.05 | 3.88 |
|  | Median | 1000.00 | 400.00 | 4.00 |
| Level 5 | Mean | 3634.12 | 1168.13 | 3.79 |
|  | Median | 2500.00 | 650.00 | 4.00 |

1. **Chronic（慢性病）**
   1. **% of having a chronic disease; among the ill: %seek care（慢性病患病率，患病就诊率）**

1)Variables:

D1 过去半年内您是否患有经医生诊断的慢性疾病

D2 最近三个月，您因这些疾病看过几次病

2)% of having a chronic disease: D1=1

%seek care:% of D2>0 if D1=1

|  | Freq | 人次 | Percent（%） | 人次（%） |
| --- | --- | --- | --- | --- |
| % of having a chronic disease | 1528 |  | 13.3 |  |
| %seek care, | 707 | 2001 | 46.3 | 130.96% |

- 1. **Among those who seek care:（就诊者）**
     1. **Where?（就诊流向）**

1)Variables:

D3.1 最近三个月，您因这些疾病就诊的主要机构是？

2)tab D3.1 if D1=1 & D2>0

|  | Freq | Percent（%） |
| --- | --- | --- |
| 1 村卫生室 | 93 | 13.2 |
| 2 乡镇卫生院 | 193 | 27.3 |
| 3 县医院 | 314 | 44.4 |
| 4 私人诊所 | 61 | 8.6 |
| 5 其他 | 46 | 6.5 |

- - 1. **THE, Saving account, reimbursement, OOP by where.（不同就诊机构的医疗支出，家庭账户支付，报销，自付）**

1)Variables:

D3.2 最近三个月内，您是否已参加新农合

D3.2.1如果参加了新农合，缴费方式是

D3.3 医疗费用您自己先垫付了多少元

D3.3.1医疗费用家庭账户支付了多少元？

D3.3.2医疗费用合管中心给报销了多少元

D3.4 医疗费用您自己支付了多少元

2) If D1=1 & D2>0:

If D3.2=1 & D3.2.1=1:

THE=D3.3

Saving Account=D3.3.1

Reimbursement=D3.3.2

OOP=D3.3-D3.3.1-D3.3.2

If D3.2=1 & D3.2.1=2:

OOP=D3.4

If D3.2=2:

OOP=D3.4

Please check:

How many D3.3, D3.3.1, D3.3.2,D3.4 are answered while D3.2 or D3.2.1 are missing?

And if D3.3, D3.3.1, D3.3.2,D3.4 are all answered, does D3.3-D3.3.1-D3.3.2=D3.4?

请核查：

有多少D3.2或D3.2.1缺失，而D3.3,D3.3.1,D3.3.2,D3.4仍回答了的？

D3.2没有缺失，D3.2.1 缺失原因：⑴被调查者不清楚2009年元月开始实施的慢性病统筹政策，无法回答这个选项，⑵病人是全部自付的，⑶病人是用以前家庭账户剩余的钱支付的，病人自己不清楚。

（注：问卷回答是D3.3和D3.3.1数据相同，表示就诊者直接用家庭账户看病，如果按表调查，则D3.2.1应回答2，直接跳问到D3.4，那么D3.4直接为0，体现不出病人就诊花费为多少。）

如果D3.3,D3.3.1,D3.3.2,D3.3.4全部回答了的话，是否D3.3-D3.3.1-D3.3.2=D3.4?

我们调查是在2月底，海原是2009年1月实行慢性病统筹的，农民基本不知道慢性病可以报销，在调查时，学生认为D3.3= D3.4，录入人员直接把四项都录入，现在做核查时，调出原始问卷，理清逻辑关系后对数据进行清理，我们将这部分问题按D3.3=D3.3.1+D3.3.2，进行录入！如回答D3.4，那么前三项空缺！

**合计表**：

If D3.2 & D3.2.1=1（分析时包括了D 3.2.1部分的缺失值）

|  | Total  expenditure | Saving account | Reimburse-  ment | Out of  pocket |
| --- | --- | --- | --- | --- |
| Mean | 961.6319 | 191.9472 | 94.4120 | 961.6319 |
| Median | 400.0000 | .0000 | .0000 | 400.0000 |

If D3.2=1 & D3.2.1=2 or If D3.2=2（分析时包括了D 3.2.1部分的缺失值）

|  | Out of  pocket |
| --- | --- |
| Mean | 856.3171 |
| Median | 300.0000 |

**按不同就诊机构分组**：By where: D3.2

If D3.2 & D3.2.1=1

|  |  | Total  expenditure | Saving account | Reimburse-  ment | Out of  pocket |
| --- | --- | --- | --- | --- | --- |
| 1村卫生室 | Mean | 441.0395 | 52.9730 | 15.7895 |  |
|  | Median | 300.0000 | .0000 | .0000 |  |
| 2乡镇卫生院 | Mean | 501.1875 | 56.3057 | 42.8428 |  |
|  | Median | 200.0000 | .0000 | .0000 |  |
| 3县医院 | Mean | 1310.7462 | 345.0583 | 146.1989 |  |
|  | Median | 600.0000 | .0000 | .0000 |  |
| 4私人诊所 | Mean | 808.9815 | 69.0741 | 44.0556 |  |
|  | Median | 300.0000 | .0000 | .0000 |  |
| 5其他 | Mean | 1676.5854 | 156.8049 | 176.2650 |  |
|  | Median | 450.0000 | .0000 | .0000 |  |

If D3.2=1 & D3.2.1=2 or If D3.2=2

|  |  | Out of  pocket |
| --- | --- | --- |
| 1村卫生室 | Mean | 724.2857 |
|  | Median | 350.0000 |
| 2乡镇卫生院 | Mean | 450.8056 |
|  | Median | 260.0000 |
| 3县医院 | Mean | 1091.6897 |
|  | Median | 360.0000 |
| 4私人诊所 | Mean | 617.0000 |
|  | Median | 100.0000 |
| 5其他 | Mean | 1894.0000 |
|  | Median | 300.0000 |

**D3.5 在药店自行购药费用**。

If D1=1

|  | 自行购药费用 |
| --- | --- |
| Mean | 449.7786 |
| Median | 120.0000 |

- 1. **Top 5 health problems by level**

1)Variables: D1.1 如有，是哪些疾病？

（Please use the disease code table 请查阅疾病编码表确定疾病名称）

2) tab D1.1 if D1=1

（按照第一种最严重的慢性病排列疾病顺位）

| Order顺位 | Name of disease 疾病名称 | fre | Percent |
| --- | --- | --- | --- |
| 1health problem | 高血压066 | 227 | 2.0 |
| 2health problem | 急慢性胃肠炎080 | 138 | 1.2 |
| 3health problem | 胆结石和胆囊炎086 | 123 | 1.1 |
| 4health problem | 乙型肝炎012 | 107 | 0.9 |
| 5health problem | 类风湿性关节炎109 | 83 | 0.7 |

- 1. **% of having a chronic disease in the last half year; among the ill: %seek care by income**

1)Variables:

D1 过去半年内您是否患有经医生诊断的慢性疾病

D2 最近三个月，您因这些疾病看过几次病

2)By income:

% of having a chronic disease: D1=1

%seek care:% of D2>0 if D1=1

分析下表时，将收入1%最大值设为缺失值

| Income Level | 人数 | % of having chronic disease | 人数 | % of seeking care |
| --- | --- | --- | --- | --- |
| Level 1 | 298 | 12.9% | 103 | 34.6% |
| Level 2 | 284 | 12.4% | 118 | 41.5% |
| Level 3 | 299 | 13.0% | 134 | 44.8% |
| Level 4 | 369 | 16.1% | 197 | 53.4% |
| Level 5 | 266 | 12.2% | 147 | 55.3% |

- 1. **THE, saving, reimbursement, OOP,income by income level**

1)Variables:

D3.2 最近三个月内，您是否已参加新农合

D3.2.1如果参加了新农合，缴费方式是

D3.3 医疗费用您自己先垫付了多少元

D3.3.1医疗费用家庭账户支付了多少元？

D3.3.2医疗费用合管中心给报销了多少元

D3.4 医疗费用您自己支付了多少元

2) If D1=1 & D2>0:

If D3.2=1 & D3.2.1=1:

THE=D3.3

Saving Account=D3.3.1

Reimbursement=D3.3.2

OOP=D3.3-D3.3.1-D3.3.2

If D3.2=1 & D3.2.1=2:

OOP=D3.4

If D3.2=2:

OOP=D3.4

按人均收入水平分组：by income level

分析下表时，将收入1%最大值设为缺失值

If D3.2 & D3.2.1=1

|  |  | Total  expenditure | Saving account | Reimburse-  ment | Out of  pocket |
| --- | --- | --- | --- | --- | --- |
| Level 1 | Mean | 469.0741 | 51.6138 | 80.5432 |  |
|  | Median | 300.0000 | .0000 | .0000 |  |
| Level 2 | Mean | 663.4455 | 168.4495 | 116.4771 |  |
|  | Median | 300.0000 | .0000 | .0000 |  |
| Level 3 | Mean | 738.2328 | 201.7391 | 17.9478 |  |
|  | Median | 300.0000 | .0000 | .0000 |  |
| Level 4 | Mean | 1098.9636 | 124.4063 | 69.4878 |  |
|  | Median | 460.0000 | .0000 | .0000 |  |
| Level 5 | Mean | 1612.99 | 396.50 | 189.930 |  |
|  | Median | 500.0000 | .0000 | .0000 |  |

If D3.2=1 & D3.2.1=2 or If D3.2=2

分析下表时，将收入1%最大值设为缺失值

| Income |  | Out of  pocket |
| --- | --- | --- |
| Level 1 | Mean | 530.4091 |
|  | Median | 310.0000 |
| Level 2 | Mean | 458.0769 |
|  | Median | 300.0000 |
| Level 3 | Mean | 583.9000 |
|  | Median | 280.0000 |
| Level 4 | Mean | 669.0909 |
|  | Median | 230.0000 |
| Level 5 | Mean | 1538.91 |
|  | Median | 415.000 |

**D3.5 在药店自行购药费用** By income level

If D1=1

分析下表时，将收入1%最大值设为缺失值

| Income |  | 自行购药费用 |
| --- | --- | --- |
| Level 1 | Mean | 242.25 |
|  | Median | 60.0 |
| Level 2 | Mean | 310.9576 |
|  | Median | 120.0000 |
| Level 3 | Mean | 485.3358 |
|  | Median | 200.0000 |
| Level 4 | Mean | 588.6751 |
|  | Median | 150.0000 |
| Level 5 | Mean | 497.38 |
|  | Median | 100.00 |

1. **Catastrophic expenditure**: （风险性卫生支出）
   1. Catastrophic expenditure= OOP (OP+IP+chronic+self tx)/income

1) Variables:

J14.4 家庭支出-药品、医疗费等（只包括自己支付的医药费用金额）

2) Being pressed for time, we will use J14.4 as OOP at this stage.（目前的阶段先使用J14.4作为自付医疗费用）

OOP=J14.4

Income=the household income

Catastrophic expenditure=J14.4/the household income

|  | Catastrophic expenditure |
| --- | --- |
| Mean | 0.1204 |
| Median | 0.0585 |

- 1. Catastrophic expenditure by income level:

| Income | Mean | Median |
| --- | --- | --- |
| Level 1 | 0.1079 | 0.0554 |
| Level 2 | 0.1124 | 0.0632 |
| Level 3 | 0.1245 | 0.0662 |
| Level 4 | 0.1362 | 0.0838 |
| Level 5 | 0.1213 | 0.0353 |

1. **Poverty**

We will send the instructions later.

1. **Prevention**
   1. **MCH [separately for those born last year**

F: 15-49岁已婚育龄妇女 ：A3 性别； A5 年龄； A6 婚姻状况

If A3=2 & A5>=15 & A5<=49 & (A6=2|A6=3|A6=4|A6=5)

**F1 过去一年中，您是否作过妇科检查？**

|  | Freq | Percent（%） |
| --- | --- | --- |
| 1是 | 564 | 36.2 |
| 2否 | 993 | 63.8 |

**F2 您在2003年1月1日以后是否有分娩**？

|  | Freq | Percent（%） |
| --- | --- | --- |
| 1是 | 577 | 37.0 |
| 2否 | 981 | 63.0 |

能否请宁夏医学院的老师说明一下：新农合对分娩费用进行定额补偿的政策是在哪一年开始实施的？是所有的产妇都可得到补助，还是只有在乡镇卫生院分娩的产妇可以得到？补助是到合管中心领取，还是从分娩费用中直接扣除？

咨询了海原县，海原是从2007年1月1日实行新农合，同年也开始实行了对分娩费用的补偿。实施方案中规定所有住院机构都可以补助，补助是从分娩费用中直接减免，（县外医院是自己先垫付）。但海原县是降消项目和瑞典妇幼卫生项目县，另有对住院分娩的补偿，因此在乡镇卫生院分娩的产妇费用基本是全免的，但许多被调查者只知道自己分娩没有花钱，其他一概不知）

下面这张表的数据是根据海原县实施分娩费用补偿的年份，以2007年为界根据孩子年龄推算的。

| 分娩年份 | Freq | Percent（%） |
| --- | --- | --- |
| 新农合对分娩费用进行定额补偿前 | 430 | 44.1 |
| 新农合对分娩费用进行定额补偿后 | 544 | 55.9 |

**F3 最近一次分娩，产前做过几次检查？**

If F2=1

|  | 产前检查次数 |
| --- | --- |
| Mean | 2.16 |
| Median | 2 |
| % of F3=0 | 183（31.9%） |

**F3.1 第一次产前检查是在怀孕第几周时做的**？

If F2=1 & F3>0

|  | 第一次产前检查时间（周） |
| --- | --- |
| Mean | 18 |
| Median | 12 |

**F3.2 您都在哪里做过产前检查？**

If F2=1 & F3>0

|  | Percent（%） |
| --- | --- |
| 1 县/区及以上医院 | 26.3 |
| 2县/区及以上中医院 | 23.5 |
| 3妇幼保健机构 | 7.7 |
| 4乡镇街道卫生院 | 43.5 |
| 5社区卫生服务中心 | 0 |
| 6计划生育指导站 | 0.8 |
| 7卫生室/所/站 | 3.1 |
| 8 其它 | 1.3 |

**F3.3 产前检查项目**？

If F2=1 & F3>0

|  | Freq | Percent（%） |
| --- | --- | --- |
| 1 测量体重 | 113 | 28.9 |
| 2 抽血检查 | 101 | 25.8 |
| 3 测量血压 | 163 | 41.7 |
| 4 尿常规检查 | 133 | 34 |
| 5 B超检查 | 369 | 94.4 |

**F4 分娩地点**？

If F2=1

|  | Freq | Percent（%） |
| --- | --- | --- |
| 1县及以上医院 | 99 | 17.2 |
| 2县及以上中医院 | 41 | 7.1 |
| 3妇幼保健机构 | 35 | 6.1 |
| 4乡镇卫生院 | 125 | 21.8 |
| 5社区卫生服务中心 | 0 | 0 |
| 6计划生育指导站 | 0 | 0 |
| 7卫生室/所/站 | 5 | 0.9 |
| 8家中 | 260 | 45.3 |
| 9其它 | 9 | 1.6 |

此表无法填写，母亲与孩子的编码不同，调查表中未调查最近一次分娩的具体生产日期！

|  | 分娩时间在新农合实施定额补偿前 | 分娩时间在新农合实施定额补偿后 |
| --- | --- | --- |
| 1县及以上医院 |  |  |
| 2县及以上中医院 |  |  |
| 3妇幼保健机构 |  |  |
| 4乡镇卫生院 |  |  |
| 5社区卫生服务中心 |  |  |
| 6计划生育指导站 |  |  |
| 7卫生室/所/站 |  |  |
| 8家中 |  |  |
| 9其它 |  |  |

**F4.1 如在家中分娩，未去医院的最主要原因**

If F2=1 & F4=8

| Income | 1没必要 | % | 2来不及 | % | 3经济困难 | % | 4交通不便 | % | 5其它 | % |
| --- | --- | --- | --- | --- | --- | --- | --- | --- | --- | --- |
| Level 1 | 13 | 18.1 | 22 | 30.6 | 24 | 33.3 | 12 | 16.7 | 1 | 1.4 |
| Level 2 | 17 | 26.6 | 21 | 32.8 | 15 | 23.4 | 8 | 12.5 | 3 | 4.7 |
| Level 3 | 10 | 20.8 | 19 | 39.6 | 10 | 20.8 | 6 | 12.5 | 3 | 6.3 |
| Level 4 | 8 | 20.6 | 8 | 20.5 | 18 | 46.2 | 5 | 12.8 | 0 | 0 |
| Level 5 | 9 | 25.0 | 14 | 38.9 | 8 | 22.2 | 2 | 5.6 | 3 | 8.3 |
| Total | 57 | 22.0 | 84 | 32.4 | 75 | 29.0 | 33 | 12.7 | 10 | 3.9 |

**F4.2 如在家中分娩，接生者是谁？**

If F2=1 & F4=8

|  | Freq | Percent（%） |
| --- | --- | --- |
| 1乡及以上医生 | 4 | 1.5 |
| 2 村医生 | 28 | 10.8 |
| 3 专职接生员 | 51 | 19.6 |
| 4 非专职接生者 | 60 | 23.1 |
| 5 家人自接 | 106 | 40.8 |
| 6 其它 | 11 | 4.2 |

**F5-F5.3 分娩费用**

请问一下宁夏医学院的老师：F5-F5.3，在住院和慢病的医疗支出部分，当缴费方式选1时，回答完自己垫付、报销费用后设置了跳转，这样就不用回答自付费用的问题了。但在分娩费用中，F5.2却没有设置跳转。我们在调查时是怎么做的，是按跳转调查的，还是没有跳转，三个问题都调查了？

这部分问题问卷里没有设置跳转，是按照培训手册的要求，计算同c4.8.1----c4.10，三个问题都做了调查。

If F2=1:

If F5=1:

THE=F5.1

Reimbursement=F5.2

OOP=F5.1-F5.2

If F5=2:

OOP=F5.3

Please check:

How many F5.1, F5.2,F5.3 are answered while F5 is missing?

And if F5.1,F5.2 ,F5.3 are all answered, does F5.1-F5.2=F5.3?

**合计表**：

If F2=1 & F5=1

|  | Total expenditure | Reimbursement | Out of pocket |
| --- | --- | --- | --- |
| Mean | 766.89 | 155.03 | 611.86 |
| Median | 400 | 0 | 250 |

If F2=1 & F5=2

|  | Out of pocket |
| --- | --- |
| Mean | 267.25 |
| Median | 50 |

**不同分娩地点的费用:** By where: F4

If F2=1 & F5=1

|  |  | Total expenditure | Reimbursement | Out of pocket |
| --- | --- | --- | --- | --- |
| 1县/区及以上医院 | Mean | 1454 | 224.81 | 1229.19 |
|  | Median | 530 | 98.5 | 500 |
| 2县/区及以上中医 | Mean | 910.29 | 369.14 | 541.14 |
|  | Median | 540 | 150 | 350 |
| 3妇幼保健机构 | Mean | 702.12 | 149.12 | 553 |
|  | Median | 500 | 125 | 350 |
| 4乡镇街道卫生院 | Mean | 428.52 | 94.03 | 334.49 |
|  | Median | 300 | 0 | 175 |
| 5社区卫生服务中心 | Mean | 0 | 0 | 0 |
|  | Median | 0 | 0 | 0 |
| 6计划生育指导站 | Mean | 0 | 0 | 0 |
|  | Median | 0 | 0 | 0 |
| 7卫生室/所/站 | Mean | 260.67 | 96.33 | 164.33 |
|  | Median | 300 | 0 | 120 |
| 8家中 | Mean | 213.53 | 22.35 | 191.18 |
|  | Median | 100 | 0 | 100 |
| 9其它 | Mean | 280 | 0 | 280 |
|  | Median | 200 | 0 | 200 |

If F2=1 & F5=2

|  |  | Out of pocket |
| --- | --- | --- |
| 1县/区及以上医院 | Mean | 1144.29 |
|  | Median | 200 |
| 2县/区及以上中医 | Mean | 400 |
|  | Median | 400 |
| 3妇幼保健机构 | Mean | 0 |
|  | Median | 0 |
| 4乡镇街道卫生院 | Mean | 93.53 |
|  | Median | 0 |
| 5社区卫生服务中心 | Mean | 0 |
|  | Median | 0 |
| 6计划生育指导站 | Mean | 0 |
|  | Median | 0 |
| 7卫生室/所/站 | Mean | 208 |
|  | Median | 208 |
| 8家中 | Mean | 101.05 |
|  | Median | 0 |
| 9其它 | Mean | 300 |
|  | Median | 300 |

**不同收入水平的分娩费用**：By income level

If F2=1 & F5=1

下表按照收入分层分析时没有将1%的最大值设为缺失值（有效数据较少）

| Income |  | Total expenditure | Reimbursement | Out of pocket |
| --- | --- | --- | --- | --- |
| Level 1 | Mean | 662.67 | 189.18 | 473.49 |
|  | Median | 300 | 50 | 200 |
| Level 2 | Mean | 600.86 | 111.95 | 488.91 |
|  | Median | 300 | 0 | 177.5 |
| Level 3 | Mean | 835.41 | 192.03 | 643.38 |
|  | Median | 500 | 0 | 250 |
| Level 4 | Mean | 777.93 | 147.67 | 630.26 |
|  | Median | 460 | 0 | 350 |
| Level 5 | Mean | 995.86 | 132.07 | 863.79 |
|  | Median | 465 | 0 | 300 |

If F2=1 & F5=2

下表按照收入分层分析时没有将1%的最大值设为缺失值（有效数据较少）

|  |  | Out of pocket |
| --- | --- | --- |
| Level 1 | Mean | 196.25 |
|  | Median | 10 |
| Level 2 | Mean | 388.75 |
|  | Median | 75 |
| Level 3 | Mean | 81.67 |
|  | Median | 25 |
| Level 3 | Mean | 467.29 |
|  | Median | 75 |
| Level 4 | Mean | 83.33 |
|  | Median | 50 |

**F6 产后42天内，您接受产后访视的次数**

F2=1

|  | 平均产后访视次数 | 非在家生产者产后访视次数  （If F4≠8） |
| --- | --- | --- |
| Mean | 0.36 | 0.52 |
| Median | 0 | 0 |
| % of F3=0 | 481（84.1%） | 243（77.4%） |

- 1. **Children**

调查5岁以下儿童：If A5(年龄)<5

按年龄分组：by A5

这部分内容的缺失值，⑴是由于知情人不在或记不清，无法调查，

**G2 近12个月内，孩子接受了几次健康体检？**

|  | G2 健康体检次数 |
| --- | --- |
| Mean | 0.58 |
| Median | 0 |
| % of G2=0 | 596（68.1%） |
| miss | 108 |

**G3 孩子有计划免疫接种卡或手册吗？**

|  | Freq | Percent |
| --- | --- | --- |
| 1有 | 724 | 83.1 |
| 2 没有 | 127 | 14.6 |
| 3不知道 | 20 | 2.3 |
| miss | 103 |  |

**G4 是否接受了卡介苗**？

|  | Freq | Percent |
| --- | --- | --- |
| 1是 | 725 | 83.1 |
| 2否 | 62 | 7.6 |
| miss | 160 |  |

**G4.1 共接中了几次百白破疫苗**？

|  | G4.1接种百白破疫苗次数 |
| --- | --- |
| Mean | 2.56 |
| Median | 3 |
| % of G4.1=0 | 105（13.5%） |
| miss | 203 |

**G4.2 共服了几次小儿麻痹糖丸？**

|  | G4.2服用小儿麻痹糖丸次数 |
| --- | --- |
| Mean | 2.72 |
| Median | 3 |
| % of G4.2=0 | 82（10.3%） |
| miss | 185 |

**G4.3 是否接种了麻疹疫苗？**

|  | Freq | Percent（%） |
| --- | --- | --- |
| 1是 | 606 | 75.1 |
| 2否 | 201 | 24.9 |
| miss | 167 |  |

**G4.4 共接种了几次乙肝疫苗？**

|  | G4.4 接种乙肝疫苗次数 |
| --- | --- |
| Mean | 2.57 |
| Median | 3 |
| % of G4.2=0 | 46（5.6%） |
| miss | 166 |

**G4.4.1第一针乙肝疫苗的接种时间?**

If G4.4>0

|  | Freq | Percent |
| --- | --- | --- |
| 1出生后24小时内 | 544 | 66 |
| 2 出生后1周内 | 39 | 4.7 |
| 3 出生后1个月内 | 66 | 8 |
| 4 1个月以上 | 97 | 11.8 |
| 5 不清楚 | 78 | 9.5 |
| miss | 150 |  |

**G5 通常在哪里进行免疫接种的?**

|  | Freq | Percent |
| --- | --- | --- |
| 1 疾控中心 | 11 | 1.3 |
| 2 卫生院 | 151 | 18 |
| 3 社区卫生服务中心 | 17 | 2 |
| 4 卫生室/所/站 | 585 | 69.6 |
| 5 其它 | 76 | 9 |
| miss | 134 |  |

**G6 这个孩子近2周内是否有过腹泻（拉肚子）**?

|  | Freq | Percent |
| --- | --- | --- |
| 1是 | 123 | 14.5 |
| 2否 | 728 | 85.5 |
| miss | 123 |  |

**G7 腹泻期间是否吃过？**

If G6=1

|  | Freq | Percent |
| --- | --- | --- |
| 1 口服补液盐粉剂 | 83 | 85.6 |
| 2 口服补液盐溶液 | 9 | 9.3 |
| 3 家庭自制口服补液盐溶液 | 5 | 5.2 |
| miss | 26 |  |

1. **TB knowledge（肺结核知识知晓情况）**

For TB and Hypertension, only keep the cases who answered the questions (like 户主)。 这一部分我们只需分析回答问题的记录。调查表上原来有被调查成员编码，但是现在数据库中没有这个变量。能否请宁夏医学院的老师核查以下问卷，增加这个变量。否则我们无法与回答者的教育程度等其他变量相对应。

数据库中已加入这个变量，不同文化程度的表格都做了分析

因为暂时还无法与教育程度相对应，所以目前先只分析合计，以及与收入水平的交叉表。

- 1. By education：（按文化程度）

Education: A7 文化程度：高中及以上文化程度的比例很小，可合并为一个分类。

- 1. By income：Income Level（按收入水平）
  2. By education：（按文化程度）

Education: A7 文化程度：高中及以上文化程度的比例很小，可合并为一个分类。

- 1. By income：Income Level（按收入水平）

**I2.1 您认为肺结核是一种严重的疾病吗？**

Column percent

|  | 1没上过学 | 2小学 | 3 初中 | 4 高中以上 | 合计 |
| --- | --- | --- | --- | --- | --- |
|  | Education 1 | Education 2 | Education 3 | Education 4 | Total |
| 1 非常严重 | 42.9% | 54.2% | 55.7% | 67.8% | 50.8% |
| 2有些严重 | 14.4% | 11.7% | 21.1% | 13.6% | 14.8% |
| 3不是很严重 | 3.7% | 3.4% | 4.1% | 5.1% | 3.7% |
| 4 不知道 | 38.9% | 30.6% | 19.1% | 13.6% | 30.7% |

收入将1%最大值设为缺失值（分析时剔除24户，共分析2484户）

|  | Income 1 | Income 2 | Income 3 | Income 4 | Income 5 |
| --- | --- | --- | --- | --- | --- |
| 1 非常严重 | 47.9% | 46.5% | 53.0% | 53.9% | 52.9% |
| 2有些严重 | 13.1% | 17.2% | 14.3% | 12.7% | 17.2% |
| 3不是很严重 | 2.5% | 3.7% | 3.5% | 5.0% | 4.6% |
| 4 不知道 | 36.5% | 32.5% | 29.2% | 28.4% | 25.3% |

I2.2 **您是从哪种途径知道肺结核这种病的？**

By Education:

|  | 1没上过学 | 2小学 | 3 初中 | 4 高中以上 | 合计 |
| --- | --- | --- | --- | --- | --- |
|  | Education 1 | Education 2 | Education 3 | Education 4 | Total |
| 1报刊 | 2.1% | 2.3% | 4.4% | 8.5% | 3.0% |
| 2广播 | 2.6% | 2.4% | 4.3% | 6.8% | 3.1% |
| 3电视 | 16.8% | 21.0% | 27.3% | 36.4% | 21.4% |
| 4公告栏 | 3.6% | 2.9% | 7.0% | 7.6% | 4.2% |
| 5宣传手册等 | 8.5% | 11.4% | 22.4% | 24.6% | 13.2% |
| 6医务人员 | 16.3% | 15.5% | 18.4% | 26.3% | 16.9% |
| 7家人朋友等 | 40.7% | 41.6% | 48.2% | 40.7% | 42.5% |
| 8宗教领袖 | 0.7% | 0.4% | .0% | .0% | 0.4% |
| 9教师 | 1.2% | .3% | 1.5% | .8% | 1.0% |
| 10其他 | 1.1% | 2.0% | 1.7% | .8% | 1.6% |
| 11不知道 | 37.5% | 30.9% | 20.1% | 11.0% | 30.3% |

By income:

收入将1%最大值设为缺失值（分析时剔除24户，共分析2484户）

|  | Income 1 | Income 2 | Income 3 | Income 4 | Income 5 |
| --- | --- | --- | --- | --- | --- |
| 1报刊 | 0.8% | 2.4% | 3.3% | 4.1% | 4.6% |
| 2广播 | 2.4% | 3.7% | 1.7% | 2.8% | 4.8% |
| 3电视 | 16.8% | 20.5% | 21.5% | 25.2% | 24.6% |
| 4公告栏 | 2.5% | 4.7% | 3.3% | 5.6% | 5.5% |
| 5宣传手册等 | 8.6% | 12.2% | 14.3% | 14.9% | 17.0% |
| 6医务人员 | 17.0% | 17.0% | 16.8% | 16.4% | 17.7% |
| 7家人朋友等 | 40.0% | 39.3% | 45.8% | 41.8% | 46.2% |
| 8宗教领袖 | 0.7% | 0.6% | 0.2% | 0.4% | 0.2% |
| 9教师 | 0.0% | 0.8% | 0.6% | 1.9% | 1.8% |
| 10其他 | .8% | 2.0% | 1.0% | 2.2% | 2.1% |
| 11不知道 | 34.1% | 32.7% | 28.2% | 28.4% | 27.1% |

**I2.3 肺结核有哪些症状？**

By Education:

|  | 1没上过学 | 2小学 | 3 初中 | 4 高中以上 | 合计 |
| --- | --- | --- | --- | --- | --- |
|  | Education 1 | Education 2 | Education 3 | Education 4 | Total |
| 1 皮疹 | 0.8% | 1.2% | 0.6% | 5.1% | 1.1% |
| 2 咳嗽 | 28.3% | 30.4% | 42.0% | 54.2% | 33.1% |
| 3 咳嗽3周以上 | 9.2% | 11.1% | 14.7% | 25.4% | 11.8% |
| 4 咳血 | 10.4% | 10.0% | 11.2% | 25.4% | 11.1% |
| 5 严重头痛 | 3.8% | 3.2% | 3.3% | 9.3% | 3.7% |
| 6 恶心 | 4.4% | 5.9% | 6.4% | 9.3% | 5.6% |
| 7 消瘦 | 7.9% | 5.9% | 7.9% | 18.6% | 7.7% |
| 8 发热 | 6.2% | 7.0% | 7.2% | 18.6% | 7.3% |
| 9 不明原因发热7天以上 | 1.1% | 1.5% | .8% | 3.4% | 1.3% |
| 10 胸痛 | 9.5% | 10.4% | 10.3% | 16.9% | 10.3% |
| 11 气短 | 17.3% | 18.1% | 20.5% | 27.1% | 18.7% |
| 12 持续乏力 | 9.1% | 9.4% | 13.0% | 16.1% | 10.3% |
| 13 其它 | 0.6% | 0.9% | 0.6% | 0.0% | 0.7% |
| 14 不知道 | 61.3% | 58.0% | 46.4% | 28.8% | 55.5% |

By income:

收入将1%最大值设为缺失值（分析时剔除24户，共分析2484户）

|  | Income 1 | Income 2 | Income 3 | Income 4 | Income 5 |
| --- | --- | --- | --- | --- | --- |
| 1 皮疹 | 1.0% | 0.6% | 1.2% | 1.3% | 1.6% |
| 2 咳嗽 | 31.8% | 31.0% | 31.3% | 35.3% | 36.6% |
| 3 咳嗽3周以上 | 7.9% | 10.8% | 9.9% | 14.4% | 16.3% |
| 4 咳血 | 9.2% | 9.7% | 11.0% | 12.1% | 14.3% |
| 5 严重头痛 | 1.8% | 3.7% | 3.3% | 4.5% | 5.7% |
| 6 恶心 | 3.0% | 4.3% | 5.0% | 6.9% | 9.2% |
| 7 消瘦 | 4.0% | 4.9% | 9.1% | 8.2% | 13.3% |
| 8 发热 | 4.2% | 5.3% | 6.8% | 9.9% | 10.6% |
| 9 不明原因发热7天以上 | .8% | 1.2% | .4% | 1.5% | 2.5% |
| 10 胸痛 | 6.1% | 9.1% | 8.9% | 11.6% | 17.5% |
| 11 气短 | 15.3% | 18.5% | 16.6% | 21.1% | 23.0% |
| 12 持续乏力 | 7.4% | 8.1% | 9.5% | 11.2% | 16.3% |
| 13 其它 | .2% | 1.2% | .4% | .6% | 1.1% |
| 14 不知道 | 60.3% | 57.4% | 56.3% | 51.9% | 49.9% |

**I2.4 通过何种途径可以感染肺结核？**

By education:

|  | 1没上过学 | 2小学 | 3 初中 | 4 高中以上 | 合计 |
| --- | --- | --- | --- | --- | --- |
|  | Education 1 | Education 2 | Education 3 | Education 4 | Total |
| 1 握手 | 1.9% | 1.9% | 3.5% | 6.8% | 2.4% |
| 2 结核病人咳嗽或打喷嚏喷出的飞沫 | 19.4% | 25.0% | 34.0% | 50.8% | 25.9% |
| 3 共同进餐 | 15.2% | 17.5% | 27.3% | 30.5% | 19.2% |
| 4 公用餐具 | 9.9% | 12.5% | 20.7% | 28.0% | 13.9% |
| 5 触摸公共场所物品 | 3.1% | 2.1% | 3.1% | 5.1% | 2.8% |
| 6 其它 | 1.7% | 1.7% | 1.5% | 3.4% | 1.7% |
| 7 不知道 | 73.5% | 68.4% | 55.1% | 37.3% | 66.1% |

By income:

收入将1%最大值设为缺失值（分析时剔除24户，共分析2484户）

|  | Income 1 | Income 2 | Income 3 | Income 4 | Income 5 |
| --- | --- | --- | --- | --- | --- |
| 1 握手 | 1.7% | 2.0% | 1.9% | 3.4% | 3.0% |
| 2 结核病人咳嗽或打喷嚏喷出的飞沫 | 20.0% | 22.5% | 24.2% | 32.8% | 32.2% |
| 3 共同进餐 | 13.8% | 16.8% | 19.3% | 24.1% | 23.9% |
| 4 公用餐具 | 9.7% | 11.8% | 13.0% | 15.5% | 21.1% |
| 5 触摸公共场所物品 | 1.7% | 1.4% | 1.9% | 5.0% | 4.8% |
| 6 其它 | 0.5% | 2.2% | 1.7% | 2.4% | 2.3% |
| 7 不知道 | 71.8% | 69.8% | 68.7% | 60.8% | 57.5% |

**I2.5 如何预防肺结核？**

By Education:

|  | 1没上过学 | 2小学 | 3 初中 | 4 高中以上 | 合计 |
| --- | --- | --- | --- | --- | --- |
|  | Education 1 | Education 2 | Education 3 | Education 4 | Total |
| 1 避免握手 | 2.5% | 2.9% | 5.6% | 7.6% | 3.5% |
| 2 在咳嗽或打喷嚏时遮掩口鼻 | 15.6% | 18.4% | 23.6% | 39.8% | 19.4% |
| 3 避免与人一起进餐 | 15.0% | 19.2% | 26.5% | 33.1% | 19.7% |
| 4 在公共场所触摸物品后洗手 | 4.9% | 6.2% | 8.7% | 18.6% | 6.8% |
| 5 家中关窗 | 2.1% | 1.0% | 1.7% | 2.5% | 1.6% |
| 6 良好的营养 | 3.3% | 3.5% | 4.1% | 6.8% | 3.7% |
| 7 祈祷 | 1.8% | 1.0% | 1.5% | .8% | 1.4% |
| 8 不知道 | 75.5% | 71.7% | 59.2% | 50.8% | 69.6% |
| 9 其它 | 1.1% | 1.3% | 2.7% | 1.7% | 1.6% |

By income:

收入将1%最大值设为缺失值（分析时剔除24户，共分析2484户）

|  | Income 1 | Income 2 | Income 3 | Income 4 | Income 5 |
| --- | --- | --- | --- | --- | --- |
| 1 避免握手 | 2.2% | 3.0% | 2.5% | 5.4% | 4.6% |
| 2 在咳嗽或打喷嚏时遮掩口鼻 | 12.6% | 19.3% | 17.2% | 24.6% | 24.6% |
| 3 避免与人一起进餐 | 13.1% | 16.8% | 20.5% | 24.6% | 25.7% |
| 4 在公共场所触摸物品后洗手 | 4.4% | 4.9% | 6.0% | 9.3% | 10.1% |
| 5 家中关窗 | 0.8% | 0.8% | 1.0% | 3.2% | 2.5% |
| 6 良好的营养 | 1.8% | 3.0% | 2.5% | 5.8% | 6.2% |
| 7 祈祷 | 1.2% | 1.4% | 1.0% | 2.2% | 1.4% |
| 8 不知道 | 77.3% | 73.0% | 71.0% | 63.1% | 61.4% |
| 9 其它 | 1.5% | .8% | 1.4% | 1.5% | 2.8% |

**I2.6 您认为谁更容易感染肺结核？**

By Education:

|  | 1没上过学 | 2小学 | 3 初中 | 4 高中以上 | 合计 |
| --- | --- | --- | --- | --- | --- |
|  | Education 1 | Education 2 | Education 3 | Education 4 | Total |
| 1 任何人 | 32.8% | 29.2% | 33.7% | 39.8% | 32.0% |
| 2 只有穷人 | 3.5% | 3.9% | 3.9% | 5.9% | 3.8% |
| 3 只有无家可归者 | 2.0% | 2.0% | 3.1% | 2.5% | 2.2% |
| 4 只有酗酒者 | 3.5% | 4.6% | 7.9% | 5.1% | 4.9% |
| 5 只有吸毒者 | 5.0% | 6.4% | 8.7% | 6.8% | 6.3% |
| 6 只有艾滋病病毒携带者 | 2.3% | 2.5% | 2.5% | 5.9% | 2.6% |
| 7 只有坐牢者 | 0.8% | 0.8% | 0.2% | 0.8% | 0.7% |
| 8 其它 | 46.5% | 45.4% | 38.7% | 35.6% | 44.0% |

By income:

收入将1%最大值设为缺失值（分析时剔除24户，共分析2484户）

|  | Income 1 | Income 2 | Income 3 | Income 4 | Income 5 |
| --- | --- | --- | --- | --- | --- |
| 1 任何人 | 29.2% | 32.0% | 29.8% | 32.8% | 36.8% |
| 2 只有穷人 | 3.5% | 4.5% | 2.7% | 3.4% | 4.6% |
| 3 只有无家可归者 | 2.0% | 2.2% | 0.6% | 3.4% | 2.8% |
| 4 只有酗酒者 | 4.0% | 5.5% | 4.8% | 6.0% | 4.4% |
| 5 只有吸毒者 | 3.2% | 7.5% | 6.8% | 9.9% | 4.6% |
| 6 只有艾滋病病毒携带者 | 0.8% | 2.6% | 1.7% | 4.7% | 3.7% |
| 7 只有坐牢者 | 0.3% | 1.2% | 0.2% | 1.3% | 0.5% |
| 8 其它 | 47.2% | 43.2% | 45.5% | 43.1% | 41.1% |

**I2.7 肺结核能治愈吗？**

By Education:

|  | 1没上过学 | 2小学 | 3 初中 | 4 高中以上 | 合计 |
| --- | --- | --- | --- | --- | --- |
|  | Education 1 | Education 2 | Education 3 | Education 4 | Total |
| 1能 | 59.6% | 59.2% | 69.6% | 79.7% | 62.5% |
| 2 不能 | 31.0% | 30.3% | 24.6% | 16.9% | 28.7% |
| 999（不知道） | 9.4% | 10.5% | 5.8% | 3.4% | 8.8% |

By income:

收入将1%最大值设为缺失值（分析时剔除24户，共分析2484户）

|  | Income 1 | Income 2 | Income 3 | Income 4 | Income 5 |
| --- | --- | --- | --- | --- | --- |
| 1能 | 61.5% | 60.9% | 59.0% | 64.4% | 67.6% |
| 2 不能 | 32.1% | 31.0% | 31.1% | 25.4% | 22.3% |
| 999（不知道） | 6.4% | 8.1% | 9.9% | 10.1% | 10.1% |

**I2.8 肺结核患者应如何治疗？**

By Education:

|  | 1没上过学 | 2小学 | 3 初中 | 4 高中以上 | 合计 |
| --- | --- | --- | --- | --- | --- |
|  | Education 1 | Education 2 | Education 3 | Education 4 | Total |
| 1 中草药 | 10.6% | 9.7% | 8.9% | 14.4% | 10.1% |
| 2 在家休息，无需治疗 | 2.9% | 1.8% | 1.4% | 0.8% | 2.1% |
| 3 祈祷 | 1.8% | 1.5% | 0.6% | 0.8% | 1.4% |
| 4 医疗机构给的特定药物 | 36.3% | 38.7% | 47.8% | 65.3% | 40.9% |
| 5 DOTS策略 | 3.1% | 3.4% | 4.1% | 5.1% | 3.5% |
| 6 不知道 | 52.6% | 51.3% | 43.3% | 26.3% | 49.0% |
| 7 其它 | 2.1% | 2.2% | 2.9% | 4.2% | 2.4% |

By income:

收入将1%最大值设为缺失值（分析时剔除24户，共分析2484户）

|  | Income 1 | Income 2 | Income 3 | Income 4 | Income 5 |
| --- | --- | --- | --- | --- | --- |
| 1 中草药 | 7.4% | 8.3% | 8.5% | 12.7% | 14.5% |
| 2 在家休息，无需治疗 | 2.2% | 2.6% | 1.7% | 2.2% | 1.6% |
| 3 祈祷 | 1.5% | 1.8% | 1.0% | 1.3% | 1.4% |
| 4 医疗机构给的特定药物 | 38.0% | 35.1% | 41.6% | 42.7% | 47.6% |
| 5 DOTS策略 | 1.3% | 2.6% | 4.3% | 6.0% | 3.9% |
| 6 不知道 | 53.4% | 53.8% | 49.3% | 44.4% | 42.8% |
| 7 其它 | 2.9% | 2.4% | 1.7% | 3.0% | 1.8% |

**I2.9 如果您认为自己得了肺结核，您会去治疗吗？**

By Education:

|  | 1没上过学 | 2小学 | 3 初中 | 4 高中以上 | 合计 |
| --- | --- | --- | --- | --- | --- |
|  | Education 1 | Education 2 | Education 3 | Education 4 | Total |
| 1会 | 93.6% | 95.4% | 98.1% | 99.2% | 95.4% |
| 2 不会 | 6.4% | 4.6% | 1.9% | .8% | 4.%6 |

By income:

收入将1%最大值设为缺失值（分析时剔除24户，共分析2484户）

|  | Income 1 | Income 2 | Income 3 | Income 4 | Income 5 |
| --- | --- | --- | --- | --- | --- |
| 1会 | 93.1% | 96.1% | 95.7% | 96.8% | 95.9% |
| 2 不会 | 6.9% | 3.9% | 4.3% | 3.2% | 4.1% |

**I2.9.1 如果治疗，您会选择去哪里治？**

If I2.9=1

By Education:

|  | 1没上过学 | 2小学 | 3 初中 | 4 高中以上 | 合计 |
| --- | --- | --- | --- | --- | --- |
|  | Education 1 | Education 2 | Education 3 | Education 4 | Total |
| 1 去村卫生室 | 4.3% | 3.8% | 2.4% | 4.3% | 3.7% |
| 2 去乡镇卫生院 | 12.7% | 11.0% | 7.9% | 10.3% | 10.9% |
| 3 去县级以上医院 | 75.0% | 76.1% | 77.5% | 70.1% | 75.7% |
| 4 去结核病防治所 | 16.3% | 17.6% | 22.5% | 30.8% | 18.8% |
| 5 去药店买药 | 1.9% | 1.7% | 2.2% | .9% | 1.8% |
| 6 传统治疗（如看中医） | 1.2% | 1.5% | 1.6% | .9% | 1.4% |
| 7 自我治疗（如草药等） | 0.6% | 0.8% | 0.6% | 0.0% | 0.6% |
| 8 其它 | 1.8% | 1.5% | 1.8% | 1.7% | 1.7% |

By income:

收入将1%最大值设为缺失值（分析时剔除24户，共分析2484户）

|  | Income 1 | Income 2 | Income 3 | Income 4 | Income 5 |
| --- | --- | --- | --- | --- | --- |
| 1 去村卫生室 | 3.4% | 3.5% | 4.8% | 2.9% | 4.1% |
| 2 去乡镇卫生院 | 9.9% | 11.5% | 13.0% | 10.5% | 9.4% |
| 3 去县级以上医院 | 76.7% | 78.4% | 74.7% | 74.2% | 74.3% |
| 4 去结核病防治所 | 17.3% | 16.2% | 16.7% | 21.8% | 22.5% |
| 5 去药店买药 | 1.4% | 1.6% | 1.3% | 2.2% | 2.9% |
| 6 传统治疗（如看中医） | 1.3% | .2% | 1.9% | 1.6% | 1.9% |
| 7 自我治疗（如草药等） | 0.5% | 0.4% | 0.6% | 0.7% | 0.7% |
| 8 其它 | 1.6% | 1.6% | 1.9% | 1.6% | 1.7% |

**I2.9.2 您不去医疗机构原因是什么？**

If I2.9=2

By Education:

|  | 1没上过学 | 2小学 | 3 初中 | 4 高中以上 | 合计 |
| --- | --- | --- | --- | --- | --- |
|  | Education 1 | Education 2 | Education 3 | Education 4 | Total |
| 1 不知道去哪里看病 | 3.2% | 4.8% | 0% | 0% | 3.5% |
| 2 费用太高 | 53.2% | 52.4% | 40.0% | 100.0% | 52.2% |
| 3 交通不便/距离太远 | 16.1% | 19.0% | 50.0% | .0% | 20.0% |
| 4 不信任医务人员 | 1.6% | 2.4% | 10.0% | .0% | 2.6% |
| 5 医务人员态度差 | 0% | 0% | 0% | 0% | 0% |
| 6 工作离不开 | 0% | 0% | 0% | 0% | 0% |
| 7 不想知道坏消息 | 0% | 2.4% | .0% | 0% | 0.9% |
| 8 其它 | 24.2% | 9.5% | 10.0% | 0% | 17.4% |

By income:

收入将1%最大值设为缺失值（分析时剔除24户，共分析2484户）

|  | Income 1 | Income 2 | Income 3 | Income 4 | Income 5 |
| --- | --- | --- | --- | --- | --- |
| 1 不知道去哪里看病 | 0% | 5.0% | 0% | 13.3% | 5.6% |
| 2 费用太高 | 61.0% | 60.0% | 52.4% | 40.0% | 33.3% |
| 3 交通不便/距离太远 | 14.6% | 10.0% | 19.0% | 40.0% | 27.8% |
| 4 不信任医务人员 | 0% | 0% | 4.8% | 6.7% | 5.6% |
| 5 医务人员态度差 | 0% | 0% | 0% | 0% | 0% |
| 6 工作离不开 | 0% | 0% | 0% | 0% | 0% |
| 7 不想知道坏消息 | 2.4% | .0% | .0% | .0% | .0% |
| 8 其它 | 22.0% | 15.0% | 9.5% | 13.3% | 22.2% |

**I2.10 您认为肺结核诊断和治疗费用怎么样？**

By Education:

|  | 1没上过学 | 2小学 | 3 初中 | 4 高中以上 | 合计 |
| --- | --- | --- | --- | --- | --- |
|  | Education 1 | Education 2 | Education 3 | Education 4 | Total |
| 1 应该免费 | 29.9% | 33.6% | 41.4% | 45.8% | 34.4% |
| 2 费用合理 | 3.2% | 6.4% | 8.9% | 14.4% | 6.1% |
| 3 费用有点高 | 9.1% | 8.6% | 8.5% | 4.2% | 8.6% |
| 4 费用非常高 | 10.9% | 10.1% | 8.3% | 9.3% | 10.0% |
| 5 不知道 | 46.9% | 41.3% | 32.9% | 26.3% | 41.0% |

By income:

收入将1%最大值设为缺失值（分析时剔除24户，共分析2484户）

|  | Income 1 | Income 2 | Income 3 | Income 4 | Income 5 |
| --- | --- | --- | --- | --- | --- |
| 1 应该免费 | 30.3% | 33.3% | 33.7% | 40.5% | 35.2% |
| 2 费用合理 | 5.2% | 5.7% | 6.0% | 4.7% | 9.2% |
| 3 费用有点高 | 9.7% | 7.9% | 8.5% | 8.0% | 8.3% |
| 4 费用非常高 | 10.6% | 8.9% | 9.5% | 11.4% | 9.4% |
| 5 不知道 | 44.2% | 44.2% | 42.2% | 35.3% | 37.9% |

**I2.11您希望通过哪些途径来获取肺结核的防治知识和信息？**

By Education:

|  | 1没上过学 | 2小学 | 3 初中 | 4 高中以上 | 合计 |
| --- | --- | --- | --- | --- | --- |
|  | Education 1 | Education 2 | Education 3 | Education 4 | Total |
| 1报刊 | 3.1% | 3.8% | 6.0% | 6.8% | 4.15 |
| 2广播 | 5.9% | 6.4% | 8.3% | 11.0% | 6.8% |
| 3电视 | 39.5% | 42.4% | 45.1% | 44.9% | 41.9% |
| 4公告栏 | 4.7% | 4.4% | 8.3% | 6.8% | 5.5% |
| 5宣传手册等 | 23.9% | 23.7% | 34.6% | 36.4% | 26.6% |
| 6医务人员 | 47.0% | 40.4% | 41.2% | 45.8% | 43.3% |
| 7家人朋友等 | 33.2% | 28.9% | 28.6% | 23.7% | 30.3% |
| 8宗教领袖 | 1.4% | 1.1% | .6% | .0% | 1.1% |
| 9教师 | 2.4% | 1.9% | 1.9% | 4.2% | 2.2% |
| 10其他 | 8.4% | 6.2% | 3.7% | 4.2% | 6.4% |

By income:

收入将1%最大值设为缺失值（分析时剔除24户，共分析2484户）

|  | Income 1 | Income 2 | Income 3 | Income 4 | Income 5 |
| --- | --- | --- | --- | --- | --- |
| 1报刊 | 2.5% | 4.1% | 4.3% | 3.9% | 5.7% |
| 2广播 | 6.6% | 5.9% | 5.6% | 7.3% | 8.5% |
| 3电视 | 36.5% | 42.8% | 45.5% | 46.3% | 39.8% |
| 4公告栏 | 4.4% | 5.1% | 5.8% | 5.2% | 7.1% |
| 5宣传手册等 | 17.8% | 24.1% | 28.0% | 34.1% | 32.4% |
| 6医务人员 | 43.7% | 41.0% | 41.0% | 42.7% | 48.5% |
| 7家人朋友等 | 29.2% | 33.1% | 29.8% | 31.0% | 26.9% |
| 8宗教领袖 | 1.5% | .8% | 1.4% | .6% | .9% |
| 9教师 | 1.2% | .8% | 1.7% | 2.8% | 5.1% |
| 10其他 | 8.2% | 6.9% | 5.8% | 5.2% | 5.5% |

1. **Hypertension knowledge（高血压知识知晓情况）**
   1. By education
   2. By income

**I3.1 高血压发病与饮食有关吗？**

Column percent

|  | Education 1 | Education 2 | Education 3 | Education 4 | Total |
| --- | --- | --- | --- | --- | --- |
| 1 有 | 33.3% | 36.8% | 50.7% | 67.8% | 39.8% |
| 2 没有 | 12.5% | 14.8% | 12.6% | 8.5% | 13.2% |
| 3 不知道 | 54.2% | 48.3% | 36.8% | 23.7% | 47.0% |

收入将1%最大值设为缺失值（分析时剔除24户，共分析2484户）

| Income | Income 1 | Income 2 | Income 3 | Income 4 | Income 5 |
| --- | --- | --- | --- | --- | --- |
| 1 有 | 30.6% | 36.9% | 39.1% | 48.1% | 46.7% |
| 2 没有 | 12.6% | 12.6% | 14.9% | 13.1% | 12.9% |
| 3 不知道 | 56.8% | 50.5% | 46.0% | 38.8% | 40.5% |

**I3.1.1 吃盐多少对高血压发病有影响吗？**

|  | Education 1 | Education 2 | Education 3 | Education 4 | Total |
| --- | --- | --- | --- | --- | --- |
| 1 有 | 34.0% | 36.7% | 51.3% | 61.0% | 39.8% |
| 2 没有 | 8.7% | 11.1% | 8.1% | 5.1% | 9.3% |
| 3 不知道 | 57.4% | 52.2% | 40.6% | 33.9% | 51.0% |

收入将1%最大值设为缺失值（分析时剔除24户，共分析2484户）

| Income | Income 1 | Income 2 | Income 3 | Income 4 | Income 5 |
| --- | --- | --- | --- | --- | --- |
| 1 有 | 32.3% | 34.1% | 41.0% | 47.2% | 46.4% |
| 2 没有 | 7.6% | 11.4% | 11.0% | 8.0% | 8.7% |
| 3 不知道 | 60.2% | 54.4% | 48.0% | 44.8% | 44.8% |

**I3.2高血压发病与吸烟有关吗？**

|  | Education 1 | Education 2 | Education 3 | Education 4 | Total |
| --- | --- | --- | --- | --- | --- |
| 1 有 | 33.7% | 38.3% | 53.2% | 69.5% | 41.1% |
| 2 没有 | 7.0% | 7.4% | 7.2% | 4.2% | 7.1% |
| 3 不知道 | 59.2% | 54.3% | 39.7% | 26.3% | 51.9% |

收入将1%最大值设为缺失值（分析时剔除24户，共分析2484户）

| Income | Income 1 | Income 2 | Income 3 | Income 4 | Income 5 |
| --- | --- | --- | --- | --- | --- |
| 1 有 | 33.9% | 39.3% | 40.4% | 47.0% | 46.7% |
| 2 没有 | 5.4% | 6.3% | 7.9% | 9.7% | 6.7% |
| 3 不知道 | 60.7% | 54.4% | 51.8% | 43.3% | 46.7% |

**I3.3高血压得病与肥胖有关吗？**

|  | Education 1 | Education 2 | Education 3 | Education 4 | Total |
| --- | --- | --- | --- | --- | --- |
| 1 有 | 39.7% | 46.1% | 59.4% | 75.4% | 47.8% |
| 2 没有 | 6.7% | 7.1% | 6.2% | 3.4% | 6.6% |
| 3 不知道 | 53.6% | 46.8% | 34.4% | 21.2% | 45.7% |

收入将1%最大值设为缺失值（分析时剔除24户，共分析2484户）

| Income | Income 1 | Income 2 | Income 3 | Income 4 | Income 5 |
| --- | --- | --- | --- | --- | --- |
| 1 有 | 36.5% | 44.2% | 47.4% | 57.3% | 57.0% |
| 2 没有 | 7.1% | 6.3% | 8.7% | 3.7% | 6.9% |
| 3 不知道 | 56.5% | 49.5% | 43.9% | 39.0% | 36.1% |

**I3.4高血压得病与饮酒有关吗？**

|  | Education 1 | Education 2 | Education 3 | Education 4 | Total |
| --- | --- | --- | --- | --- | --- |
| 1 有 | 37.3% | 39.8% | 58.2% | 72.9% | 44.2% |
| 2 没有 | 3.8% | 5.1% | 4.4% | 1.7% | 4.3% |
| 3 不知道 | 58.9% | 55.1% | 37.3% | 25.4% | 51.5% |

收入将1%最大值设为缺失值（分析时剔除24户，共分析2484户）

| Income | Income 1 | Income 2 | Income 3 | Income 4 | Income 5 |
| --- | --- | --- | --- | --- | --- |
| 1 有 | 35.5% | 41.8% | 43.1% | 53.4% | 49.9% |
| 2 没有 | 4.0% | 4.7% | 5.2% | 3.4% | 3.9% |
| 3 不知道 | 60.5% | 53.5% | 51.8% | 43.1% | 46.2% |

**I3.5 一个高血压病人，如果血压得不到有效控制，可发展为哪些疾病？**

|  | Education 1 | Education 2 | Education 3 | Education 4 | Total |
| --- | --- | --- | --- | --- | --- |
| 1中风 | 20.4% | 23.3% | 32.3% | 44.9% | 25.1% |
| 2冠心病 | 9.5% | 12.2% | 20.3% | 33.9% | 13.8% |
| 3肿瘤 | 3.6% | 4.5% | 4.8% | 6.8% | 4.3% |
| 4不会发展 | 0.5% | 0.8% | 0.8% | 0% | 0.6% |
| 99不知道 | 74.3% | 70.9% | 58.2% | 44.9% | 68.4% |

收入将1%最大值设为缺失值（分析时剔除24户，共分析2484户）

| Income | Income 1 | Income 2 | Income 3 | Income 4 | Income 5 |
| --- | --- | --- | --- | --- | --- |
| 1中风 | 17.5% | 21.1% | 25.5% | 30.8% | 32.4% |
| 2冠心病 | 7.9% | 9.9% | 13.9% | 17.2% | 21.8% |
| 3肿瘤 | 2.5% | 3.0% | 3.9% | 5.2% | 7.1% |
| 4不会发展 | 0.3% | 0.4% | 0.6% | 0.4% | 1.1% |
| 99不知道 | 78.7% | 73.8% | 67.7% | 60.3% | 57.9% |

**I3.6 一个高血压病人，应如何来控制血压？**

|  | Education 1 | Education 2 | Education 3 | Education 4 | Total |
| --- | --- | --- | --- | --- | --- |
| 1按医嘱服药 | 27.3% | 30.2% | 41.4% | 48.3% | 32.3% |
| 2按医生的建议调理饮食控制食盐 | 19.1% | 23.0% | 29.8% | 44.9% | 23.9% |
| 3少吃含脂肪和胆固醇的肉蛋类 | 16.8% | 18.4% | 26.7% | 39.0% | 20.5% |
| 4保持情绪稳定 | 9.3% | 12.8% | 12.4% | 22.9% | 11.8% |
| 5做适宜运动 | 6.7% | 10.0% | 11.6% | 25.4% | 9.8% |
| 6控制体重 | 7.4% | 8.4% | 9.9% | 21.2% | 8.9% |
| 7不用控制 | 0.7% | 0.7% | 0.6% | 0.8% | 0.7% |
| 99不知道 | 67.3% | 63.9% | 50.1% | 34.7% | 61.0% |

收入将1%最大值设为缺失值（分析时剔除24户，共分析2484户）

| Income | Income 1 | Income 2 | Income 3 | Income 4 | Income 5 |
| --- | --- | --- | --- | --- | --- |
| 1按医嘱服药 | 27.2% | 28.2% | 32.7% | 37.1% | 37.5% |
| 2按医生的建议调理饮食控制食盐 | 15.5% | 20.3% | 23.2% | 30.2% | 32.6% |
| 3少吃含脂肪和胆固醇的肉蛋类 | 12.6% | 17.0% | 19.5% | 27.4% | 28.0% |
| 4保持情绪稳定 | 6.9% | 9.7% | 10.1% | 14.7% | 19.1% |
| 5做适宜运动 | 4.4% | 5.9% | 8.7% | 13.8% | 17.7% |
| 6控制体重 | 3.9% | 6.7% | 7.5% | 11.6% | 16.3% |
| 7不用控制 | 0.3% | 0.8% | 0.2% | 0.2% | 1.6% |
| 99不知道 | 69.6% | 67.9% | 60.2% | 53.4% | 50.6% |

1. **Smoking （吸烟）**
   1. By education
   2. By income

**I1.1 您吸烟吗？**

**吸烟部分我们调查时统一了15岁以上在家人必须回答，但家人如果知情也可以代为回答该问题！I1.2、I1.3和 I1.4有部分缺失值，知情人记不清没有回答该问题！**

|  | Education 1 | Education 2 | Education 3 | Education 4 | Total |
| --- | --- | --- | --- | --- | --- |
| 1 从未吸 | 1836 | 1640 | 881 | 240 | 4597 |
|  | 39.9% | 35.7% | 19.2% | 5.2% | 100.0% |
| 2 偶尔吸 | 37 | 53 | 35 | 12 | 137 |
|  | 27.0% | 38.7% | 25.5% | 8.8% | 100.0% |
| 3 经常吸 | 127 | 231 | 226 | 55 | 639 |
|  | 19.9% | 36.2% | 35.4% | 8.6% | 100.0% |
| 4 已戒烟 | 15 | 23 | 11 | 4 | 53 |
|  | 28.3% | 43.4% | 20.8% | 7.5% | 100.0% |

| Income | Income 1 | Income 2 | Income 3 | Income 4 | Income 5 |
| --- | --- | --- | --- | --- | --- |
| 1 从未吸 | 951 | 883 | 904 | 926 | 933 |
|  | 20.7% | 19.2% | 19.7% | 20.1% | 20.3% |
| 2 偶尔吸 | 34 | 27 | 31 | 23 | 22 |
|  | 24.8% | 19.7% | 22.6% | 16.8% | 16.1% |
| 3 经常吸 | 118 | 125 | 128 | 147 | 121 |
|  | 18.5% | 19.6% | 20.0% | 23.0% | 18.9% |
| 4 已戒烟 | 7 | 12 | 13 | 9 | 12 |
|  | 13.2% | 22.6% | 24.5% | 17.0% | 22.6% |

**I1.2您开始吸烟的年龄多大？**

If I1.1=2|I1.1=3

| Education | Mean Age | Median Age |
| --- | --- | --- |
| 1没上过学 | 21.96 | 20.00 |
| 2小学 | 20.67 | 20.00 |
| 3初中 | 21.23 | 20.00 |
| 4高中及以上 | 22.51 | 22.00 |
| Total | 86.37 | 82 |

| Income | Mean Age | Median Age |
| --- | --- | --- |
| Level 1 | 21.33 | 20.00 |
| Level 2 | 20.97 | 20.00 |
| Level 3 | 21.47 | 20.00 |
| Level 4 | 21.34 | 20.00 |
| Level 5 | 21.39 | 20.00 |

**I1.3 您平均每天吸多少支烟？**

If I1.1=2|I1.1=3

| Education | Mean | Median |
| --- | --- | --- |
| 1没上过学 | 13.18 | 10.00 |
| 2小学 | 14.13 | 15.00 |
| 3初中 | 17.56 | 20.00 |
| 4高中及以上 | 14.43 | 12.50 |
| Total | 59.3 | 57.5 |

| Income | Mean | Median |
| --- | --- | --- |
| Level 1 | 11.90 | 10.00 |
| Level 2 | 13.43 | 15.00 |
| Level 3 | 15.10 | 20.00 |
| Level 4 | 16.93 | 20.00 |
| Level 5 | 18.05 | 20.00 |

**I1.4请问您每月吸烟大约花多少钱？**

If I1.1=2|I1.1=3

| Education | Mean | Median |
| --- | --- | --- |
| 1没上过学 | 71.805 | 40.000 |
| 2小学 | 75.167 | 60.000 |
| 3初中 | 93.196 | 65.000 |
| 4高中及以上 | 105.227 | 75.000 |
| Total | 345.395 | 240 |

| Income | Mean | Median |
| --- | --- | --- |
| Level 1 | 48.583 | 35.000 |
| Level 2 | 74.196 | 60.000 |
| Level 3 | 79.974 | 60.000 |
| Level 4 | 90.693 | 60.000 |
| Level 5 | 114.714 | 90.000 |

**I1.5 请问您戒烟多少年了？**

If I1.1=4

| Education | Mean | Median |
| --- | --- | --- |
| 1没上过学 | 3.67 | 2.00 |
| 2小学 | 6.96 | 3.00 |
| 3初中 | 3.45 | 1.00 |
| 4高中及以上 | 6.25 | 4.50 |
| Total | 20.33 | 10.5 |

| Income | Mean | Median |
| --- | --- | --- |
| Level 1 | 6.57 | 1.00 |
| Level 2 | 4.58 | 2.50 |
| Level 3 | 5.31 | 3.00 |
| Level 4 | 2.78 | 1.00 |
| Level 5 | 19.24 | 7.5 |

**I1.5.1 戒烟的主要原因？**

If I1.1=4

|  | Education 1 | Education 2 | Education 3 | Education 4 | Total |
| --- | --- | --- | --- | --- | --- |
| 1 已患病 | 10 | 13 | 6 | 2 | 31 |
|  | 32.3% | 41.9% | 19.4% | 6.5% | 100.0% |
| 2 预防疾病 | 1 | 4 | 1 | 1 | 7 |
|  | 14.3% | 57.1% | 14.3% | 14.3% | 100.0% |
| 3 经济原因 | 6 | 2 | 3 | 0 | 11 |
|  | 54.5% | 18.2% | 27.3% | .0% | 100.0% |
| 4 家庭反对 | 3 | 3 | 4 | 0 | 10 |
|  | 30.0% | 30.0% | 40.0% | .0% | 100.0% |
| 5 环境限制 | 0 | 0 | 0 | 0 | 0 |
|  | 0 | 0 | 0 | 0 | 0 |
| 6 树立形象 | 0 | 0 | 0 | 0 | 0 |
|  | 0 | 0 | 0 | 0 | 0 |
| 7 经宣传教育 | 0 | 0 | 0 | 0 | 0 |
|  | 0 | 0 | 0 | 0 | 0 |
| 8 经医生劝告 | 1 | 4 | 0 | 0 | 5 |
|  | 20.0% | 80.0% | .0% | .0% | 100.0% |
| 9 其它 | 3 | 3 | 1 | 1 | 8 |
|  | 37.5% | 37.5% | 12.5% | 12.5% | 100.0% |
| 10 不知道 | 0 | 1 | 0 | 0 | 1 |
|  | .0% | 100.0% | .0% | .0% | 100.0% |

| Income | Income 1 | Income 2 | Income 3 | Income 4 | Income 5 |
| --- | --- | --- | --- | --- | --- |
| 1 已患病 | 5 | 9 | 4 | 8 | 5 |
|  | 16.1% | 29.0% | 12.9% | 25.8% | 16.1% |
| 2 预防疾病 | 2 | 2 | 1 | 1 | 1 |
|  | 28.6% | 28.6% | 14.3% | 14.3% | 14.3% |
| 3 经济原因 | 2 | 1 | 5 | 2 | 1 |
|  | 18.2% | 9.1% | 45.5% | 18.2% | 9.1% |
| 4 家庭反对 | 2 | 1 | 5 | 1 | 1 |
|  | 20.0% | 10.0% | 50.0% | 10.0% | 10.0% |
| 5 环境限制 | 0 | 0 | 0 | 0 | 1 |
|  | .0% | .0% | .0% | .0% | 100.0% |
| 6 树立形象 | 7 | 12 | 13 | 9 | 12 |
|  | 13.2% | 22.6% | 24.5% | 17.0% | 22.6% |
| 7 经宣传教育 | 7 | 12 | 13 | 9 | 12 |
|  | 13.2% | 22.6% | 24.5% | 17.0% | 22.6% |
| 8 经医生劝告 | 1 | 2 | 1 | 0 | 1 |
|  | 20.0% | 40.0% | 20.0% | .0% | 20.0% |
| 9 其它 | 1 | 1 | 3 | 0 | 3 |
|  | 12.5% | 12.5% | 37.5% | .0% | 37.5% |
| 10 不知道 | 0 | 0 | 0 | 0 | 1 |
|  | .0% | .0% | .0% | .0% | 100.0% |

**附：**

**疾病分类---编码表**

**编码 疾病名称 编码 疾病名称**

**A . 传染病计**

001. 伤寒和付伤寒

002. 细菌性食物中毒 003. 痢疾

004. 甲型肝炎

005. 其他肠道传染病

006. 结核病

007. 破伤风

008. 败血症

009. 麻疹

010. 流行性乙型脑炎

011. 流行性出血热

012. 乙型肝炎

013. 钩端螺旋体病

014. 非典型肺炎

015. 其他非肠道传染病

**B. 寄生虫病计**

016. 疟疾

017. 血吸虫病

018. 其他寄生虫病

**C. 恶性肿瘤计**

019. 鼻咽恶性肿瘤

020. 食管恶性肿瘤

021. 胃恶性肿瘤

022. 结肠恶性肿瘤

023. 直肠和肛门恶性肿瘤

024. 肝恶性肿瘤

025. 胰恶性肿瘤

026. 气管.支气管和肺恶性肿瘤

027. 乳房恶性肿瘤

028. 子宫颈恶性肿瘤

029. 白血病

030. 其他恶性肿瘤

**D. 良性、原位及动态未定肿瘤计**

031. 子宫良性肿瘤

032. 脑良性肿瘤

033. 其他良性肿瘤

034. 原位肿瘤

035. 动态未定或动态未知的肿瘤计

**E.内分泌.营养和代谢疾病及免疫疾病**

036. 甲状腺功能亢进

037. 糖尿病

038. 营养缺乏或不良

039. 内:佝偻病

040. 肥胖和其他营养过度

041. 其他内、营、代和免疫疾病

**F. 血液和造血器官疾病小计**

042. 贫血

043. 其他血液和造血器官疾病

**G. 精神病小计**

044. 老年,老年前期器质性精神病

045. 精神分裂症

046. 抑郁症

047. 其他精神障碍

**H. 神经系病计**

048. 脑膜炎

049. 癜痫

050. 急性感染性多发性神经炎

051. 帕金森病

052. 其他神经系疾患

**I. 眼及附器疾病计**

053. 青光眼

054. 白内障

055. 角膜疾病计

056. 其他眼及附器疾病

**J. 耳和乳突疾病计**

057. 中耳炎和乳突炎

058. 其他耳和乳突疾病

**K. 循环系统疾病小计**

059. 急性风湿热

060. 慢性风湿性心脏病

061. 心绞痛

062. 急性心肌梗死

063. 其他缺血性心脏病

064. 肺原性心脏病

065. 其他类型心脏病

066. 高血压病

067. 脑血管病

068. 下肢静脉曲张

069. 其他循环系统疾病

**L. 呼吸系统疾病小计**

070. 急性鼻咽炎（普通感冒）

071. 急性咽、喉、扁桃体和气管等

上呼道感染

072. 流行性感冒

073. 肺炎

074. 慢性咽、喉炎

075. 肺气肿

076. 其他慢性阻塞性肺病（COPD，含

慢支等）

077. 哮喘

078. 其他呼吸系统疾病（含急性下呼

吸道感染）

**M. 消化系统疾病小计**

079. 牙齿疾患

其他口腔或唾液腺及颌疾病

080. 急、慢性胃肠炎

081. 消化性溃疡

082. 阑尾疾病

083. 腹腔疝

084. 肠梗阻

085. 慢性肝病和肝硬变

086. 胆结石症和胆囊炎

087. 其他消化系统疾病

**N. 泌尿生殖系统疾病小计**

088. 肾炎和肾变病

089. 肾盂炎

090. 泌尿系统结石

091. 其他泌尿系统疾病

092. 前列腺增生或炎症

093. 其他男性生殖器官疾病

094. 乳房疾病

095. 输卵管炎和卵巢炎

096. 子宫阴道脱垂

097. 其他女性生殖器官疾病

**O.妊娠,分娩病及产褥期病发症小计**

098. 自然流产

099. *人工流产

100. 妊娠和分娩出血

101. 妊娠高血压综合症

102. *正常分娩

103. 梗阻性分娩

104. 产褥期病发症

105. 其他妊娠分娩病及产褥期并发症

**P. 皮肤和皮下组织疾病小计**

106. 痈和疖

107. 皮 炎

108. 其他皮肤和皮下组织疾病

**Q. 肌肉.骨骼系统和结缔组织疾病小计**

109. 类风湿性关节炎

110. 椎间盘疾病

111. 骨髓炎

112. 其他运动系病

**R. 先天异常小计**

113. 先天性心脏病

114. 其他先天异常

**S. 起源于围产期的情况小计**

115. 早产儿和未成熟儿

116. 产伤

117. 胎儿及新生儿窒息

118. 新生儿破伤风

119. 其他新生儿病

**T. 损伤和中毒小计**

120. 骨折

121. 脱位.扭伤和劳损

122. 颅内和体内损伤(包括神经)

123. 开放性创伤和血管损伤

124. 烧伤

125. 中毒和毒性效应

126. 其他损伤和中毒

**V. *其他小计**

127. 妊娠监护

128. 绝育

129. 为特殊治疗住院

130. 个人和人群的检查

131. 其他原因

999. 体征、病状和不明确情况

* 不为疾病。

注：⑴在填写疾病编码时，填写疾病名称相对应的三位阿拉伯数字（如012为乙型肝炎）； ⑵在疾病分类中，不可能把所有疾病名称均列上，如果没有某病名称，按此病是属于哪类疾病中的‘其他··系病’编码填写，如卵巢恶性肿瘤，填写恶性肿瘤类中的‘其他恶性肿瘤’，编码是030。对于疾病症状、体征不明确而不能作出疾病诊断的疾病填写999.
