# Supplementary material for: A study on the factors influencing the vulnerability of women of childbearing age to health poverty in rural western China
Source: Sci Rep. 2024 Jun 8;14:13219. doi: 10.1038/s41598-024-64070-z (PMC11162415; doi:10.1038/s41598-024-64070-z)
Supplement: Supplementary file 3 — Supplementary Information 3. [file 41598_2024_64070_MOESM3_ESM.pdf]

Replacement account: whether the questionnaire code:

1. Name of the household owner:______________Code: county _____ _____ township _____ village _____ household _____

telephone:

2. Number of registered population: _____ In the past 6 months, the number of permanent resident population (including permanent resident population): _____

Member code, member name, ID number

3. Home address:____________county____________Township (town)_____________village

4． Entry time: year, month, and day

5. Since the last investigation, whether the household is divided into households: (1) yes, (2) no

If divided, a total of divided into _____ households. This questionnaire investigates the No_______one-panelled door

Signature by the investigator:

Signature of the auditor:

Resident comrade:

shalom! We are the investigators of the "Innovative Payment System, Improve Health Benefits" project. The purpose of this survey is to understand the health and utilization of health services, and provide a basis for the formulation of medical and health policies in the region. I hope to get your cooperation. The content investigated will be used only for relevant analytical studies. We will strictly abide by the requirements of the Statistics Law of the People's Republic of China and keep the answers from you and your family confidential. I hope you can answer the following questions truthfully. Thank you very much for your cooperation!

Ningxia Medical University

The Health Commission of the Ningxia Hui Autonomous Region

**The code of the investigated members (01 is the head of the household, and the others are filled in in order according to the entry list)**		**01**	**02**	**03**	**04**	**05**	**06**	**07**
**A. Personal basic information (A1-A13 is answered by the head of the household or the insider, and the member code of the respondent is ___________)**								
A1	**Name of member: (Members include registered population and permanent resident population within 6 months)**							
A2	Relationship with the head of household: (1) head of household (2) spouse (3) children (4) andchildren (5) parents (6) grandparents (7) siblings (8) others							
A3	Gender: (1) male and (2) female							
A4	Ethnic: (1) Han (2) Mongolian (3) Hui (4) Tibetan (5) Uygur (6) Miao (7) Other							
A5	**Age: (one year old) (verified by the household registration book)**							
A6	Marital status: (1) unmarried (2) in marriage (3) divorce (4) widowed (5) other							
A7	Education level: (1) no school (2) primary school (3) junior high school (4) high school or above							
A8	occupation (main occupation): (1) farming (2) work (3) village cadres (4) village doctors (5) small business, small business (6) teachers (7) students (8) business owners (9) unemployed (10) others **(Please indicate)**							
A9	Whether to have worked in the field in the past year since the date of the investigation: (1) yes and (2) no							
A10	What kind of medical insurance policy are you currently participating in?(Can be selected more)							

The code of the investigated members (01 is the head of the household, and the others are filled in in order according to the entry list)		01	02	03	04	05	06	07
	(1) Urban and rural pooling medical insurance (3) Medical insurance for urban workers (4) commercial medical insurance (5) Other (6) did not participate							
**For the families who participate in the urban and rural pooling basic medical insurance (A10 choice 1), please answer the following questions of A10.3.1-A10.3.3.**								
A10.3.1	For the following outpatient clinics, which institution has the highest reimbursement rate:<br>(1) village clinics (2) township health center (3) county hospital (4) hospitals above the county level (5) the same high (6) do not know							
A10.3.2	Admission to the following institutions:<br>(1) township health centers (2) county hospitals (3) hospitals above the county level (4) the same high (5) do not know							
A10.3.3	If hospitalized in a hospital outside the county, is the reimbursement rate higher than those who are not?<br>(1) Yes. (2) No. (3) I do not know							
A11	**Who answers the following survey questions (Part B, C, and D must be fully investigated) (completed by the investigator):**<br>(1) Answer by yourself (jump question A12) (2) answer by others							
A11a	**Please fill in the member code of the representative answer person**							
A11.1	Others answer the reasons: (1) I go to other places to work (2) I am too small (3) my mind is not clear<br>(4) I do not want to answer (5) I am temporarily not at home (6) other							
A12	How do you feel about your health compared to your age?<br>(1) very good (2) good (3) general (4) poor (5) very bad (6) refuse to answer (7) do not know							
A13	Various body parameters							
A13.1	**stature (cm)**							

The code of the investigated members (01 is the head of the household, and the others are filled in in order according to the entry list)		01	02	03	04	05	06	07
A13.2	weight (kg)							
A13.3	**waistline (cm)**							
A13.4	**hip (cm)**							
**B. Injury and medical visits for the 14 days before the investigation**								
B1	Did you feel unwell within the 14 days before the survey?(1) Yes (2) No (jump C)							
B3.1	What illness or injury are you suffering from?**(Fill in the name of the disease) (with multiple diseases, fill in the name of multiple diseases)**							
B3.2	**(Fill in disease code) (according to National Health Service Survey)**							
B4	How many days did you stay in bed in the 14 days before the survey?(Days) (no bedridden, 0)							
B5	If you are a worker / farmer, how many days have you rested due to physical discomfort?(Days) (no work, 0)							
B6	If you are a student, how many days did you suspend from school because of physical discomfort?(Days) (no suspension, fill in 0)							
B7	Have you received any treatment (including self-treatment)?(1) Yes (jump B8) (2) No							
B7.1	If untreated, what is the main reason?**(After the answer, jump to ask C)**<br>(1) mild self-infection disease (2) economic difficulties (3) no time (4) inconvenient transportation inconvenience (5) no effective measures (6) others							
B8	How did you treat it?(1) See a doctor and have self-medical treatment within two weeks<br>(2) See a doctor within two weeks (ask B11)<br>(3) Pure and self-administered medical treatment							
B10	In the case of self-treatment, the source of your medication is: (up to three options)<br>**(1) existing at home (Q B11) (2) the drugstore (Q B10.11-B10.12)**							

The code of the investigated members (01 is the head of the household, and the others are filled in in order according to the entry list)		01	02	03	04	05	06	07
	**(4) given by others (jump question B11) (5) Others (jump question B11)**							
B10.11	Why did you choose to buy your medicine in a pharmacy instead of going to a medical institution?<br>(1) The types of drugs in pharmacies are more complete than those in medical institutions. (2) Self-infection disease is mild / there is no need to see a doctor<br>(3) pharmacies are cheaper than medicines in medical institutions (4) pharmacies have no time to go to medical institutions<br>(5) Inconvenient transportation to medical institutions (6) poor service in medical institutions (7) others							
B10.12	If you buy it at the pharmacy, how much yuan will you spend (including your children, family members, relatives to buy the medicine) in these 14 days?(Out-of-pocket expenses)							
**The following questions ask about visits within two weeks, and part B questions are visits within two weeks (B8= (3))**								
B11	How many times have you seen illness in the past 14 days?**(Time)**							
**The following questions are about the first visit (answered by me or the insider)**								
B12	Where was the first visit?(Please fill in the name of the clinic unit, including the name of the private clinic.)							
**opfaclvl-1**	Level 1, village clinic 2, township health center 3, county hospital 4, hospitals above the county level 5, private clinic 6 others							
B12.1	The main reasons for choosing the above-mentioned units are:<br>(1) close / convenient (2) reasonable fees (3) high technical level (4) good equipment conditions (5) rich drugs<br>(6) good service attitude (7) designated units (8) acquaintances (9) trusted doctors (10) referral (11) others							

The code of the investigated members (01 is the head of the household, and the others are filled in in order according to the entry list)		01	02	03	04	05	06	07
B13	Have you received the following treatment for this visit?							
B13.1	Intramuscular injection (1) Yes (2) No							
B13.2	Infusion therapy (1) Yes (2) No							
B13.3	Oral medication (1) Yes (2) No (Jump B13.5)							
B13.3.1	The medicine you take is: (1) Chinese medicine (2) western medicine (3) Chinese and western medicine have (4) do not know							
B13.3.2	Where did you come from?**(Up to three options)**<br>(1) bought by the medical institution (2) existing at home (3) bought by the pharmacy (4) given by others (5) others							
B13.5	Have you been referred to a superior medical institution this time?(1) Yes (2) No (Jump B13.6)							
B13.5.1	Which level of medical institution are you referred to?<br>(1) Township health centers and hospitals (2) county hospitals and (3) general hospitals above the county level<br>(4) Tuberculosis clinic of the center for Disease Control (epidemic prevention station) (5) others							
B13.6	Have you been referred from another medical institution?**(1) Yes (2) No (Jump Q B14)**							
B13.6.1	What level of medical facility are you visit before referral?<br>(1) Village clinics (2) township health centers (3) county hospitals (4) Centers for Disease Control and Prevention (5) medical institutions above the county level (6) others							
B14	How much is the total medical cost of this doctor?							
B14.1	How much yuan is the cash payment (including children, family members, relatives, etc.)?							
B14.1.1	Do you get "one-stop settlement" reimbursement from health insurance (each settlement has been deducted from the reimbursement)?							

The code of the investigated members (01 is the head of the household, and the others are filled in in order according to the entry list)		01	02	03	04	05	06	07
	(1) reimbursed (jump ask B16) (2) not reimbursed							
B14.1.2	Why didn't you get reimbursed?<br>(1) forget to bring social security card when seeking a doctor (2) did not participate in medical insurance<br>(3) I was told that the drugs and services I needed were not within the reimbursement scope. (4) the drug cost was very cheap and not worth reporting. (5) Other							
B16	You feel this about this visit:<br>(1) very satisfaction (2) satisfaction (3) general satisfaction (4) dissatisfaction (5) very dissatisfaction<br>**(Select (1) or (2), jump B16.2; select (3), select B17; select (4) or (5), answer B16.1)**							
B16.1	What are you most dissatisfied with by the medical institution?**(Up to three options)**<br>(2) Low technical level; (3) poor equipment conditions; (4) few types of drugs<br>(5) Poor service attitude (6) providing unnecessary services (including drugs and examinations) (7) unreasonable charges<br>(8) high medical expenses (9) complicated medical procedures (10) too long waiting time (11) others							
B16.2	What are you most satisfied with by the medical institution?**(Up to three options)**<br>(2) High technical level; (3) good equipment conditions; (4) many types of drugs<br>(5) Good service attitude (6) Failure to provide unnecessary services (including drugs and inspection) (7) reasonable charges<br>(8) low medical expenses (9) simple medical procedures (10) short waiting time (11) others							
**The following questions are about the second visit (answered by me or the insider)**								
B17	Where is the second visit?**(Please fill in the detailed name of the clinic unit, including the name of the private clinic.)**							

The code of the investigated members (01 is the head of the household, and the others are filled in in order according to the entry list)		01	02	03	04	05	06	07
opfaclvl-2	Level: 1, village clinic 2, township health centers 3, county hospitals 4, hospitals above the county level 5, private clinics 6, others							
B17.1	The main reasons for choosing the above-mentioned units are:<br>(1) close / convenient (2) reasonable fees (3) high technical level (4) good equipment conditions (5) rich drugs<br>(6) good service attitude (7) designated units (8) acquaintances (9) trusted doctors (10) referral (11) others							
B18	Have you received the following treatment after this visit?							
B18.1	Intramuscular injection (1) Yes (2) No							
B18.2	Infusion therapy (1) Yes (2) No							
B18.3	Oral medication (1) Yes (2) No (Jump B18.5)							
B18.3.1	The medicine you take is: (1) Chinese medicine (2) western medicine (3) Chinese and western medicine have (4) do not know							
B18.3.2	Where did you come from?**(Up to three options)**<br>(1) bought by the medical institution (2) existing at home (3) bought by the pharmacy (4) given by others (5) others							
B18.5	Have you been referred to a superior medical institution this time?(1) Yes (2) No (Jump B18.6)							
B18.5.1	Which level of medical institution are you referred to?<br>(1) Township health centers and hospitals (2) county hospitals and (3) general hospitals above the county level<br>(4) Tuberculosis clinic of the center for Disease Control (epidemic prevention station) (5) others							
B18.6	Have you been referred from another medical institution?**(1) Yes (2) No (jump B20)**							

The code of the investigated members (01 is the head of the household, and the others are filled in in order according to the entry list)		01	02	03	04	05	06	07
B18.6.1	What level of medical facility are you visit before referral?<br>(1) Village clinics (2) township health centers (3) county hospitals (4) CDC (5) medical institutions outside the county (6) others							
B20	How much is the total medical cost of this doctor?							
B20.1	How much yuan is the cash payment (including children, family members, relatives, etc.)?							
B20.1.1	Do you get "one-stop settlement" reimbursement from health insurance (each settlement has been deducted from the reimbursement)?<br>(1) reimbursed (jump question B 22) (2) not reimbursed							
B20.1.2	Why didn't you get reimbursed?<br>(1) forget to bring social security card when seeking a doctor (2) did not participate in medical insurance<br>(3) I was told that the drugs and services I needed were not within the reimbursement scope. (4) the drug cost was very cheap and not worth reporting. (5) Other							
B22	You feel about this visit: (1) very satisfied (2) satisfied (3) generally satisfied (4) not satisfied (5) very dissatisfied<br>**(Select (1) or (2), jump B22.2; select (3), select C1; select (4) or (5), answer B22.1)**							
B22.1	What are you most dissatisfied with by the medical institution?**(Up to three options)**<br>(2) Low technical level; (3) poor equipment conditions; (4) few types of drugs<br>(5) Poor service attitude (6) providing unnecessary services (including drugs and examinations)<br>(7) unreasonable charges<br>(8) high medical expenses (9) complicated medical procedures (10) too long waiting time (11) others							
B22.2	What are you most satisfied with by the medical institution?**(Up to three options)**<br>(2) High technical level; (3) good equipment conditions; (4) many types of drugs							

The code of the investigated members (01 is the head of the household, and the others are filled in in order according to the entry list)		01	02	03	04	05	06	07
	(5) Good service attitude (6) Failure to provide unnecessary services (including drugs and inspection) (7) reasonable charges<br>(8) low medical expenses (9) simple medical procedures (10) short waiting time (11) others							
**C. Hospitalization status within one year**								
C1	In the past year, have you been diagnosed by a doctor that you need hospitalization?(1) Yes (2) No (jump D)							
C1.1	How many times in the past year (did the doctor diagnose that you need to be hospitalized)?							
C2	How many years did your doctor diagnose a hospitalization for you in the past year and you did not?<br>**(Fill in the specific times, if not, fill in 0 and ask C3 instead)**							
C2.1	Main reasons for no hospitalization:<br>(1) No need (2) no time (3) economic difficulties (4) poor service (5) too high price (6) no beds (7) other							
C3	How many times have you been in the hospital in the past year?(Fill in the specific times, if not, fill in 0 and ask D)							
C3.1	How many days have you lived in in the past year?**(Fill in the specific number of days.)**							
**Most recent hospitalization in the past year**								
C4	The name of your illness hospitalized due to illness or injury poisoning?<br>**(Fill in the name of the disease during the survey, and turn in the disease code when checking the questionnaire)**							
C4.1.2	**Disease 1**							
C4.1.3	**Disease 2**							
C4.1.4	**Disease 3**							

**The code of the investigated members (01 is the head of the household, and the others are filled in in order according to the entry list)**		**01**	**02**	**03**	**04**	**05**	**06**	**07**
C4.1	Time of admission for this hospitalization: (years)							
C4.1.1	**(moon)**							
C4.2	Is the name of the medical institution hospitalized this time?							
INPFACLV	Level: 1, township health centers 2, county hospitals 3, hospitals above the county level 4, others							
C4.2.1	Where is the medical institution that be hospitalized this time?(1) County (answer C4.3-C4.3.1) **(2) Outside the county (answer C4.2.2-C4.2.4)**							
If C4.2.1 Select (2), please answer C4.2.2-C4.2.4								
C4.2.2	What is the reason why you chose a medical institution outside the county for this hospitalization?<br>(1) Good environmental conditions (2) high technical level (3) serious condition (4) good equipment conditions<br>(5) many types of drugs (6) recommended by doctors (7) close to where I live (8) others							
C4.2.3	You go to the hospital outside the county this time, whether through the referral of the county hospital?(1) Yes (2) No (Jump C 4.4)							
C4.2.4	If you transfer, where did you transfer from?(1) Township hospitals (2) county hospitals (3) county Hospital of Traditional Chinese Medicine (4) others							
If C4.2.1 selects (1), please answer C4.3-C4.3.1								
C4.3	Is this hospitalization a transfer?(1) Yes (2) No (Jump C4.4)							
C4.3.1	If you transfer, where did you transfer from?<br>(1) Township hospitals (2) county (district) hospitals (3) city (local) hospitals (4) provincial hospitals<br>(5) Military hospitals (6) county traditional Chinese medicine hospitals (7) traditional Chinese							

**The code of the investigated members (01 is the head of the household, and the others are filled in in order according to the entry list)**		**01**	**02**	**03**	**04**	**05**	**06**	**07**
	medicine hospitals above the city (8) private hospitals (9) others							
C4.4	Number of days for this hospitalization: (days)							
C4.5	Have you ever had an operation in this hospital? No. (1) Yes. (2) No							
C4.6	If you are working / farming, the days of work due to this hospitalization?**(Including hospitalization days, no 0)**							
C4.6.1	If you are a student, are the number of days suspended from this hospitalization?**(Including hospitalization days, no 0)**							
C4.6.2	How many bed days at home before and after this hospitalization?**(Excluding hospitalization days, no 0)**							
C4.7	Your discharge is due to: (1) the doctor asked (2) the doctor asked (3) (4) other reasons<br>**(Select (3) answer C4.7.1, other options jump question C4.8)**							
C4.7.1	If you request discharge, the reasons:<br>(1) long illness (2) economic difficulties (3) limited hospital conditions (4) poor service attitude (5) others							
C4.8	In the past year, have you participated in the basic medical insurance for urban and rural areas?<br>(1) Yes (2) No (Jump C4.10)							
C4.8.1	If you participate in the urban and rural pooling basic medical insurance, the way you pay the medical expenses in this hospitalization is:<br>(1) They pay all in advance, and then go to the medical insurance center for reimbursement<br>(2) The hospital directly reduces the reimbursed expenses (jump question C4.10)							
If C4.8.1 selects (1), answer C4.9 and C4.9.1; if C4.8.1 selects (2), answer C4.10								
C4.9	How much yuan did you pay (including children, family, relatives) pay?**(Excluding bus fare, escort, red envelope)**							

The code of the investigated members (01 is the head of the household, and the others are filled in in order according to the entry list)		01	02	03	04	05	06	07
C4.9.1	How much yuan did the medical insurance center reimburyou for this hospitalization?**(Jump Q C4.11)**							
C4.10	How much yuan did you pay to yourself (including your children, family members and relatives) for the hospitalization expenses?**(Excluding reimbursement reduction, fare, escort, red envelope)**							
C4.11	How much is the cost of the hospital for travel, nutrition, food and escort expenses (yuan)?(No fill 0)							
C4.13	You feel about this hospitalization:<br>(1) very satisfaction (2) satisfaction (3) general satisfaction (4) dissatisfaction (5) very dissatisfaction<br>**(Select (1) or (2), jump question C4.13.2; select (3), jump C5; select (4) or (5), answer C4.13.1)**							
C4.13.1	What are you most dissatisfied with in your inpatient unit?**(Up to three options)**<br>(2) Low technical level; (3) poor equipment conditions; (4) few types of drugs<br>(5) Poor service attitude (6) providing unnecessary services (including drugs and examinations) (7) unreasonable charges<br>(8) High medical expenses (9) complicated medical procedures (10) too long waiting time (11) poor medical environment<br>(12) the treatment is not thorough or the treatment effect is not obvious (13) others							
C4.13.2	What are you most satisfied with in your inpatient unit?**(Up to three options)**<br>(2) High technical level; (3) good equipment conditions; (4) many types of drugs<br>(5) Good service attitude (6) Failure to provide unnecessary services (including drugs and inspection) (7) reasonable charges<br>(8) Low medical expenses (9) simple procedures for seeing a doctor (10) short waiting time							

The code of the investigated members (01 is the head of the household, and the others are filled in in order according to the entry list)		01	02	03	04	05	06	07
	(11) good medical environment<br>(12) complete treatment or obvious treatment effect (13) others							
**One hospitalization before the latest hospitalization in the past year (if two or more admissions in a year, otherwise D)**								
C5	The name of your illness hospitalized due to illness or injury poisoning?<br>**(Fill in the name of the disease during the survey, and turn in the disease code when checking the questionnaire)**							
C5.1.2	**Disease 1**							
C5.1.3	**Disease 2**							
C5.1.4	**Disease 3**							
C5.1	Time of admission for this hospitalization: (years)							
C5.1.1	**(moon)**							
C5.2	Is the name of the medical unit in this hospitalization?**(Write a detailed name.)**							
INPFACLV	Level: 1, township health centers 2, county-level hospitals 3, hospitals above the county level 4, others							
C5.4	Number of days for this hospitalization: (days)							
If C4.8.1 selects (1), answer C5.9 and C5.9.1; if C4.8.1 selects (2), answer C5.10								
C5.9	How much yuan did you pay (including children, family, relatives) pay?**(Excluding bus fare, escort, red envelope)**							
C5.9.1	How much yuan did the medical insurance center reimburyou for this hospitalization?**(Jump Q C5.11)**							
C5.10	How much yuan did you pay to yourself (including your children, family members and relatives) for the hospitalization expenses?**(Excluding reimbursement reduction, fare, escort, red envelope)**							

The code of the investigated members (01 is the head of the household, and the others are filled in in order according to the entry list)		01	02	03	04	05	06	07
C5.11	How much is the cost of the hospital for travel, nutrition, food and escort expenses (yuan)?(No fill 0)							
**D. Chronic diseases (please refer to the training manual for a list of chronic diseases)**								
D1	Did you have a chronic disease diagnosed by a doctor in the past?(1) Yes (2) No (jump E)							
D1.1.4	**Which of the following chronic diseases do you have (multiple options)**<br>(1) hypertension (2) diabetes mellitus (3) intervertebral disc disease<br>(4) Cerebrovascular disease (5) chronic gastroenteritis (6) coronary heart disease<br>(7) Rheumatoid arthritis (8) chronic obstructive pulmonary disease (9) Other_________(Fill in the disease code)							
D 1.1.5	How many chronic diseases are there there?**(Number of fills)**							
D 1.1.6	The length of disease of the earliest diagnosed chronic disease?							
D2	How many times have you seen these diseases in the last three months?**(If not, fill in 0 and ask D3.5)**							
D3.1	Your primary visits for these conditions in the last three months are:<br>(1) Village clinics (2) township health centers (3) county hospitals (4) private clinics (5) others							
D3.2	Have you already applied for the outpatient serious illness medical certificate?**(1) Yes (2) No (Jump D3.4)**							
D3.2.1	If you participate in the urban and rural planning, the way you pay the medical expenses when seeing these diseases is:<br>(1) They pay all in advance, and then go to the medical insurance center for reimbursement<br>(2) The hospital directly reduces the reimbursed expenses (choose ⑵, ask D3.4)							
D3.3	In the last three months, how much of the medical expenses caused by these diseases for you (or children, family members, relatives)?**(Excluding bus fare, escort, red envelope)**							

**The code of the investigated members (01 is the head of the household, and the others are filled in in order according to the entry list)**		**01**	**02**	**03**	**04**	**05**	**06**	**07**
D3.3.2	How many yuan did the medical insurance center pay you for the medical expenses incurred by these diseases in the last three months?**(Jump Q D3.5)**							
D3.4	How many yuan did you pay to yourself (or your children, family members, relatives) for the medical expenses caused by these diseases in the last three months?**(Excluding reimbursement reduction, fare, escort, red envelope)**							
D3.5	In the last three months, how much yuan did you spend to buy medicine in the drugstore (including children, family and relatives for you) because of these diseases?**(No fill 0)**							
**E. Health and behavior of members 15 years and older (population born in 2007 and before) (ask F without this age)**								
E1	Today you take action:<br>(1) can walk around, without any difficulties (2) some action inconvenience (3) sick in bed							
E2	Today in your self-care (washing and dressing):<br>(1) No problems (2) some problems (3) unable to wash or wear clothes							
E3	Today, you are engaged in ordinary activities (work, reading or doing housework):<br>(1) No problems in daily activities (2) some problems (3) inability to engage in daily activities							
E4	Your body pain or discomfort today:<br>(1) No pain or discomfort (2) self-conscious moderate pain or discomfort (3) self-conscious extreme pain or discomfort							
E5	Today in your anxiety or depression:<br>(1) Do not feel anxious or depressed; (2) consciously moderate anxiety or depression; (3) consciously extreme anxiety or depression							
E6	Please point out on the scale that is the best indicator of your health today<br>├──┼──┼──┼──┼──┼──┼──┼──┼──┼──┤<br>0 10 20 30 40 50 60 70 80 90 100							

The code of the investigated members (01 is the head of the household, and the others are filled in in order according to the entry list)		01	02	03	04	05	06	07
	Worst health, best health condition							
E7	In general, your health status is: (1) very good (2) very good (3) good (4) general (5) poor							
E8	Compared to a year ago, you think your health status is:<br>(1) better than a year ago (2) better than a year ago (3) similar to a year ago<br>(4) some worse than one year ago (5) much worse than one year ago							
I1	smoke							
I1.1	Do you smoke?(1) Never suction (jump ask I4) (2) occasionally suction (3) often suction (4) has quit smoking (jump ask I1.5)							
I1.2	How old did you start smoking?**(one full year of life)**							
I1.3	How many cigarettes do you take per day: (count)							
I1.4	How much do you cost to smoke every month?(Q I4)							
I1.5	How long have you taken to quit smoking (years)							
I1.5.1	The main reasons for quitting smoking (with multiple choices)?<br>(1) disease (2) disease prevention (3) economic reasons (4) family opposition (5) environmental restrictions<br>(6) Set up the image (7) through the publicity and education (8) through the doctor's advice (9) other (10) do not know							
I 4	drink							
I 4.1	Do you drink alcohol?(1) Never drink (jump question I 5) (2) occasionally drink (3) often drink (4) have quit drinking (jump ask I4.5)							
I4.2	How old did you start drinking?**(one full year of life)**							
I4.3	How much you drink every day: (fill in specific weight)							
I4.4	How much do you cost to drink every month?(Q I5)							

The code of the investigated members (01 is the head of the household, and the others are filled in in order according to the entry list)		01	02	03	04	05	06	07
I4.5	How many years have you been sober?							
I4.5.1	The main reason for drinking (this question is optional)?<br>(1) disease (2) disease prevention (3) economic reasons (4) family opposition (5) environmental restrictions<br>(6) Set up the image (7) through the publicity and education (8) through the doctor's advice (9) other (10) do not know							
I5	Exercise exercise							
I5.1	How many times do you have an average of conscious physical exercise every week (including square dancing, walking exercise, walking, running, morning exercise, recess exercises, physical education classes, extracurricular sports classes, workshop exercises, etc.)?<br>(1) 6 times or more (2) 3-5 times (3) 1-2 times (4) less than 1 time (5) Never exercise (jump ask M)							
I5.2	What is your average physical exercise time?<br>(1) more than 1 hour (2) 30 minutes-1 hour (3) less than 30 minutes							
**M. Survey of mental health status of members aged 15 years and above: Please fill in the answer number in the space according to your situation in the past two weeks.(To answer in person, if I do not ask F)**								
M22	No energy or no interest in doing things<br>① No ② has a few days ③ more than a week ④ almost every day							
M23	Feel depressed, depressed or desperate<br>① No ② has a few days ③ more than a week ④ almost every day							
M24	Difficulty falling asleep, sleeping poorly or sleeping too much<br>① No ② has a few days ③ more than a week ④ almost every day							
M25	Feeling tired or lifeless							

The code of the investigated members (01 is the head of the household, and the others are filled in in order according to the entry list)		01	02	03	04	05	06	07
	① No ② has a few days ③ more than a week ④ almost every day							
M26	Loss of appetite or eat too much<br>① No ② has a few days ③ more than a week ④ almost every day							
M27	Feel too bad or fail, or let yourself and your family down<br>① No ② has a few days ③ more than a week ④ almost every day							
M28	It is difficult to concentrate on doing things, such as reading newspapers and watching TV<br>① No ② has a few days ③ more than a week ④ almost every day							
M29	Movement or speech speed becomes very slow, or just the opposite, appear irritability or fidgeting more than usual<br>① No ② has a few days ③ more than a week ④ almost every day							
M30	Have the idea of committing suicide or hurting yourself in some way<br>① No ② has a few days ③ more than a week ④ almost every day							
**F. Questionnaire of married women aged 15-49 (required) (1972-2007) (if not in this age group, ask G)**								
F1.1	In the past year, have you ever done free gynecology (breast, uterus) examination? No. (1) Yes. (2) No							
F1.2	Have you ever had a free cervical test in the past year?**(Including cervical smear, TCT, HPV examination, etc.)**<br>No. (1) Yes. (2) No							
F1.3	Have you ever had a free breast test in the past year?**(Including B-ultrasound, molybdenum target, etc.), (1) Yes, (2) No**							
F2	Did you delivered after February 1,2019 (1) Yes (2) No (jump to G1)							
F3	Last delivery, how many prenatal tests (times)?**(Never done, fill in 0 and ask F4)**							
F3.1	The first prenatal examination was done during the first week of pregnancy?**(circumference)**							
F3.2	Where did you have a prenatal check-up?**(Up to three options)**							

**The code of the investigated members (01 is the head of the household, and the others are filled in in order according to the entry list)**		**01**	**02**	**03**	**04**	**05**	**06**	**07**
	(1) county / district and above hospitals (2) county / district and above traditional Chinese medicine hospitals (3) maternal and child health care institutions<br>(4) Township street health centers (5) community health service center (6) family planning guidance station<br>(7) Health clinic / office / station (8) others							
F4	**Place of delivery: (select (8) F4.3)**<br>(1) Hospitals above the county level (2) county hospitals (3) maternal and child health care institutions (4) township and sub-district health centers<br>(5) Community health service center (6) Family planning guidance station (7) clinic / office / station (8) home (9) other							
F4.3	This delivery you are (1) natural birth (2) cesarean section (3) other							
F6	How many times has a doctor or health worker visited your home within 42 days after delivery?(Times) (not filled in 0)							
**G. Children under 7 questionnaire (preferably answered by the mother or not by the person who best knows the child) (born after 2015) (jump H)**								
G1	What does the child's mother code for in the questionnaire?**(If the mother is not investigated, code the respondent)**							
G1.1	Is the child being breastfed? No. (1) Yes. (2) No							
G2	In the past 12 months, the child has received several physical examination (times)?**(Not including tests done for the disease)**							
G3	Does the child have a planned immunization card or manual?(1) have (2) have no (3) do not know							
G5	Where is the usual immunization process?<br>(1) CDC (2) Health Center (3) Community Health Service Center (4) Village clinic / office /							

The code of the investigated members (01 is the head of the household, and the others are filled in in order according to the entry list)		01	02	03	04	05	06	07
	station (5) Others							
G8	Have you received other self-funded vaccines?<br>(1) Yes (2) No (jump question G9) (3) do not know							
G8.1	What kind of self-funded vaccination?							
G9	Has the child ever been diagnosed with anemia? No. (1) Yes. (2) No							
G10	Are the children left-behind children? No. (1) Yes. (2) No							
**H.45 and older (born in 1977 or before) (ask J)**								
H11	Do you need any help when you take a shower?<br>(1) oneself can completely (2) some difficulties (3) need help (4) can not do it at all							
H12	Do you need any help when you dress up?<br>(1) oneself can completely (2) some difficulties (3) need help (4) can not do it at all							
H13	Do you need any help when you go to the bathroom?<br>(1) oneself can completely (2) some difficulties (3) need help (4) can not do it at all							
H14	Do you need help during your indoor activities?<br>(1) oneself can completely (2) some difficulties (3) need help (4) can not do it at all							
H15	Can you control the defecation?<br>(1) oneself can completely (2) some difficulties (3) need help (4) can not do it at all							
H16	Do you need help from others at dinner?<br>(1) oneself can completely (2) some difficulties (3) need help (4) can not do it at all							
H17	Do you need help to wash your face, comb your hair, brush your teeth and shave?<br>(1) oneself can completely (2) some difficulties (3) need help (4) can not do it at all							
H18	Do you need help from bed to a chair?<br>(1) oneself can completely (2) some difficulties (3) need help (4) can not do it at all							

The code of the investigated members (01 is the head of the household, and the others are filled in in order according to the entry list)		01	02	03	04	05	06	07
H19	Do you need help up the stairs (up and down the stairs, with a cane is also independent, can be used on the steps instead)<br>(1) oneself can completely (2) some difficulties (3) need help (4) can not do it at all							
**L. Survey on children aged 45 and above (born in 1977 or before) (answered by myself) (ask J)**								
L1	Where was your birthplace?(1) This Province; (2) other provinces							
L2	Have you ever been hungry during childhood and adolescence?(1) is (2) is not (3), I do not know							
L3	Can children and adolescents be treated in time (go to the hospital or buy medicine)?(1) Can (2) can not (3), do not know							
L4	How are you doing during childhood and adolescence?(1), very good (2) good (3) general (4) bad (5), very bad							
L5	Are all the parents alive in children and adolescents?<br>(1) Father or mother in (2), parents in (3) parents are absent (jump ask L7.1)							
L6.1	How is your father doing during childhood and adolescence?(1), very good (2) good (3) general (4) poor (5) very bad							
L6.2	How is your mother doing during childhood and adolescence?(1), very good (2) good (3) general (4) poor (5) very bad							
L7.1	Did your father ever go to school?(1) on (2), not on							
L7.2	Did your mother ever go to school?(1) on (2), not on							
**J. Family income and expenditure**								
**Basic family situation**								
**The code of the investigated member answering this section is (answered by the householder or the insider):**								
**Whether the J1 family owns the following property: (1) Yes (please fill in the quantity) (2) No (Fill in 0)**								

**The code of the investigated members (01 is the head of the household, and the others are filled in in order according to the entry list)**	**01**	**02**	**03**	**04**	**05**	**06**	**07**
J1.2 bicycle (electric vehicle)							
J1.5 color TV							
J1.7 motorcycle							
The J1.8 automobile No							
J1.9 refrigerator							
J1.10 Washing machine							
J1.11 phone (including mobile phone, landline)							
J1.12 Agricultural machinery							
J1.13 Satellite receiver, VCD, DVD, audio, etc. (only one item)							
J1.14 camera, video camera, etc. ____________							
J1.15 air conditioning							
J1.16 cattle							
J1.17 sheep							
J1.18 horse, donkey, mules							
**J1.19 pig (Note: when registering basic information, if it is a Hui family, do not ask this question)**							
J1.20 Dated land							
J1.21 Mountain area							
J1.22 computer (including desktop and laptop computers)							
J1.23 Kitchen appliances (microwave oven, induction cooker, electric wok, range hood, etc.) (how many pieces to fill in)							
J1.24 Solar energy							
J1.25 Chicken, duck and other poultry (how many birds are filled in)							
**Please answer the following questions (J2-J8) according to the actual situation of the house**							
J2 your housing type: (1) brick and concrete (2) brick and wood (3) civil (4) whole brick (hard top) (5) cave (6) others							
J2.1 What material is the land of your house?(1) land (2) brick land (3) ceramic tile (4) wood floor (5) floor leather (6) others							

The code of the investigated members (01 is the head of the household, and the others are filled in in order according to the entry list)	01	02	03	04	05	06	07
J2.2 What year did your house be built?year							
J2.3 How big is your house?square meter							
J3 The main types of drinking water in your home: (1) tap water (2) mountain spring water (3) hand press well water (4) cellar water (5) well water (6) river and lake water (7) pond ditch water (8) other							
J4 type of your toilet: (1) flushing (2) biogas or three tank type (3) double urn funnel type (4) pit or water flushing (5) toilet (6) dry toilet (7) no toilet (8) other							
J5 what fuel does your home usually use for cooking (at most choose two kinds): (1) coal (2) electricity (3) kerosene (4) natural liquefied gas / natural gas (5) wood, wood, etc. (6) charcoal (7) other							
Is the J6 housing separated from the kitchen? No. (1) Yes. (2) No							
J8 Distance from your home to the nearest medical point (1 km =1000 m; 1 li =500 m)							
J8.1 The nearest village clinic to your home							
J8.1.1 km number:							
J8.1.2 The most commonly used modes of transportation: (1) walking (2) buses (3) bicycles (4) motorcycles (5) others (self-drive, car / private car)							
J8.1.3 Time required: minutes							
J8.2 Your home is the nearest township health center							
J8.2.1 km number:							
J8.2.2 The most commonly used modes of transportation: (1) walking (2) buses (3) bicycles (4) motorcycles (5) others (self-drive, car / private car)							
J8.2.3 Time required: minutes							
J8.3 The nearest county hospital to your home							
J8.3.1 km number:							
J8.3.2 The most commonly used modes of transportation: (1) walking (2) buses (3) bicycles (4) motorcycles (5) others (self-drive, car / private car)							
J8.3.3 Time required: minutes							
J8.4 The nearest medical point to your home is (the nearest point): (1) village clinic (2) township health center (3) county hospital							
J9 Is your family listed as a registered poor household or a low-income household?(1) No (jump J10) (2) Yes.**2**							

**The code of the investigated members (01 is the head of the household, and the others are filled in in order according to the entry list)**	**01**	**02**	**03**	**04**	**05**	**06**	**07**
J9.1 When participating in the urban and rural pooling, does your family get the reduction of the insurance fees?(1) has got (2) did not get (3) is not clear							
**Family lending**							
J10.2 In the past 6 months, has your family ever borrowed money from others for medical treatment? No. (1) Yes. (2) No							
J10 Is your family in debt right now?(1) (2) no (jump J11) (3) not clear (jump J11)							
J10.1 If there are any arrears, the main reasons for the arrears are:<br>(1) buy food (2) building a house repair (3) medical treatment (4) children go to school (5) pay taxes (6) red and white wedding (7) agricultural production (8) other (indicated)							
J10.1.1 How much is the debt owed?							
J10.1.2 How much is the repayment interest due to debt?							
J11 Is your family borrowing money to someone else now?(1) Yes (2) no (jump question J12) (3) not clear (jump question J12)							
J11.1?							
**productive outlays**							
**J12 How much yuan did your family spend last year on production (such as cattle, fertilizer, seeds, agricultural machinery, etc.)?**							
**Consumer expenditure (unit: yuan)**							
J13 usually a month, the cash expenditure of the following items							
J13.1 Food expenditure (food, meat, vegetables, fruit, oil, oil, salt, sauce, vinegar, tea, tobacco, wine, etc.)							
J13.3 Daily necessities, such as soap, paper, pen, etc							
J13.4 Electricity fee, water fee, heating fee, cooking fuel fee, telephone fee, express fee, Internet fee, etc							
J13.5, rent, land and rent, etc							
J13.7 Transportation cost (bus fare, fuel cost)							
J13.8 Culture, recreational activities, etc							
**J14 Cash expenditure (in RMB) for the past year (12 months)**							
J14.1 for the clothes							

**The code of the investigated members (01 is the head of the household, and the others are filled in in order according to the entry list)**	**01**	**02**	**03**	**04**	**05**	**06**	**07**
J14.2 Tuition fees, books, school supplies, etc. (including the living expenses of students studying outside at home)							
J14.3 Premium of overall urban and rural integrated basic medical care							
**J14.4 Drugs, medical expenses, etc. (only including the amount of medical expenses paid by oneself and his family members)**							
J14.5 Gift expenses, such as dowry, New Year gifts, wedding gifts, funeral expenses, gifts to relatives and friends							
**J14.8 All kinds of insurance expenses (excluding the insurance premium of urban and rural integrated basic medical care)**							
J14.9 Expendable on purchasing durable goods, such as mobile phones, computers, TV, furniture, electric fans, bicycles, motorcycles, cars, refrigerators, air conditioners, etc							
J14.10 Building and repairing and buying houses							
**J14.11 Other (not included in the monthly and annual expenditures)**							
**Savings status (unit: RMB)**							
J15 last year your family money enough to spend enough, how much money can be left?**(If not, fill 0)**							
**Self-produced items**							
J16 is the value of the self-produced goods consumed by the whole family last year<br>(If the respondent can answer the consumption quantity, the amount can be obtained according to the unit price multiplied by the quantity, but if the respondent cannot answer the quantity, please inform the approximate amount and fill in the amount column; the total output and sales quantity are used to calculate the consumption quantity, and there is no need to fill in the consumption quantity.)							

**Self-produced items**	**Total output (jin)**	**Sales quantity (jin)**	**Self-consumption quantity (jin)**
J16.1 for wheat			
J16.2 in maize			
J16.3 for vegetables			
J16.4 Meat meat			
**Including: pork (note: if it is a Hui family, do not ask this question)**			

The code of the investigated members (01 is the head of the household, and the others are filled in in order according to the entry list)		**01**	**02**	**03**	**04**	**05**	**06**	**07**
mutton								
beef								
Chicken, duck								
J16.5 for Eggs								
J16.6 for fruit								
J16.7 Buckwheat								
J16.8 flax								
J16.9 for potatoes								
J16.10 Other								
J16.11barbary wolfberry								
**Family income**								
**J17 Family income (yuan) in the past year (including the money sent back by family members of migrant workers)**								
J17.1 Money (yuan) sent back by family members who have worked outside in the past year								
**K . Other circumstances (K 1-K8 is answered by the head of the household or the insider, and the member code of the respondent is ___________)**								
K 3	Have you lost your labor force?(1) Yes. (2) No. (3) I do not know							
K 3.1	Are you a disabled person? And (1) Yes and (2) No							
K 4	Do you have a health file?(1) Yes. (2) No. (3) I do not know							
K5	Do you enjoy a family doctor contract service?(1) Yes. (2) No. (3) I do not know							
K6	Have you ever had a physical examination in the past one year?(1) Yes. (2) No. (3) I do not know							
K7	Do you know about the "inpatient treatment before payment" system?(1) Yes. (2) No. (3) I do not know							
K7.1	In the past year, if you have been in hospital, have you enjoyed the "hospitalization after							

The code of the investigated members (01 is the head of the household, and the others are filled in in order according to the entry list)		01	02	03	04	05	06	07
	payment" system? **(C3 item 1 to answer this question) (1) Yes (2) No (3) Do not know**							
K8	If you have ever been in a hospital in the past year, have you ever had an operation?**(C3 for item 1)** (1) Yes. (2) No. (3) I do not know							
K 9	The number of missed work days (including outpatient service, hospitalization, and chronic diseases) in the past one year?**(Fill in the specific number of days.)**							
K10	Whether the family is an empty-nest elderly family is (1) yes and (2) no							
**Follow-up**								
Family follow-up at J18								
J18.1 Personal follow-up status								

Description of the family follow-up status:

1= participation in 2009,2011,2012,2015,2019,2022 surveys 2= participation in 2011,2012,2015,2019,2022 surveys 3= 2009,2012,2015,2019,2022 surveys

4= surveys in 2009,2011,2015,2019,20225= in 2009,2011,2012,2019,20226= in 2009,2011,2012,2015,2022

7= participation in 2012,2015,2019,20228= participation in 2011,2015,2019,20229= 2011,2012,2019,2022

10= participation in 2011,2012,2015,2022 surveys 11= participation in 2009,2015,2019,2022 surveys 12= 2009,2012,2019,2022 surveys

13= participation in 2009,2012,2015,2022 surveys 14= participation in 2009,2011,2019,2022 surveys 15= 2009,2011,2015,2022 surveys

16= participation in 2009,2011,2012,2022 surveys 17= participation in 2015,2019,2022 survey 18= participation in 2012,2019,2022 surveys

19= participation in the 2012,2015,2022 survey 20= participation in the 2011,2019,2022 survey 21= participation in the 2011,2015,2022 surveys

22= participation in 2009,2019,2022 surveys 23= participation in 2009,2015,2022 surveys 24= participation in 2009,2011,2022 surveys

25= participation in 2009,2012,2022 survey 26= participation in 2011,2012,2022 survey 27= participation in 2009,2022 survey

28= Participation in the 2011,2022 survey 29= Participation in the 2012,2022 survey 30= Participation in the 2015,2022 survey

31= participation in 2019,2022 survey 32= only participation in 2022 survey 33=2022 No follow-up: happened to no one at home during the survey

34=2022 No follow-up to: the whole family goes out for a long time 35=2022 No follow-up to: the whole family has moved out

The code of the investigated members (01 is the head of the household, and the others are filled in in order according to the entry list)	01	02	03	04	05	06	07
**Personal follow-up status:**<br>1 = follow-up to 2 = new family members 3 = no follow-up to: marriage / household 4 = no follow-up to: death 5 = no follow-up to: long-term outing 6 = no follow-up to: other reasons							

----------Finish the----------
