# Supplementary material for: A study on the factors influencing the vulnerability of women of childbearing age to health poverty in rural western China
Source: Sci Rep. 2024 Jun 8;14:13219. doi: 10.1038/s41598-024-64070-z (PMC11162415; doi:10.1038/s41598-024-64070-z)
Supplement: Supplementary file 4 — Supplementary Information 4. [file 41598_2024_64070_MOESM4_ESM.pdf]

替代户：　是　否

问卷编码：

编码：县_____　乡镇_____　村_____　户_____

电话：

成员编码　成员姓名　身份证号

1. 户主姓名：_______________

2. 户籍人口数：_____　过去 6 个月，常住人口数（含常住户籍人口）：_____

3. 家庭住址：____________县____________乡（镇）_____________村

4. 入户时间：　　年　　月　　日

5. **自上次调查起，该户是否分家：　(1)是　(2)否**

如果分家，总共分为_____户。本问卷调查的是第_______户

填表时间　　年　　月　　日　　调查员签名:

核查日期　　年　　月　　日　　审核人签名:

住户同志：

您好！我们是“创新支付制度，提高卫生效益”项目的调查员。此次调查旨在了解居民健康和卫生服务利用情况，为制定本地区医疗卫生相关政策提供依据，希望得到您的配合。所调查的内容我们仅用于相关的分析研究。我们会严格遵守中华人民共和国《统计法》的要求，对您和您家人回答的内容保密。下面的问题希望您能够如实回答。非常感谢您的合作！

宁夏医科大学

宁夏回族自治区卫生健康委员会

被调查成员编码 （01为户主，其他按入户名单给出顺序依次填写）		01	02	03	04	05	06	07
A. 个人基本情况（A1-A13由户主或知情人统一回答，回答人的成员编码是__________）								
A1	成员姓名： **（成员包括户籍人口和6个月内常住人口）**							
A2	与户主关系： (1)户主 (2)配偶 (3)子女 (4)孙子女<br>(5)父母 (6)祖父母 (7)兄弟姐妹 (8)其他							
A3	性别： (1)男 (2)女							
A4	民族： (1)汉族 (2)蒙族 (3)回族 (4)藏族 (5)维族 (6)苗族 (7)其它							
A5	年龄：**（周岁）（用户口本核实）**							
A6	婚姻状况： (1)未婚 (2)在婚 (3)离婚 (4)丧偶 (5)其他							
A7	文化程度： (1)没上过学 (2)小学 (3)初中 (4)高中及以上							
A8	职业（**主要职业**）： (1)务农 (2)务工 (3)村干部 (4)村医生 (5)小生意、小买卖<br>(6)教师 (7)学生 (8)企业主 (9)无业 (10)其它____________**(请注明)**							
A9	自调查之日起过去一年来是否到外地打过工： (1)是 (2)否							
A10	您目前参加了哪种医疗保险？（可多选）<br>(1)城乡统筹医疗保险 (3)城镇职工医疗保险 (4) 商业医疗保险 (5) 其他 (6)没参加							
对于参加城乡统筹基本医疗保险的家庭（A10选择1），请回答以下A10.3.1-A10.3.3的问题。								
A10.3.1	如果到下列机构看门诊，哪类机构报销比例最高：<br>(1)村卫生室 (2)乡镇卫生院 (3)县医院 (4)县级以上医院 (5)一样高 (6)不知道							
A10.3.2	如果到下列机构住院，哪类机构报销比例最高：<br>(1)乡镇卫生院 (2)县医院 (3)县级以上医院 (4)一样高 (5)不知道							
A10.3.3	如果在县外医院住院的话，被转诊的病人是否比没有被转诊的病人的报销比例高？<br>(1)是 (2)否 (3)不知道							
A11	下列调查问题（**B,C,D部分必须全部调查**）**由谁回答**（调查员填写）：<br>(1)自己回答 **(跳问A12)** (2)由他人代答							
A11a	**请填写代答人的成员编码**							

被调查成员编码 （01 为户主，其他按入户名单给出顺序依次填写）		01	02	03	04	05	06	07
A11.1	他人代答的原因： (1)本人去外地打工 (2)本人太小 (3)本人头脑不清<br>(4)本人不愿意回答 (5)本人暂时不在家 (6)其他							
A12	与同年龄的人相比，你感觉你现在的健康状况如何?<br>(1)非常好 (2)好 (3)一般 (4)差 (5)非常差 (6)拒绝回答 (7)不知道							
A13	各项身体参数							
A13.1	身高（cm）							
A13.2	体重（kg）							
A13.3	腰围（cm）							
A13.4	臀围（cm）							
**B. 调查前 14 天病伤及就诊情况**								
B1	调查前 14 天内，您是否有身体不适? (1)是 (2)否**（跳问 C）**							
B3.1	您患的是什么病或伤？ **（填疾病名称）（有多种病伤的填写多个病伤名称）**							
B3.2	**（填疾病编码）（根据国家卫生服务调查疾病编码）**							
B4	调查前 14 天内，您因身体不适卧床了多少天？ （天）**（无卧床，填 0）**							
B5	如您是工作/务农人员，因身体不适休工了多少天？ （天）**（无休工，填 0）**							
B6	如您是学生，因身体不适，休学了多少天？（天）**（无休学，填 0）**							
B7	您患病后，是否进行了治疗（包括自我医疗）？ (1)是**（跳问 B8）**(2)否							
B7.1	如未治疗，最主要的原因是什么？**（回答后，跳问 C）**<br>(1)自感病轻 (2)经济困难 (3)无时间 (4)交通不便 (5)无有效措施 (6)其它							
B8	您是如何治疗的？ (1)两周内找医生看过<ins>且进行</ins>自我医疗<br>(2)两周内找医生看过**（跳问 B11）**<br>(3)纯自我医疗							

被调查成员编码 （01为户主，其他按入户名单给出顺序依次填写）		01	02	03	04	05	06	07
B10	如果是自我医疗，您吃的药品来源是：**（最多可选三项）**<br>(1)家里已有的 **（跳问 B11）** (2)药店买的 **（问 B10.11-12）**<br>(4)别人给的 **（跳问 B11）** (5)其它 **（跳问 B11）**							
B10.11	您为什么选择在药店买药而不是去医疗机构就诊？<br>(1)药店的药品种类比医疗机构齐全 (2)自感病轻/没有必要看医生<br>(3)药房买药比医疗机构的药较便宜 (4)没有时间去医疗机构就诊<br>(5)去医疗机构就诊交通不便 (6)医疗机构服务差 (7)其他							
B10.12	若在药店购买，您在这14天内一共购买药品的花费（包括儿女、家人、亲戚等给买药）是多少元?**（自付费用）**							
**下列问题询问两周内就诊情况，两周内未就诊者** *(B8＝(3))*，**结束B部分问题**								
B11	过去14天内您看过几次病？**（次）**							
**下列问题是关于第一次就诊时的情况（由本人或知情人回答）**								
B12	第一次就诊是在哪里？（请填写就诊单位名称，包括私人诊所名称也详细填写）							
opfaclvl-1	**级别 1、村卫生室 2、乡镇卫生院 3、县级医院 4、县级以上医院 5、私人诊所 6 其他**							
B12.1	选择上述单位就诊的最主要原因是：<br>(1)距离近/方便 (2)收费合理 (3)技术水平高 (4)设备条件好 (5)药品丰富<br>(6)服务态度好 (7)定点单位 (8)有熟人 (9)有信赖医生 (10)转诊 (11)其它							
B13	这次就诊，您是否接受了如下治疗？							
B13.1	肌肉注射 (1)是 (2)否							
B13.2	输液治疗 (1)是 (2)否							
B13.3	口服药物治疗 (1)是 (2)否 **（跳问 B13.5）**							
B13.3.1	您吃的药是： (1)中药 (2)西药 (3)中西药都有 (4)不知道							

被调查成员编码 （01 为户主，其他按入户名单给出顺序依次填写）		01	02	03	04	05	06	07
B13.3.2	您吃的药主要是从哪里来的？**（最多可选三项）**<br>(1)就诊机构买的 (2)家里已有的 (3)药店买的 (4)别人给的 (5)其它							
B13.5	您这次就诊是否被转诊到上级医疗机构？ (1)是 (2)否 **（跳问 B13.6）**							
B13.5.1	您被转诊到哪一级医疗机构？<br>(1)乡镇卫生院 (2)县医院 (3)县以上综合医院<br>(4)疾病控制中心（防疫站）结核病门诊 (5)其他							
B13.6	您这次就诊是否被从其他医疗机构转诊过来？ (1)是 (2)否 **(跳问 B14)**							
B13.6.1	在转诊之前，您在哪级医疗机构就诊？<br>(1)村卫生室 (2)乡卫生院 (3)县医院 (4)疾控中心 (5)县级以上的医疗机构 (6)其他							
B14	此次就诊医疗费总额多少元？							
B14.1	此次就诊医疗费用现金自付（包括儿女、家人、亲戚等给支付的）多少元？							
B14.1.1	您是否从医疗保险得到“一站式结算”报销(每一项结算的费用已经减去医保报销部分)？<br>(1)得到报销**(跳问 B16)** (2) 没有得到报销							
B14.1.2	您为什么没有得到报销？<br>(1)就医的时候忘记带社会保障卡 (2)没有参加医疗保险<br>(3)被告知我需要的药和服务不在报销的范围内 (4)药费很便宜，不值得报 (5)其他							
B16	您对此次就诊感到：<br>(1)非常满意 (2)满意 (3)一般满意 (4)不满意 (5)非常不满意<br>**(选择(1)或(2)，跳问 B16.2；选择(3)，跳问 B17；选择(4)或者(5)，回答 B16.1)**							
B16.1	您对此次就诊的医疗机构感到最不满意的是什么？**（最多可选三项）**<br>(2)技术水平低 (3)设备条件差 (4)药品种类少<br>(5)服务态度差 (6)提供不必要服务（包括药品和检查） (7)收费不合理<br>(8)医疗费用高 (9)看病手续烦琐 (10)等候时间过长 (11)其它							

被调查成员编码 （01 为户主，其他按入户名单给出顺序依次填写）		01	02	03	04	05	06	07
B16.2	您对此次就诊的医疗机构感到最满意的是什么？**（最多可选三项）**<br>(2)技术水平高 (3)设备条件好 (4)药品种类多<br>(5)服务态度好 (6)不提供不必要服务（包括药品和检查） (7)收费合理<br>(8)医疗费用低 (9)看病手续简便 (10)等候时间短 (11)其它							
**下列问题是关于第二次就诊时的情况（由本人或知情人回答）**								
B17	第二次就诊是在哪里？**(请填写就诊单位详细名称,包括私人诊所名称也详细填写)**							
opfaclvl-2	级别 ：1、村卫生室 2、乡镇卫生院 3、县级医院 4、县级以上医院 5、私人诊所 6 其他							
B17.1	选择上述单位就诊的最主要原因是：<br>(1)距离近/方便 (2)收费合理 (3)技术水平高 (4)设备条件好 (5)药品丰富<br>(6)服务态度好 (7)定点单位 (8)有熟人 (9)有信赖医生 (10)转诊 (11)其它							
B18	这次就诊后，您是否接受了如下治疗？							
B18.1	肌肉注射 (1)是 (2)否							
B18.2	输液治疗 (1)是 (2)否							
B18.3	口服药物治疗 (1)是 (2)否 **(跳问 B18.5)**							
B18.3.1	您吃的药是： (1)中药 (2)西药 (3)中西药都有 (4)不知道							
B18.3.2	您吃的药主要是从哪里来的？**（最多可选三项）**<br>(1)就诊机构买的 (2)家里已有的 (3)药店买的 (4)别人给的 (5)其它							
B18.5	您这次就诊是否被转诊到上级医疗机构？ (1)是 (2)否 **（跳问 B18.6）**							
B18.5.1	您被转诊到哪一级医疗机构？<br>(1)乡镇卫生院 (2)县医院 (3)县以上综合医院<br>(4)疾病控制中心（防疫站）结核病门诊 (5)其他							
B18.6	您这次就诊是否被从其他医疗机构转诊过来？ (1)是 (2)否 **(跳问 B20)**							
B18.6.1	在转诊之前，您在哪级医疗机构就诊？<br>(1)村卫生室 (2)乡卫生院 (3)县医院 (4)疾控中心 (5)县以外的医疗机构 (6)其他							

被调查成员编码 （01为户主，其他按入户名单给出顺序依次填写）		01	02	03	04	05	06	07
B20	此次就诊医疗费总额多少元？							
B20.1	此次就诊医疗费用现金自付（包括儿女、家人、亲戚等给你支付的）多少元？							
B20.1.1	您是否从医疗保险得到“一站式结算”报销（每一项结算的费用已经减去医保报销部分）？<br>(1)得到报销**(跳问B22)**　(2) 没有得到报销							
B20.1.2	您为什么没有得到报销？<br>(1)就医的时候忘记带社会保障卡　(2)没有参加医疗保险<br>(3)被告知我需要的药和服务不在报销的范围内 (4)药费很便宜，不值得报　(5)其他							
B22	您对此次就诊感到：⑴非常满意 ⑵满意 ⑶一般满意 ⑷不满意 ⑸非常不满意<br>**(选择⑴或⑵，跳问B22.2；选择(3)，跳问C1；选择(4)或者(5)，回答B22.1)**							
B22.1	您对此次就诊的医疗机构感到最不满意的是什么？**（最多可选三项）**<br>(2)技术水平低　(3)设备条件差　(4)药品种类少<br>(5)服务态度差　(6)提供不必要服务（包括药品和检查）　(7)收费不合理<br>(8)医疗费用高　(9)看病手续烦琐　(10)等候时间过长　(11)其它							
B22.2	您对此次就诊的医疗机构感到最满意的是什么？**（最多可选三项）**<br>(2)技术水平高　(3)设备条件好　(4)药品种类多<br>(5)服务态度好　(6)不提供不必要服务（包括药品和检查）　(7)收费合理<br>(8)医疗费用低　(9)看病手续简便　(10)等候时间短　(11)其它							
**C. 一年内住院情况**								
C1	在过去一年中，您在就诊时是否有医生诊断您需要住院的情况？(1)是(2)否**（跳问 D）**							
C1.1	在过去一年一共有几次(医生诊断您需要住院)？							
C2	在过去一年内医生诊断您需要住院而您未住院的次数？<br>**（填具体次数，如没有，填写0并跳问C3）**							
C2.1	未住院主要原因：<br>(1)没必要(2)没时间(3)经济困难(4)服务差(5)价格太高(6)没床位(7)其它							

被调查成员编码 （01 为户主，其他按入户名单给出顺序依次填写）		01	02	03	04	05	06	07
C3	在过去一年里，您一共住过几次医院?（**填具体次数，如没有，填写 0 并跳问 D**）							
C3.1	在过去一年里，您一共住过多少天院？（**填具体天数**）							
**过去一年内最近一次住院情况**								
C4	您因疾病或损伤中毒等住院的疾病名称?<br>**(调查时先填写疾病名称，核查问卷时转填成疾病编码)**							
C4.1.2	**疾病 1**							
C4.1.3	**疾病 2**							
C4.1.4	**疾病 3**							
C4.1	本次住院的入院时间：**（年）**							
C4.1.1	**（月）**							
C4.2	本次住院的医疗机构名称是？							
INPFACLV	级别： **1 乡镇卫生院 2 县级医院 3 县级以上医院 4 其他**							
C4.2.1	本次住院的医疗机构在哪里？ (1)县内 **（回答 C4.3-C4.3.1）**<br>(2)县外 **（回答 C4.2.2—C4.2.4）**							
若 C4.2.1 选择(2)，请回答 C4.2.2-C4.2.4								
C4.2.2	您这次住院选择县外医疗机构的原因是什么？<br>(1)环境条件好 (2)技术水平高 (3)病情重 (4)设备条件好<br>(5)药品种类多 (6)医生推荐 (7)距离我住的地方近 (8)其他							
C4.2.3	您这次到县外医院住院，是否经过县内医院的转诊？ (1)是 (2)否 **（跳问 C4.4）**							
C4.2.4	如转院，您从哪里转院过来的？(1)乡镇卫生院 (2)县医院 (3)县中医院 (4)其他							
若 C4.2.1 选择(1)，请回答 C4.3-C4.3.1								
C4.3	本次住院是否为转院？ (1)是 (2)否（**跳问 C4.4**）							

被调查成员编码 （01 为户主，其他按入户名单给出顺序依次填写）		01	02	03	04	05	06	07
C4.3.1	如转院，您是从哪里转院过来的？<br>(1)乡镇卫生院 (2)县(区)医院 (3)市(地)医院 (4)省级医院<br>(5)部队医院 (6)县中医院 (7)地市以上中医院 (8)私立医院 (9)其他							
C4.4	这次住院的住院天数：（天）							
C4.5	本次住院，您是否做过手术？ (1)是 (2)否							
C4.6	如您在工作/务农，因这次住院而休工的天数？**（包括住院天数，没有填 0）**							
C4.6.1	如您是学生，因这次住院而休学的天数？**（包括住院天数，没有填 0）**							
C4.6.2	这次住院前后在家卧床的天数？**（不包括住院天数，没有填 0）**							
C4.7	您这次出院是由于：(1)病愈医生要求 (2)病未愈医生要求 (3)自己要求 (4)其他原因<br>**(选(3)回答 C4.7.1，其他选项跳问 C4.8)**							
C4.7.1	如您自己要求出院，原因：<br>(1)久病不愈 (2)经济困难 (3)医院条件所限 (4)服务态度不好 (5)其他							
C4.8	过去一年，您是否已参加城乡统筹基本医疗？<br>(1)是 (2)否**（跳问 C4.10）**							
C4.8.1	如果您参加了城乡统筹基本医疗，本次住院您缴纳医疗费用的方式是：<br>(1)自己先全部垫付，再去医保中心报销<br>(2)医院直接减免应报销部分的费用**（跳问 C4.10）**							
如果 C4.8.1 选择(1)，请回答 C4.9 和 C4.9.1；如果 C4.8.1 选择(2)，请回答 C4.10								
C4.9	本次住院医疗费用您自己（包括儿女、家人、亲戚）先垫付了多少元？ **（不包括车费、陪护、红包）**							
C4.9.1	本次住院医疗费用医保中心给你报销了多少元？**（跳问 C4.11）**							
C4.10	本次住院医疗费用您自己（包括儿女、家人、亲戚）支付了多少元？**（不包括报销减免、车费、陪护、红包）**							
C4.11	因本次住院所花费的车旅费、营养伙食费、陪护费共多少（元）？ **（没有填 0）**							

被调查成员编码 （01 为户主，其他按入户名单给出顺序依次填写）		01	02	03	04	05	06	07
C4.13	您对此次住院感到：<br>(1)非常满意 (2)满意 (3)一般满意 (4)不满意 (5)非常不满意<br>**(选择(1)或(2)，跳问 C4.13.2；选择(3)，跳问 C5；选择(4)或者(5)，回答 C4.13.1)**							
C4.13.1	您对住院单位最不满意的是什么？**（最多可选三项）**<br>(2)技术水平低 (3)设备条件差 (4)药品种类少<br>(5)服务态度差 (6)提供不必要服务（包括药品和检查） (7)收费不合理<br>(8)医疗费用高 (9)看病手续烦琐 (10)等候时间过长 (11)就医环境差<br>(12)治疗不彻底或治疗效果不明显 (13)其它							
C4.13.2	您对住院单位最满意的是什么？**（最多可选三项）**<br>(2)技术水平高 (3)设备条件好 (4)药品种类多<br>(5)服务态度好 (6)不提供不必要服务（包括药品和检查） (7)收费合理<br>(8)医疗费用低 (9)看病手续简便 (10)等候时间短 (11)就医环境好<br>(12)治疗彻底或治疗效果明显 (13)其它							
**过去一年内最近一次住院之前一次住院情况（如果在一年内有两次及两次以上住院时间，否则跳问 D）**								
C5	您因疾病或损伤中毒等住院的疾病名称?<br>**(调查时先填写疾病名称，核查问卷时转填成疾病编码)**							
C5.1.2	疾病 1							
C5.1.3	疾病 2							
C5.1.4	疾病 3							
C5.1	本次住院的入院时间：**（年）**							
C5.1.1	**（月）**							
C5.2	本次住院的医疗单位名称是？**（写出详细名称）**							
INPFACLV	级别： 1、乡镇卫生院 2、县级医院 3、县级以上医院 4、其他							
C5.4	这次住院的住院天数： **（天）**							

被调查成员编码 （01 为户主，其他按入户名单给出顺序依次填写）		01	02	03	04	05	06	07
如果 C4.8.1 选择(1)，请回答 C5.9 和 C5.9.1；如果 C4.8.1 选择(2)，请回答 C5.10								
C5.9	本次住院医疗费用您自己（包括儿女、家人、亲戚）先垫付了多少元？ **（不包括车费、陪护、红包）**							
C5.9.1	本次住院医疗费用医保中心给你报销了多少元？**（跳问 C5.11）**							
C5.10	本次住院医疗费用您自己（包括儿女、家人、亲戚）支付了多少元？**（不包括报销减免、车费、陪护、红包）**							
C5.11	因本次住院所花费的车旅费、营养伙食费、陪护费共多少（元）？ **（没有填 0）**							
D. **慢性病情况 （请参照培训手册慢性病列表）**								
D1	过去您是否患有**经医生诊断**的慢性疾病？ (1)是 (2)否**（跳问 E）**							
D1.1.4	**您患有下列哪几种慢性病（可多选）**<br>(1)高血压 (2)糖尿病 (3)椎间盘疾病<br>(4)脑血管病 (5)慢性胃肠炎 (6)冠心病<br>(7)类风湿性关节炎 (8)慢性阻塞性肺疾病 (9)其它________（填写疾病编码）							
D1.1.5	共患有几种慢性病？**（填写个数）**							
D1.1.6	最早确诊的慢性病的患病年限？							
D2	最近三个月内，您因这些疾病看过几次病？ **（如没有，填写 0 并跳问 D3.5）**							
D3.1	最近三个月内，您因这些疾病就诊的主要机构是：<br>(1)村卫生室 (2)乡镇卫生院 (3)县医院 (4)私人诊所 (5)其他							
D3.2	您是否已办理门诊大病医疗证？ (1)是 (2)否**（跳问 D3.4）**							
D3.2.1	如果您参加了城乡统筹，看这些病时您缴纳医疗费用的方式是：<br>(1)自己先全部垫付，再去医保中心报销<br>(2)医院直接减免报销部分的费用 **(选择(2)，跳问 D3.4)**							
D3.3	最近三个月因这些疾病而产生的医疗费用您自己（或者儿女、家人、亲戚）先垫付了多少元？**（不包括车费、陪护、红包）**							

被调查成员编码 （01 为户主，其他按入户名单给出顺序依次填写）		01	02	03	04	05	06	07
D3.3.2	最近三个月因这些疾病而产生的医疗费用医保中心给你报销了多少元？**（跳问 D3.5）**							
D3.4	最近三个月因这些疾病而产生的医疗费用您自己（或者儿女、家人、亲戚）支付了多少元？**（不包括报销减免、车费、陪护、红包）**							
D3.5	最近三个月内，您因这些疾病在药店自行买药（包括儿女、家人、亲戚替你买药）共花费多少元？**（没有填 0）**							
**E. 15 岁及以上成员健康及行为（2007 年及以前出生的人口）（若无此年龄段人群则跳问 F）**								
E1	今天您在行动方面：<br>(1)可以四处走动，无任何困难　(2)行动有些不便　(3)卧病在床							
E2	今天您自我照顾（盥洗和穿衣）方面：<br>(1)无任何问题　(2)有些问题　(3)无法自己盥洗或穿衣服							
E3	今天您从事平常活动（工作、读书或做家务）方面：<br>(1)从事日常活动无任何问题　(2)有些问题　(3)无法从事日常活动							
E4	今天您身体疼痛或不适方面：<br>(1)无任何疼痛或不适　(2)自觉有中度疼痛或不适　(3)自觉极度疼痛或不适							
E5	今天您在焦虑或沮丧方面：<br>(1)不觉得焦虑或沮丧　(2)自觉中度焦虑或沮丧　(3)自觉极度焦虑或沮丧							
E6	请您在刻度尺上，指出最能代表您今天健康状况好坏的那一点<br>0　10　20　30　40　50　60　70　80　90　100<br>最差健康状况　最好健康状况							
E7	总体来讲，您的健康状况是：　(1)非常好　(2)很好　(3)好　(4)一般　(5)差							

被调查成员编码	（01 为户主，其他按入户名单给出顺序依次填写）	01	02	03	04	05	06	07
E8	跟 1 年以前比您觉得自己的健康状况是：<br>(1)比 1 年前好多了 (2)比 1 年前好一些 (3)跟 1 年前差不多<br>(4)比 1 年前差一些 (5)比 1 年前差多了							
I1	吸烟							
I1.1	您吸烟吗？ （1）从未吸**(跳问 I4)**（2）偶尔吸（3）经常吸 （4）已戒烟**（跳问 I1.5）**							
I1.2	您开始吸烟的年龄多大？**（周岁）**							
I1.3	您平均每天吸多少支烟：**（填具体支数）**							
I1.4	请问您每月吸烟大约花多少钱?**(跳问 I4)**							
I1.5	请问您戒烟多长时间**（年数）**							
I1.5.1	戒烟的主要原因**（此题可多选）**？<br>（1）已患病 （2）预防疾病 （3）经济原因 （4）家庭反对 （5）环境限制<br>（6）树立形象 （7）经宣传教育 （8）经医生劝告 （9）其它 （10）不知道							
I4	饮酒							
I1.1	您饮酒吗？ （1）从未喝过**(跳问 I5)**（2）偶尔喝（3）经常喝 （4）已戒酒**（跳问 I4.5）**							
I4.2	您开始喝酒的年龄多大？**（周岁）**							
I4.3	您平均每天喝多少酒：**（填具体斤数）**							
I4.4	请问您每月喝酒大约花多少钱?**(跳问 I5)**							
I4.5	请问您戒酒多少年了？							
I4.5.1	戒酒的主要原因（此题可多选）？<br>（1）已患病 （2）预防疾病 （3）经济原因 （4）家庭反对 （5）环境限制<br>（6）树立形象 （7）经宣传教育 （8）经医生劝告 （9）其它 （10）不知道							
I5	锻炼运动							

被调查成员编码 （01 为户主，其他按入户名单给出顺序依次填写）		01	02	03	04	05	06	07
I5.1	您平均每周有意识的体育锻炼有多少次(包括广场舞、步行锻炼、散步、跑步、早操、课间操、体育课、课外体育班、工间操等)？<br>(1) 6 次及以上 (2) 3-5 次 (3)1-2 次 (4)不到 1 次 (5)从不锻炼（**跳问 M**）							
I5.2	您平均每次的体育锻炼时长有多少？ （1）1 小时以上 （2）30 分钟-1 小时 （3）不足 30 分钟							
**M. 15 岁及以上成员心理健康状况调查：请根据您过去两周的情况，在空格中填写与您情况相符的答案序号。(需本人亲自回答,若本人不在跳问 F)**								
M22	做事时提不起劲或没有兴趣<br>①完全不会 ②有几天 ③超过一周 ④几乎每天							
M23	感到心情低落，沮丧或绝望<br>①完全不会 ②有几天 ③超过一周 ④几乎每天							
M24	入睡困难，睡不安稳或睡眠过多<br>①完全不会 ②有几天 ③超过一周 ④几乎每天							
M25	感觉疲倦或没有活力<br>①完全不会 ②有几天 ③超过一周 ④几乎每天							
M26	食欲不振或吃太多<br>①完全不会 ②有几天 ③超过一周 ④几乎每天							
M27	感觉自己很糟糕或失败，或让自己和家人失望<br>①完全不会 ②有几天 ③超过一周 ④几乎每天							
M28	很难集中注意力做事，例如读报纸、看电视等<br>①完全不会 ②有几天 ③超过一周 ④几乎每天							
M29	动作或说话速度变得非常缓慢，或正好相反，出现烦躁或坐立不安比平时多<br>①完全不会 ②有几天 ③超过一周 ④几乎每天							
M30	有轻生或用某种方式伤害自己的念头<br>①完全不会 ②有几天 ③超过一周 ④几乎每天							

被调查成员编码 （01 为户主，其他按入户名单给出顺序依次填写）		01	02	03	04	05	06	07
F. 15-49 岁已婚育龄期妇女调查表（需要本人亲自回答）（1972-2007）（若无此年龄段人群则跳问 G）								
F1.1	过去一年中，您是否免费做过妇科(乳腺、子宫)检查？ (1)是(2)否							
F1.2	过去一年中，您是否免费做过宫颈检查？**(包括宫颈涂片、TCT、HPV 检查等)**<br>(1)是 (2)否							
F1.3	过去一年中，您是否免费做过乳腺检查？**(包括 B 超、钼靶等)** (1)是(2)否							
F2	您在 2019 年 2 月 1 日以后是否有分娩 (1)是 (2)否**（跳到 G1）**							
F3	最近一次分娩，产前做过几次检查（次）？**(从未做过，填 0，并跳问 F4)**							
F3.1	第一次产前检查是在怀孕第几周时做的？**(周)**							
F3.2	您都在哪里做过产前检查？**（最多可选三项）**<br>(1)县/区及以上医院 (2)县/区及以上中医院 (3)妇幼保健机构<br>(4)乡镇街道卫生院 (5)社区卫生服务中心 (6)计划生育指导站<br>(7)卫生室/所/站 (8)其它							
F4	分娩地点：**（选择(8)以外答案则跳问 F4.3）**<br>(1)县级以上医院 (2)县级医院 (3)妇幼保健机构 (4)乡镇街道卫生院<br>(5)社区卫生服务中心 (6)计划生育指导站 (7)卫生室/所/站 (8)家中 (9)其他							
F4.3	本次分娩您是 (1)顺产 (2)剖宫产 (3)其它							
F6	产后 42 天内，医生或保健员到您家访视过几次？（次）**（没有填写 0）**							
G. 7 岁以下儿童调查表 **（最好由孩子的母亲回答；如果孩子母亲不在现场，则由最了解孩子的情况的人回答）（2015 年以后出生）（若无此年龄段人群则跳问 H）**								
G1	孩子母亲在调查表中的编码？**（如果其母亲未被调查，填回答人的编码）**							
G1.1	孩子是否为母乳喂养 ？ (1)是 (2)否							
G2	近 12 个月内，孩子接受了几次健康体检（次）？**（不包括为治疗疾病而做的检查）**							
G3	孩子有计划免疫接种卡或手册吗？ (1)有 (2)没有 (3)不知道							
G5	通常在哪里进行免疫接种的？<br>(1)疾控中心 (2)卫生院 (3)社区卫生服务中心 (4)村卫生室/所/站 (5)其它							

被调查成员编码 （01 为户主，其他按入户名单给出顺序依次填写）		01	02	03	04	05	06	07
G8	是否接种过其他自费疫苗？<br>(1)是 (2)否 **（跳问 G9）** (3)不知道							
G8.1	接种哪种自费疫苗？							
G9	孩子是否曾被诊断为贫血？ (1)是 (2)否							
G10	孩子是否是留守儿童？ (1)是 (2)否							
**H. 45 岁及以上中老年人口调查表（1977 年及以前出生）（若无此年龄段人群则跳问 J）**								
H11	您洗澡时是否需要他人帮助?<br>(1)自己完全可以 (2)有些困难 (3)需要帮助 (4)根本无法做							
H12	您穿衣时是否需要他人帮助?<br>(1)自己完全可以 (2)有些困难 (3)需要帮助 (4)根本无法做							
H13	您上厕所时是否需要他人帮助?<br>(1)自己完全可以 (2)有些困难 (3)需要帮助 (4)根本无法做							
H14	您在室内活动时是否需要他人帮助?<br>(1)自己完全可以 (2)有些困难 (3)需要帮助 (4)根本无法做							
H15	您能否控制大小便?<br>(1)自己完全可以 (2)有些困难 (3)需要帮助 (4)根本无法做							
H16	您吃饭时是否需要他人帮助?<br>(1)自己完全可以 (2)有些困难 (3)需要帮助 (4)根本无法做							
H17	您洗脸、梳头、刷牙、剃须是否需要他人帮助？<br>(1)自己完全可以 (2)有些困难 (3)需要帮助 (4)根本无法做							
H18	您从床上移动到椅子上是否需要他人帮助？<br>(1)自己完全可以 (2)有些困难 (3)需要帮助 (4)根本无法做							
H19	您上楼梯是否需要他人帮助（上下一端楼梯，用手杖也算独立，可用上台阶代替）<br>(1)自己完全可以 (2)有些困难 (3)需要帮助 (4)根本无法做							

被调查成员编码 （01 为户主，其他按入户名单给出顺序依次填写）		01	02	03	04	05	06	07
**L. 45 岁及以上中老年人口儿童时期情况调查表（1977 年及以前出生）（需要本人亲自回答）（若无此年龄段人群则跳问 J）**								
L1	你的出生地在哪里？（1）本省（2）外省							
L2	儿童青少年期是否挨过饿？（1）是（2）不是（3）不知道							
L3	儿童青少年期生病能否及时得到治疗（去医院或买药）？（1）能（2）不能（3）不知道							
L4	儿童青少年期你身体怎么样？（1）非常好（2）好（3）一般（4）差（5）非常差							
L5	儿童青少年期父母是否都健在？<br>（1）父亲或母亲在（2）双亲在（3）双亲都不在**（跳问 L7）**							
L6.1	儿童青少年期你父亲身体怎么样？（1）非常好（2）好（3）一般（4）差（5）非常差							
L6.2	儿童青少年期你母亲身体怎么样？（1）非常好（2）好（3）一般（4）差（5）非常差							
L7.1	你的父亲是否上过学？（1）上过（2）没上过							
L7.2	你的母亲是否上过学？（1）上过（2）没上过							

**J. 家庭收支情况**	
**家庭基本情况**	
**回答该部分的被调查成员编码是(由户主或知情人回答)：**	
J1 家庭是否拥有下列财产：**(1)是（请填写数量） (2)否 （填 0)**	
J1.2 自行车（电动车）	
J1.5 彩色电视	
J1.7 摩托车	
J1.8 汽车	
J1.9 电冰箱	
J1.10 洗衣机	
J1.11 电话（包括手机、座机）	
J1.12 农用机器	
J1.13 卫星接收器、VCD、DVD、音响等**（有一项即可）**	
J1.14 照相机、摄像机等____________	

被调查成员编码 （01 为户主，其他按入户名单给出顺序依次填写）	01	02	03	04	05	06	07
J1.15 空调							
J1.16 牛							
J1.17 羊							
J1.18 马、驴、骡子							
J1.19 猪**（注意：在登记基本信息时，如果为回民家庭，一定不要问这个问题）**							
J1.20 水浇地							
J1.21 山地							
J1.22 电脑（包括台式和笔记本电脑）							
J1.23 厨房电器用品（微波炉、电磁炉、电炒锅、抽油烟机等）**（填写多少件数）**							
J1.24 太阳能							
J1.25 鸡、鸭等家禽**（填写多少只）**							
**以下问题（J2-J8）请按照现在实际居住的房屋情况回答**							
J2 您家住房类型： (1)砖土混 (2)砖木 (3)土木 (4)全砖（硬顶） (5)窑洞 (6)其他							
J2.1 您家房子的地是什么材质？ (1)土地 (2)砖地 (3)瓷砖 (4)木地板 (5)地板革 (6)其他							
J2.2 您家房子是哪一年盖的？ 年							
J2.3 您家房子多大面积？ 平米							
J3 您家饮水主要类型： (1)自来水 (2)山泉水 (3)手压机井水 (4)窖水 (5)井水 (6)江河湖水 (7)塘沟渠水 (8)其他							
J4 您家厕所类型：(1)水冲式 (2)沼气或三格池式(3)双瓮漏斗式 (4)深坑或免水冲(5)马桶 (6)旱厕 (7)无厕所 (8)其他							
J5 您家通常采用什么燃料做饭（最多选两种）：(1)煤(2)电(3)煤油(4)天然液化气/天然气(5)木头、柴草等(6)木炭(7)其他							
J6 住房是否与厨房分离？ (1)是 (2)否							
J8 您家离最近的医疗点的距离（1 公里＝1000 米；1 里＝500 米）							
J8.1 您家距离最近的村卫生室							
J8.1.1 公里数：							
J8.1.2 最常用的交通方式： (1)步行 (2)公交车 (3)自行车 (4)摩托车 (5)其他（自驾、汽车/私家车）							
J8.1.3 所需要时间： 分钟							

被调查成员编码 （01 为户主，其他按入户名单给出顺序依次填写）	01	02	03	04	05	06	07
J8.2 您家距离最近的乡卫生院							
J8.2.1 公里数：							
J8.2.2 最常用的交通方式： (1)步行 (2)公交车 (3)自行车 (4)摩托车 (5)其他（自驾、汽车/私家车）							
J8.2.3 所需要时间： 分钟							
J8.3 您家距离最近的县医院							
J8.3.1 公里数：							
J8.3.2 最常用的交通方式： (1)步行 (2)公交车 (3)自行车 (4)摩托车 (5)其他（自驾、汽车/私家车）							
J8.3.3 所需要时间： 分钟							
J8.4 离您家距离最近的医疗点是（距离最近）： （1）村卫生室 （2）乡卫生院 （3）县医院							
J9 您家是否被列为建档立卡贫困户或低保户？ (1)否 **(跳问 J10.2)** (2) 是							
J9.1 参加城乡统筹时，您家是否得到参保费用的减免? (1)有得到 (2)没有得到 (3)不清楚							
**家庭借贷情况**							
J10.2 在过去 6 个月中，您家是否由于看病就医向他人借过钱？ (1)是 (2)否							
J10 您家现在是否有欠债？ (1)有 (2)没有（**跳问 J11**） (3)不清楚（**跳问 J11**）							
J10.1 如有欠款，造成欠款的主要原因为：<br>(1)购买食物 (2)盖房修房 (3)看病就医 (4)子女上学 (5)交税费 (6)红白喜事 (7)农业生产 (8) 其它**（注明）**							
J10.1.1 欠债金额多少元？							
J10.1.2 因欠债产生的还款利息多少元？							
J11 您家现在是否借款给别人？(1)是 (2)否（**跳问 J12**） (3)不清楚（**跳问 J12**）							
J11.1 借出金额多少元？							
**生产性支出**							
**J12** 您家去年全家生产所需的**（如养牛，化肥，种子，农用机具等）**支出共多少元？							
**消费性支出（单位：元）**							
J13 通常**一个月**，以下物品的现金支出							

被调查成员编码 （01 为户主，其他按入户名单给出顺序依次填写）	01	02	03	04	05	06	07
J13.1 食品支出（ 粮食，肉类，蔬菜，水果，油，盐，酱，醋，茶，烟，酒等）							
J13.3 日用品，如肥皂，纸，笔等							
J13.4 电费，水费，取暖费，做饭燃料费,电话费，快递费,网费等							
J13.5 房租地租等							
J13.7 交通费（车费、油费）							
J13.8 文化，娱乐活动等							
**J14 过去一年（12 个月），以下物品的现金支出（单位：元）**							
J14.1 衣服							
J14.2 学费，书本，学习用品等（**包括家里在外就读的学生的生活费**）							
J14.3 城乡统筹基本医疗的保费							
J14.4 药品，医疗费等（**只包括自己、家人支付的医药费用金额**）							
J14.5 礼品支出，如嫁妆，新年礼品，结婚礼品，葬礼支出，赠送亲友和朋友支出							
J14.8 各类保险支出（**不包括城乡统筹基本医疗的参保费**）							
J14.9 购买耐用品支出，如手机、电脑、电视，家具，电扇，自行车，摩托车，汽车、冰箱、空调等							
J14.10 盖房和修房、买房							
J14.11 其它(**上述月支出和年支出中都未包括的支出**)							
**储蓄情况（单位：元）**							
J15 去年一年您全家钱够花吗,能剩下多少钱？（**无则填 0**）							
**自产物品情况**							
J16 去年一年**全家消费**的自产物品价值<br>(如果被调查者能够回答消费数量，根据单价乘以数量可以获得金额，但如被调查者无法回答数量， 请告知大概钱数，填在金额栏中；**总产量和销售数量是用来计算消费数量的，如果能回答消费数量则不需要填写这两项**)							

自产物品	总产量（斤）	销售数量（斤）	自己消费数量（斤）
J16.1 小麦			

被调查成员编码 （01 为户主，其他按入户名单给出顺序依次填写）	01	02	03	04	05	06	07
J16.2 玉米							
J16.3 蔬菜							
J16.4 肉食							
其中：猪肉（**注意：如果为回民家庭，一定不要问这个问题）**							
羊肉							
牛肉							
鸡、鸭							
J16.5 蛋类							
J16.6 水果							
J16.7 荞麦							
J16.8 胡麻							
J16.9 土豆							
J16.10 其他							
J16.11 枸杞							
**家庭收入情况**							
J17 过去一年全家收入（元）**（包括外出打工的家庭成员寄回的钱）**							
J17.1 过去一年在外打工的家庭成员寄回的钱财（元）							

K. 个人其他情况 (K1-K8 由户主或知情人统一回答，回答人的成员编码是___________)		01	02	03	04	05	06	07
K3	您是否丧失劳动力？（1）是 （2）否 （3）不知道							
K3.1	您是否为残疾人士？（1）是 （2）否							
K4	您是否有健康档案？（1）是 （2）否 （3）不知道							
K5	您是否享受家庭医生签约服务？（1）是 （2）否 （3）不知道							
K6	近一年内，您是否进行过健康体检？（1）是 （2）否 （3）不知道							
K7	您是否知道“住院先诊疗后付费”制度？（1）是 （2）否 （3）不知道							

**被调查成员编码** （**01为户主，其他按入户名单给出顺序依次填写**）		01	02	03	04	05	06	07
K7.1	近一年内，如果您住过院，您是否享受过“住院先诊疗后付费”制度？<br>**（C3项≥1的回答此题）** （1）是 （2）否 （3）不知道							
K8	近一年内，如果您住过院，是否做过手术？**（C3项≥1的回答此题）**<br>（1）是 （2）否 （3）不知道							
K9	近一年内，您因疾病产生误工天数（包括门诊、住院和慢性病）？**（填具体天数）**							
K10	是否为空巢老年家庭 （1）是 （2）否							
**随访情况**								
J18 家庭随访情况								
J18.1 个人随访情况								

家庭随访情况说明：

1=参加2009，2011，2012，2015，2019，2022调查
2=参加2011，2012，2015，2019，2022调查
3=参加2009，2012，2015，2019，2022调查
4=参加2009，2011，2015，2019，2022调查
5=参加2009，2011，2012，2019，2022调查
6=参加2009，2011，2012，2015，2022调查
7=参加2012，2015，2019，2022调查
8=参加2011，2015，2019，2022调查
9=参加2011，2012，2019，2022调查
10=参加2011，2012，2015，2022调查
11=参加2009，2015，2019，2022调查
12=参加2009，2012，2019，2022调查
13=参加2009，2012，2015，2022调查
14=参加2009，2011，2019，2022调查
15=参加2009，2011，2015，2022调查
16=参加2009，2011，2012，2022调查
17=参加2015，2019，2022调查
18=参加2012，2019，2022调查
19=参加2012，2015，2022调查
20=参加2011，2019，2022调查
21=参加2011，2015，2022年调查
22=参加2009，2019，2022调查
23=参加2009，2015，2022调查
24=参加2009，2011，2022调查
25=参加2009，2012，2022调查
26=参加2011，2012，2022年调查
27=参加2009，2022调查
28=参加2011，2022调查
29=参加2012，2022调查
30=参加2015，2022调查
31=参加2019，2022调查
32=仅参加2022年调查
33=2022没有随访到：碰巧调查期间无人在家
34=2022没有随访到：全家长期外出
35=2022没有随访到：全家已经搬离

个人随访情况：

1 = 随访到　2 = 新增家庭成员　3 = 没有随访到：出嫁/分户　4 = 没有随访到：死亡　5 =没有随访到：长期外出　6 = 没有随访到：其他原因

----------完----------
