# Supplementary material for: A study on the factors influencing the vulnerability of women of childbearing age to health poverty in rural western China
Source: Sci Rep. 2024 Jun 8;14:13219. doi: 10.1038/s41598-024-64070-z (PMC11162415; doi:10.1038/s41598-024-64070-z)
Supplement: Supplementary file 5 — Supplementary Tables. [file 41598_2024_64070_MOESM5_ESM.docx]

**A study on the factors influencing the vulnerability of women of childbearing age to health poverty in rural western China**

**Authors’ information**

Ximin Ma^1,2^, Qi Hu ^2, 3^, Jiahui He ^1, 2^, Chunsheng Li^1,2^, Mingsha Song^1,2^, Youyun Wang^1,2^ and Hui Qiao ^1, 2*^

^1^School of Public Health, Ningxia Medical University, Yinchuan 750004, China,

^2^Key Laboratory of Environmental Factors and Chronic Disease Control, Yinchuan 750004, China,

^3^ School of Humanities and Management, Ningxia Medical University, Yinchuan 750004, China

**^*^ Correspondence**: Hui Qiao.

Email:qiaohui71@163.com

Address:No. 1160 Shengli Street, Xingqing District, Yinchuan, Ningxia, China

Mobile telephone number：13995185918

**Table 7 Comparison of poverty rates and health poverty vulnerability between years (Poverty line ($1.9$))**

| Poverty line | 2019 | 2022 | *χ2* | *P* |
| --- | --- | --- | --- | --- |
| Poverty line (1.9$) |  |  |  |  |
| Poor(%) | 691(21.8) | 501(20.6) | 1.023 | 0.312 |
| Non-poor(%) | 2486(78.2) | 1927(79.4) |  |  |
| Vulnerability(%) | 689(21.7) | 515(21.2) | 0.230 | 0.631 |
| Non-vulnerability(%) | 2488(78.3) | 1913(78.8) |  |  |

**Table 8 Comparison of Poverty Rates and Health Poverty Vulnerability Between Years (Poverty line ($2.15$))**

| Poverty line (2.15$) | 2019 | 2022 | *χ2* | *P* |
| --- | --- | --- | --- | --- |
| Poor(%) | 895(28.2) | 655(27.0) | 0.981 | 0.322 |
| Non-poor(%) | 2282(71.8) | 1773(73.0) |  |  |
| Vulnerability(%) | 959(30.2) | 655(27.0) | 6.828 | 0.009 |
| Non-vulnerability(%) | 2218(69.8) | 1773(73.0) |  |  |

**Table 9** Decomposition of risk factors for vulnerability to health poverty among rural women of childbearing age in 2019 and 2022.(Survey weights not considered)

| Variable | 2019 | | | | 2022 | | | |
| --- | --- | --- | --- | --- | --- | --- | --- | --- |
|  | Poverty line(1.9$) | | Poverty line(2.15$) | | Poverty line(1.9$) | | Poverty line(2.15$) | |
|  | Coefficient | SD | Coefficient | SD | Coefficient | SD | Coefficient | SD |
| Physical capital |  |  |  |  |  |  |  |  |
| Type of housing (reference: brick soil concrete) |  |  |  |  |  |  |  |  |
| brick wood | -0.0004 | 0.0034 | -0.0097^**^ | 0.0046 | 0.0042 | 0.0028 | 0.0048 | 0.0048 |
| Civil Engineering | 0.0042 | 0.0055 | -0.0113 | 0.0074 | -0.0064 | 0.0083 | -0.0206 | 0.0141 |
| full brick | -0.0023 | 0.0035 | -0.0278^***^ | 0.0047 | 0.0080^***^ | 0.0028 | 0.0071 | 0.0047 |
| Type of drinking water (reference: tap water) |  |  |  |  |  |  |  |  |
| cellar water | 0.0131^***^ | 0.0032 | 0.0185^***^ | 0.0042 | 0.0323^***^ | 0.0063 | 0.0583^***^ | 0.0109 |
| well water | 0.0163^**^ | 0.0072 | 0.0278^***^ | 0.0096 | 0.0412^***^ | 0.0089 | 0.0838^***^ | 0.0169 |
| Type of toilet(reference: water flushing type) |  |  |  |  |  |  |  |  |
| toilet | -0.0009 | 0.0066 | 0.0036 | 0.0087 | 0.0009 | 0.0050 | 0.0025 | 0.0086 |
| dry toilet | 0.0097^*^ | 0.0053 | 0.0219^***^ | 0.0069 | -0.0010 | 0.0034 | -0.0005 | 0.0057 |
| Separation of housing and kitchen(reference: no) | -0.0087^***^ | 0.0026 | -0.0142^***^ | 0.0035 | 0.0036^*^ | 0.0020 | 0.0068^*^ | 0.0035 |
| Financial capital |  |  |  |  |  |  |  |  |
| Registered poor household(reference:no) | 0.0015 | 0.0024 | -0.0040 | 0.0032 | 0.0070^***^ | 0.0018 | 0.0138^***^ | 0.0031 |
| Household loans(reference:no) | -0.0167^***^ | 0.0028 | -0.0148^***^ | 0.0037 | -0.0065^***^ | 0.0019 | -0.0155^***^ | 0.0033 |
| Annual per capita household income(reference: I group) |  |  |  |  |  |  |  |  |
| II group | -0.8111^***^ | 0.0038 | -0.5250^***^ | 0.0053 | -0.8622^***^ | 0.0028 | -0.5354^***^ | 0.0049 |
| III group | -0.9809^***^ | 0.0039 | -1.0192^***^ | 0.0054 | -0.9863^***^ | 0.0029 | -0.9960^***^ | 0.0050 |
| IV group | -0.9727^***^ | 0.0040 | -1.0099^***^ | 0.0056 | -0.9807^***^ | 0.0030 | -0.9880^***^ | 0.0052 |
| V group | -0.9719^***^ | 0.0041 | -1.0071^***^ | 0.0057 | -0.9761^***^ | 0.0031 | -0.9801^***^ | 0.0054 |
| Social capital |  |  |  |  |  |  |  |  |
| Expenditures on social interactions(log) | -0.0188^***^ | 0.0009 | -0.0249^***^ | 0.0012 | -0.0119^***^ | 0.0007 | -0.0187^***^ | 0.0011 |
| Human capital |  |  |  |  |  |  |  |  |
| Educational attainment of the respondents(reference: no schooling) |  |  |  |  |  |  |  |  |
| primary school | -0.0083^***^ | 0.0030 | -0.0170^***^ | 0.0041 | -0.0058^**^ | 0.0023 | -0.0044 | 0.0040 |
| junior high school | -0.0126^***^ | 0.0035 | -0.0284^***^ | 0.0047 | -0.0068^**^ | 0.0027 | -0.0088^*^ | 0.0046 |
| senior high school or above | -0.0201^***^ | 0.0051 | -0.0413^***^ | 0.0068 | -0.0108^***^ | 0.0037 | -0.0160^**^ | 0.0063 |
| Self-assessed health status of respondents(reference: very good) |  |  |  |  |  |  |  |  |
| good | -0.0158^***^ | 0.0046 | -0.0184^***^ | 0.0062 | -0.0002 | 0.0029 | -0.0034 | 0.0050 |
| average | -0.0161^***^ | 0.0051 | -0.0156^**^ | 0.0068 | 0.0063^**^ | 0.0029 | 0.0070 | 0.0049 |
| poor | -0.0239^***^ | 0.0058 | -0.0214^***^ | 0.0078 | 0.0097^***^ | 0.0036 | 0.0128^**^ | 0.0061 |
| very poor | -0.0142 | 0.0130 | 0.0053 | 0.0177 | 0.0210^***^ | 0.0056 | 0.0358^***^ | 0.0096 |
| Chronic disease of the respondents(reference: no) | -0.0008 | 0.0036 | 0.0024 | 0.0048 | 0.0033 | 0.0030 | 0.0219^***^ | 0.0051 |
| Outpatient service utilization of the respondents(reference: no) | 0.0084^*^ | 0.0049 | 0.0184^***^ | 0.0065 | -0.0067 | 0.0051 | -0.0242^***^ | 0.0086 |
| Inpatient service utilization of the respondents(reference: no) | -0.0008 | 0.0040 | -0.0078 | 0.0052 | -0.0078^**^ | 0.0031 | -0.0113^**^ | 0.0054 |
| Age of the respondents(reference:) | -0.0006^***^ | 0.0002 | -0.0010^***^ | 0.0002 | -0.0007^***^ | 0.0001 | -0.0010^***^ | 0.0002 |
| Occupation of the respondents(reference:Farming ) |  |  |  |  |  |  |  |  |
| working | -0.0024 | 0.0030 | -0.0066 | 0.0040 | -0.0025 | 0.0023 | -0.0061 | 0.0039 |
| unemployed | -0.0070^*^ | 0.0038 | -0.0109^**^ | 0.0051 | -0.0015 | 0.0034 | -0.0111 ^*^ | 0.0059 |

****P*< 0.01; ***P* <0.05; **P* < 0.1.

**Table 10** Decomposition of risk factors for vulnerability to health poverty among rural women of childbearing age in 2019 and 2022.(Survey weights not considered)

| Variable | 2019 | | | | 2022 | | | |
| --- | --- | --- | --- | --- | --- | --- | --- | --- |
|  | Poverty line (1.9$) | | Poverty line (2.15$) | | Poverty line (1.9$) | | Poverty line (2.15$) | |
|  | Shapley | Contribution (%) | Shapley | Contribution (%) | Shapley | Contribution (%) | Shapley | Contribution (%) |
| Type of housing | 1.9153 | 6.42 | 1.4064 | 5.71 | 0.1539 | 0.47 | 0.1073 | 0.41 |
| Type of drinking water | 0.0363 | 0.12 | 0.0320 | 0.13 | 0.3195 | 0.98 | 0.2114 | 0.81 |
| Household loans | 2.0361 | 6.82 | 1.5794 | 6.41 | 1.1934 | 3.65 | 0.9406 | 3.58 |
| Annual per capita household income | 13.0290 | 43.67 | 11.3546 | 46.10 | 13.3036 | 40.69 | 11.5527 | 44.03 |
| Expenditures on social interactions (log) | 10.1361 | 33.97 | 8.2467 | 33.48 | 11.4958 | 35.16 | 9.1860 | 35.01 |
| Educational attainment of the respondents | 0.4066 | 1.36 | 0.3060 | 1.24 | 0.5108 | 1.56 | 0.3850 | 1.47 |
| Self-assessed health status of respondents | 0.9950 | 3.33 | 0.7746 | 3.14 | 0.0722 | 0.22 | 0.0625 | 0.24 |
| Outpatient service utilization of the respondents | 0.0961 | 0.32 | 0.0801 | 0.33 | 0.1245 | 0.38 | 0.0741 | 0.28 |
| Age of the respondents | 1.0726 | 3.60 | 0.8494 | 3.45 | 4.1593 | 12.72 | 3.2647 | 12.44 |
| TOTA | 29.8365 | 100.00 | 24.6292 | 100.00 | 32.6914 | 100.00 | 26.2408 | 100.00 |

**Table 11** Decomposition of different dimensions of vulnerability to health poverty(Survey weights not considered)

| Dimension | 2019 | | | | 2022 | | | |
| --- | --- | --- | --- | --- | --- | --- | --- | --- |
|  | Poverty line (1.9$) | | Poverty line (2.15$) | | Poverty line (1.9$) | | Poverty line (2.15$) | |
|  | Shapley | Contribution (%) | Shapley | Contribution (%) | Shapley | Contribution (%) | Shapley | Contribution (%) |
| Physical capital | 1.9516 | 6.54 | 1.4384 | 5.84 | 0.4734 | 1.45 | 0.3187 | 1.22 |
| Financial capital | 15.0652 | 50.49 | 12.9340 | 52.51 | 14.4970 | 44.34 | 12.4933 | 47.61 |
| Social capital | 10.1361 | 33.97 | 8.2467 | 33.48 | 11.4958 | 35.16 | 9.1860 | 35.01 |
| Human capital | 2.5703 | 8.61 | 2.0101 | 8.16 | 4.8668 | 14.88 | 3.7862 | 14.43 |
